# Estimating effective population size trajectories from time-series identity-by-descent segments

**DOI:** 10.1093/genetics/iyae212

**Published:** 2025-01-24

**Authors:** Yilei Huang, Shai Carmi, Harald Ringbauer

**Affiliations:** Department of Archaeogenetics, Max Planck Institute for Evolutionary Anthropology, Leipzig 04317, Germany; Bioinformatics Group, Institute of Computer Science, Universität Leipzig, Leipzig 04109, Germany; Braun School of Public Health and Community Medicine, Hebrew University of Jerusalem, Jerusalem 9112102, Israel; Department of Archaeogenetics, Max Planck Institute for Evolutionary Anthropology, Leipzig 04317, Germany

**Keywords:** population genetics, evolutionary biology, ancient DNA, bioinformatics, statistical genetics

## Abstract

Long, identical haplotypes shared between pairs of individuals, known as identity-by-descent (IBD) segments, result from recently shared co-ancestry. Various methods have been developed to utilize IBD sharing for demographic inference in contemporary DNA data. Recent methodological advances have extended the screening for IBD segments to ancient DNA (aDNA) data, making demographic inference based on IBD also possible for aDNA. However, aDNA data typically have varying sampling times, but most demographic inference methods for modern data assume that sampling is contemporaneous. Here, we present Ttne (Time-Transect Ne), which models time-transect sampling to infer recent effective population size trajectories. Using simulations, we show that utilizing IBD sharing in time series increased resolution to infer recent fluctuations in effective population sizes compared with methods that only use contemporaneous samples. To account for IBD detection errors common in empirical analyses, we implemented an approach to estimate and model IBD detection errors. Finally, we applied Ttne to two aDNA time transects: individuals associated with the Copper Age Corded Ware Culture and Medieval England. In both cases, we found evidence of a growing population, a signal consistent with archaeological records.

## Introduction

Published ancient DNA (aDNA) data have surged in recent years. This growing dataset now enables researchers to track demographic changes and evolution over time (e.g. Patterson *et al.* 2022; Allentoft *et al.* 2024a), which was previously only feasible for a few model organisms with short generation time (e.g. Burke *et al.* 2010; Bergland *et al.* 2014) or for well-documented wild populations in long-term field projects (e.g. Chen *et al.* 2019; Lamichhaney *et al.* 2020).

Several computational methods were developed to infer the demographic history of a population, including its population size dynamics, based on genomic data from a sample of contemporary individuals (Browning and Browning 2015; Fournier *et al.* 2023). However, most of these methods cannot explicitly model time-series genomic data. Consequently, although many ancient DNA studies have time-series genomes from a single site or region, the analyses at the temporal dimension are often only descriptive, such as tracking ancestry compositions or allele frequency changes over time (e.g. Mathieson *et al.* 2015; Gretzinger *et al.* 2022; Patterson *et al.* 2022; Posth *et al.* 2023). Although these approaches offer easily interpretable population descriptions and provide evidence of migration, admixture, or selection, they fail to fully utilize the temporal information to infer key population genetic parameters. Our method presented here is in line with several recent developments that directly model the time dimension to study important aspects of evolution (e.g. Joseph and Pe’er 2019; Buffalo and Coop 2020; Mathieson and Terhorst 2022).

Identity-by-descent (IBD) segments are long genomic stretches shared by pairs of individuals that result from recent genealogical connections. Because recombination events rapidly break long IBD segments apart, IBD sharing represents an ideal signal for investigating recent demography. Recent methodological advances have enabled inferring IBD segments in aDNA datasets (e.g. the tool Ancibd (Ringbauer *et al.* 2023) or using Ibdseq (Browning and Browning 2013) as in Allentoft *et al.* 2024b). Several methods have utilized IBD sharing in modern populations to estimate various aspects of recent population structure (e.g. Palamara *et al.* 2012; Palamara and Pe’er 2013; Ralph and Coop 2013; Browning and Browning 2015; Ringbauer *et al.* 2017; Nait Saada *et al.* 2020; Cai *et al.* 2023). In addition, a recent method designed for aDNA, HapNe (Fournier *et al.* 2023), can estimate recent effective population size ($N_{e}$) trajectories from either IBD or linkage disequilibrium (LD). However, all these methods assume that all samples are contemporaneous or make approximations when samples are temporally stratified. As a result, if samples span a wide time interval, bias may arise due to assuming time homogeneity.

Here, we present Ttne, a method to estimate recent effective population size ($N_{e}$) trajectories from time-series aDNA data based on IBD segments ≥8 centimorgan (cM) long. Ttne explicitly models IBD sharing in samples from different time points and directly utilizes this temporal dimension to improve the estimate of $N_{e}$ trajectory. Our simulations show that Ttne can accurately estimate recent $N_{e}$ while avoiding overfitting using a regularization scheme. We compared the performance of IBD-based recent $N_{e}$ estimates under two sampling strategies: contemporaneous vs. temporally stratified samples, demonstrating that the latter yields substantially more accurate $N_{e}$ estimates, especially when there is major demographic turnover over a short period. Finally, we apply Ttne to individuals associated with the Corded Ware culture of the European Copper Age and individuals in the British Isles from the Anglo-Saxon migration to the late Medieval period. We found evidence of population growth in both cases, consistent with archaeological and historical records.

## Materials and methods

Here, we describe our model for estimating population size trajectories using IBD sharing in time-series data. First, we derive the theoretical IBD length distribution under a given demography. Our inference scheme then maximizes the composite likelihood of observed IBD length distributions with respect to demography.

**Fig. 1.**
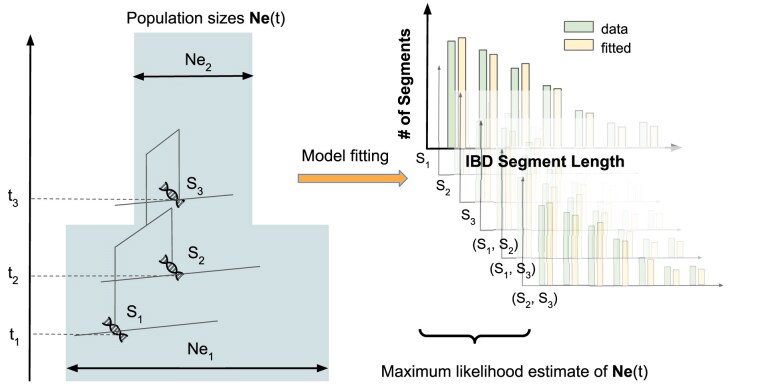
Schematics of using time-series IBD segments for demographic inference. We show a simplified graphical representation of the Ttne model: It assumes multiple sets of samples $S_{i}$ collected from different time points $t_{i}$. Ttne then fits a $N_{e}$ vector to match the observed IBD sharing between samples within each sample set and across pairs of sample sets.

Throughout, we assume a panmictic population with varying population sizes $N_{e}$ over time. We use the bold font $N_{e}$ to denote a vector of floating-point values representing $N_{e}(t)$ at each generation *t* backward in time. We assume we have samples (i.e. sets of individual genomes) from *n* different time points and denote the set of samples in each time point $t_{i}$ (measured by number of generations backward in time with respect to the most recent sample) by $S_{i}$ with sample size $n_{i}$. Moreover, we denote the set of sample sets by $S=\{S_{1},\ldots ,S_{n}\}$, and the set of sampling times by $T=\{t_{1},\ldots ,t_{n}\}$ (Fig. 1). Our model assumes that all IBD segments longer than a length cutoff *l* have their most recent common ancestors within *G* generations predating the oldest samples. This approximation has been applied in several previous IBD-based demographic inference schemes (e.g. Palamara *et al.* 2012; Browning and Browning 2015) and, for sufficiently large *G*, it becomes highly accurate. Thus, we aim to infer $N_{e}$, a vector of length $T_{\max}=G+\max(T)$.

### IBD distribution under a given demography

Throughout, we use the genetic map in Morgans to measure the lengths of IBD segments and chromosomes because this natural recombination distance simplifies the calculations. Due to recombination, a chromosome is cut into an increasing number of segments as one traces its ancestry backward through time. As a result, each chunk of the chromosome follows a different genealogical path. Here, we define an IBD segment as a region where all sites follow the same genealogical path to the most recent common ancestor.

We follow a long-standing analytical framework (Palamara *et al.* 2012; Palamara and Pe’er 2013; Ralph and Coop 2013; Browning and Browning 2015), adapting the specific approach of Ringbauer *et al.* (2017): The expected number of IBD segments of length *l* originating from *t* generations backward in time, $f_{N}(l,t)$, can be expressed as the product of two factors:


(1) $$f_{N}(l,t)=f_{K}(l,t)\phi (t),$$

where the first factor $f_{K}(l,t)$ denotes the expected number of chromosome segments of length *l* after the chromosome has experienced 2t meioses (where each recombination event starts a new segment). Tracing two lineages back to the common ancestor *t* generations ago leads to the total branch length of 2t. The second factor ϕ(t) denotes the standard single-locus coalescent time density. Throughout, functions denoted by f(⋅) are densities, i.e. the quantity they describe (usually the expected numbers of IBD segments) is obtained by integrating (or approximated by performing infinitesimal summation) the density over an interval (e.g. over IBD length and time interval). It is not a probability measure because it does not integrate to 1. Equation (1) holds because the number of IBD segments coming from generation *t* is the number of chromosome pieces multiplied by the single locus probability of coalescence of each potential IBD segment.

**Fig. 2.**
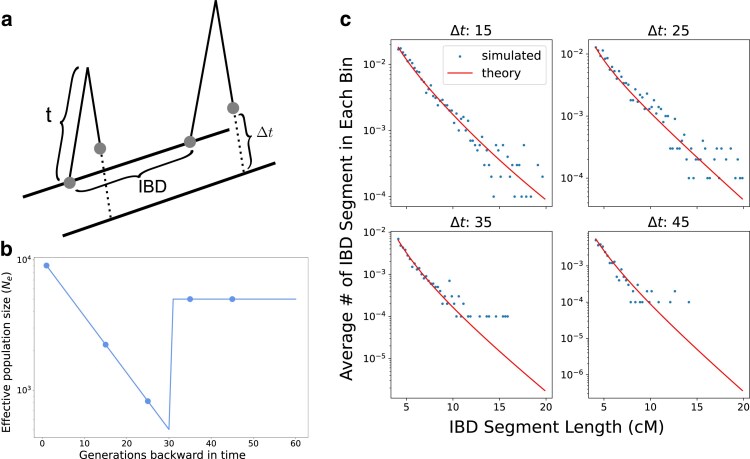
IBD segment sharing in temporally structured samples. a) Schematic of sampling temporally structured haplotypes. The two sampled haplotypes are separated by Δt generations. b) We simulated a bottleneck demography to verify our model and sampled at t=0,15,25,35,45 generations ago. c) To test the correctness of equation (4), we took pairs of haplotypes in each simulated replicate where one is sampled at t=0 and the other at t=15,25,35,45 generations ago (so that Δt=15,25,35,45). We visualized the number of IBD segments in each bin (bin size of 0.25 cM, averaged over 10,000 replicates simulated with different random seeds) as dots and our model prediction as lines. A version of this figure depicting error bars is shown in Supplementary Fig. 16.

For any two haplotypes $h_{1}\in S_{i},h_{2}\in S_{j}$, the time difference is $\Delta t=|t_{i}-t_{j}|$ (Fig. 2a), where $t_{i},t_{j}$ are the times when people with the genomes i,j have lived. Assuming a Poisson model of recombination along a chromosome of length *L*, for any $t>\max(t_{i},t_{j})$, one can extend equation 4 from Ringbauer *et al.* (2017), by updating the total branch length to $t^{*}=2t-\Delta t$:


(2) $$f_{K}(l,t)=\underset{i}{\underbrace{2t^{*}e^{-t^{*}l}}}+\underset{ii}{\underbrace{(L-l)(t^{*}{)}^{2}e^{-t^{*}l}}}.$$

Part (i) describes IBD segments ending in one of the two ends of the chromosome. Because the total branch length up to the most recent common ancestor is $t^{*}$, after fixing one border of the IBD segment to the chromosome end, the distance to the other border of IBD follows an exponential distribution with rate $t^{*}$. Therefore, the probability of having a segment of length *l* at either of the chromosome ends is $t^{*}e^{-t^{*}l}$.

Part (ii) of equation (2) describes IBD segments in the interior of a chromosome. Recombination happens with probability $t^{*}\Delta L$ within a small interval ΔL, and once a recombination happens, the distance to the next recombination event follows an exponential distribution with rate $t^{*}$. Therefore, the probability of obtaining a segment of length *l* that begins from a small interval ΔL is $t^{*}e^{-t^{*}l}t^{*}\Delta L$. Adding up the terms from all possible starting intervals of a segment of length *l* (which sum up to a total length L−l) results in part (ii) in $f_{K}(l,t)$.

We note that equation (2) directly follows from equation 6 in Ralph and Coop (2013), which gives the mean number of pieces of length at least *x* obtained by cutting the chromosome at the recombination sites of $n=t^{*}$ meioses. This quantity is related to $f_{K}(l,t)$ by integrating $f_{K}(l,t)$ from l=x to the length of the chromosome. Differentiating equation 6 from Ralph and Coop (2013) with respect to *l* (and flipping the sign), yields equation (2).

For a single panmictic population with time-varying effective sizes $N_{e}$, the single-locus coalescent probability ϕ(t) is


(3) $$\phi (t)=\frac{1}{2N_{e}[t]}\prod_{i=1+\max(t_{i},t_{j})}^{t-1}\left(1-\frac{1}{2N_{e}[i]}\right),t>\max\left(t_{i},t_{j}\right).$$

To obtain $f_{N}(l)$, the expected number of IBD segments of length *l* between a pair of haplotypes, we sum up $f_{N}(l,t)$ over *t* from $\max(t_{i},t_{j})$ to *G* generations into the past:


(4) $$f_{N}(l)=\sum_{t=\max(t_{i},t_{j})}^{G+\max(t_{i},t_{j})}f_{K}(l,t)\phi (t).$$

For some demographic scenarios, inserting a continuous-time approximation of equation (3) into equation (4) and integrating can yield closed-form results. For instance, for a constant population size $N_{e}$, ϕ(t) is an exponential random variable with rate $\frac{1}{2N_{e}}$. Integrating from Δt to ∞ then yields a closed-form formula analogous to equation (4):


(5) $$\begin{array}{rl} \;f_{N}(l) & \;=\int_{\Delta t}^{\infty }f_{K}(l,t)\frac{1}{2N_{e}}e^{-\frac{t-\Delta t}{2N_{e}}}dt\\ & =\frac{e^{-l\Delta t}}{(1+4N_{e}l{)}^{3}}\left(8N_{e}(1+4N_{e}L)+2\Delta t(1+4N_{e}L)(1+4N_{e}l\right)\\ & \;\;-(L-l)(\Delta t+4N_{e}l\Delta t{)}^{2}). \end{array}$$

To verify this theoretical framework, we used Msprime (Baumdicker *et al.* 2022) to simulate chromosome 3 of a panmictic population with an instantaneous bottleneck at 30 generations backward in time, followed by exponential growth (illustrated in Fig. 2b). We then obtained the simulated IBD sharing rate between one haplotype sampled at present (t=0) and the others sampled at t=15,25,35,45 generations ago using the ibd_segment() function in tskit’s Python API (Kelleher *et al.* 2024). We found that the simulated IBD rates closely match the theoretical predictions, demonstrating the accuracy of our framework (Fig. 2c).

### Calculating the composite likelihood of observed IBD segments given a demography

As described in Ringbauer *et al.* (2017), the expected number of IBD segments whose length falls into a small length bin $\left[l,l+\Delta_{l}\right]$ shared between a pair of haplotypes is approximated accurately by $f_{N}(l+\frac{\Delta_{l}}{2})\Delta_{l}$. Within each length bin, we model the number of IBD segments as a Poisson variable with mean $n_{hap}f_{N}(l+\frac{\Delta_{l}}{2})\Delta_{l}$, where $n_{hap}$ is the number of all possible haplotype pairs.

Assuming that each length bin is independent, the total likelihood is the product of Poisson likelihoods over all length bins. We use *m* equally spaced ($\Delta_{l}$) length bins from $l_{\min}$ to $l_{\max}$ with boundaries $l_{\min}=l_{0}<l_{1}<\cdots <l_{m}=l_{\max}$, and denote the number of observed IBD segments between sample sets $S_{i},S_{j}$ in the $k^{th}$ length bin $\left[l_{k-1},l_{k}\right]$ to be $n_{k}^{\left(i,j\right)}$. Then the log-likelihood $L(S_{i},S_{j}\;|\;N_{e})$ of IBD segments shared between $S_{i}$ and $S_{j}$ is given by:


(6) $$L(S_{i},S_{j}\;|\;N_{e})=\sum_{k=1}^{m}\text{Poisson}\left(n_{k}^{\left(i,j\right)}\right|n_{hap}f_{N}\left(\frac{l_{k-1}+l_{k}}{2}\right)\Delta_{l}),$$

where Poisson(⋅|λ) is the Poisson likelihood with mean *λ*. Note that $f_{N}(\frac{l_{k-1}+l_{k}}{2})$ is conditioned on $N_{e}$, although we did not write out this dependency here in keeping with the notation from the previous section.

It then follows that the composite likelihood of the observed IBD segments among all the sample sets $S=\{S_{1},\ldots ,S_{n}\}$ is:


(7) $$L(N_{e})=\underset{i}{\underbrace{\sum_{i=1}^{n}L(S_{i},S_{i}\;|\;N_{e})}}+\underset{ii}{\underbrace{\sum_{i<j,1\leq i<n,1<j\leq n}L(S_{i},S_{j}\;|\;N_{e})}}.$$

Part (i) gives the likelihood of IBD sharing within each set of samples, and part (ii) gives the likelihood of IBD sharing across all pairwise sets of samples. The summands in equation (7) are not independent, as implicitly assumed when summing them to an overall likelihood. However, we show by simulations that this composite likelihood approximation works well in practice, and it has been used successfully in various other population genetic applications (e.g. Pritchard *et al.* 2000; Alexander *et al.* 2009; Ringbauer *et al.* 2017).

To avoid overfitting, we employed a regularization scheme partly inspired by DeWitt *et al.* (2021), which uses $\ell_{1}$ norms of the first-order and third-order time derivatives of $N_{e}$. Our early exploratory experiments showed that $\ell_{1}$ norm performed less well than $\ell_{2}$ norm for our optimization problems. Therefore, we use quadratic penalty functions.

Our penalty term consists of two parts. For the first term, we use the Hodrick–Prescott filter (H-P filter, Hodrick and Prescott 1997), which is the sum of the squares of the second difference,


(8) $$\text{Penalty1}(x)=\sum_{t=1}^{T_{\max}-1}(x[t-1]-2x[t]+x[t+1]{)}^{2}.$$

This term discourages solutions with large curvature.

The second penalty term is the sum of the square of the first difference with Gaussian positional decay, which is related to the first derivative:


(9) $$\text{Penalty2}(x)=\sum_{t=1}^{T_{\max}}w_{t}\;*\;{\left|x[t]-x[t-1]\right|}^{2},$$

where


(10) $$\begin{array}{rl} w_{t} & =\frac{N(t-T_{\max}\;|\;\mu =0,\sigma =T_{\max}/3)}{N(0\;|\;\mu =0,\sigma =T_{\max}/3)}\\ & =\exp\;\left(-\frac{9(t-T_{\max}{)}^{2}}{2T_{\max}^{2}}\right), \end{array}$$

and N(⋅|μ,σ) denotes a standard Gaussian density with mean *μ* and standard deviation *σ*. We chose $\sigma =T_{\max}/3$ because 99.7% of the probability mass of Gaussian distribution lies within 3σ from its mean. Therefore, $w_{t}$ is nearly zero for *t* close to zero while it is close to one for *t* close to $T_{\max}$. Qualitatively, this term penalizes changes in $N_{e}$ deeper in time where signals from IBD diminish. Our simulations confirm that this term helps stabilize the $N_{e}$ estimate at deeper time depths (Supplementary Fig. 22).

The full objective function of the optimization becomes:


(11) $$\begin{array}{rl} \text{Objective}(N_{e}) & =-L(N_{e})+\alpha \;*\;\text{Penalty1}(\log(N_{e}))\\ & \;+\beta \;*\;\text{Penalty2}(\log(N_{e})). \end{array}$$

This regularization scheme is similar to several previous works (e.g. Ralph and Coop 2013; DeWitt *et al.* 2021). As in DeWitt *et al.* (2021), we apply a log transform before computing the penalty terms because population size can vary over orders of magnitude during expansions and bottlenecks.

This regularization has two parameters, *α*, and *β*, that determine the relative strength of the regularization penalties. In practice, we found that different situations may entail different values of *α* to avoid overfitting. Unless otherwise stated, we fix β=250 as we found that this setting works well across different scenarios (Supplementary Fig. 21). We adapt *α* automatically using cross-validation (described in Supplementary Note 1 and see Fig. 1).

### Inference

To obtain the maximum-likelihood estimator $\hat{N_{e}}$ of the penalized composite likelihood,


(12) $$\hat{N_{e}}=\underset{N_{e}}{arg\;min}\;\text{Objective}(N_{e}),$$

we use the L-BFGS-B algorithm (Zhu *et al.* 1997) as implemented in SciPy (Virtanen *et al.* 2020) to iteratively minimize the objective function equation (11).

To initialize the optimization, we first obtain a maximum-likelihood estimator ${\hat{N_{e}}}^{\mathrm{const}}$ assuming a constant $N_{e}$ using equation (5). The constant $N_{e}$ estimate is fit similarly using L-BFGS-B starting from initial value $N_{e}=1,000$. We then initialize $N_{e}(t)$, the population size trajectory, by:


(13) $$N_{e}(t)={\hat{N_{e}}}^{\mathrm{const}}+\epsilon_{t};\;\epsilon_{t}∼N\left(0,\frac{{\hat{N_{e}}}^{const}}{20}\right).$$

This random Gaussian noise helps the optimization algorithm explore a fuller search space.

To obtain confidence intervals for $N_{e}$, we bootstrap over chromosomes (sampling with replacement from the original 22 autosomes) 200 times and record the 2.5% and 97.5% percentile of each component of $N_{e}$ as the confidence intervals.

Because we calculate the likelihood for all pairs of sample sets, the runtime scales quadratically with the number of sample sets. While the optimization step of Ttne is slower than that of hapNe-IBD, the runtime remains manageable for typical CPUs (Supplementary Fig. 30, e.g. the two main applications described in this manuscript finished within a few CPU-hours).

### Computing posterior distribution of TMRCA

After $N_{e}$ is inferred, obtaining information about the time depth of which IBD segments are informative is helpful. Towards this end, we calculate the time distribution to the most recent common ancestor (TMRCA) of an IBD segment of length *l*. Given $N_{e}$, we can compute the posterior distribution of the TMRCA *T* by applying Bayes’s theorem:


(14) $$\begin{array}{rl} P(T=t\;|\;l,N_{e}) & =\frac{P(l\;|\;T=t)P(T=t\;|\;N_{e})}{P(l\;|\;N_{e})}\\ & \propto P(l\;|\;T=t)P(T=t\;|\;N_{e}), \end{array}$$

where P(l|T=t) denotes the probability density of having an IBD segment of length *l* spanning a chosen marker and originating from *t* generations ago, which is equation (2). An accurate approximate expression that does not consider chromosome edges used in earlier work (e.g. Palamara *et al.* 2012; Baharian *et al.* 2016) is given by:


(15) $$P(l\;|\;T=t)=(2t{)}^{2}le^{-2tl}.$$

The above density is Erlang-2, because the distance to the next recombination on each side of the focal marker is exponential with rate 2t (the total branch length to the common ancestor). Conditioning on a fixed time *t* to the most recent common ancestor, the IBD length distribution is independent of $N_{e}$ because the distance to the next recombination only depends on the recombination events over the time of 2t, as shown in Palamara *et al.* (2012). When Δt generations separate two samples from different generations, this term becomes


(16) $$P(l\;|\;T=t)=(2t-\Delta t{)}^{2}le^{-l(2t-\Delta t)},$$

where *t* is measured from the younger sample. The approximate expression equation (16) gives nearly identical result as the exact expression in practice (Supplementary Fig. 20), so we use equation (16) in our implementation because the exact formula would require computing the posterior for each of the autosomes separately.

Finally, the second term $P(T=t\;|\;N_{e})$ in equation (14) is given by equation (3). We verified this formula with simulations (Supplementary Fig. 20). Our implementation of Ttne computes and visualizes this posterior distribution using the inferred $\hat{N_{e}}$ and its associated cumulative density function (see Supplementary Fig. 18 for one example).

### Modeling IBD detection error

Detecting IBD segments in genomic data is a complex task. Therefore, inferred IBD segments include errors such as false positives, imperfect recall, and length biases, affecting the downstream demographic inference. Here, we use a model similar to Ralph and Coop (2013); Ringbauer *et al.* (2017) to account for these three types of errors. Denoting the theoretical rate of sharing of IBD segments of length *y* for a given demographic history by λ(y), the observed rate of IBD sharing $\hat{\lambda }(y)$ can be expressed as


(17) $$\hat{\lambda }(y)=\text{FP}(y)+\int_{0}^{\infty }\lambda (z)\text{Recall}(z)R(y\;|\;z)dz,$$

where FP(y) denotes the false positive rate of IBD segments of length *y*, Recall(z) the power to detect a segment of length *z* and R(y|z) the probability that an IBD segment of true length *z* is detected with length *y*. See Supplementary Note 2 for how we approximated these three error rates using estimates from simulated IBD segment data.

In practice, we limit the integral to an interval around *y*:


(18) $$\hat{\lambda }(y)=\text{FP}(y)+\int_{y-a_{-}}^{y+a^{+}}\lambda (z)\text{Recall}(z)R(y\;|\;z)dz.$$

We chose $a_{-}=a^{+}=5$ in this study as we found that the inferred IBD length is almost always within 5 cM from its actual size (Supplementary Fig. 3b,c). We evaluated this integral by summing over discrete bins of width 0.25 cM.

### Simulating IBD segments

To simulate IBD segments, we used Msprime (Baumdicker *et al.* 2022) to simulate genome-wide genealogies and then extracted IBD segments longer than 2 cM using tskit’s Python API (Kelleher *et al.* 2024). Although we only use segments longer than 8 cM for inference in our simulated data, we include shorter segments to simulate length bias errors (see ‘Simulating IBD detection errors’). We simulated the 22 autosomes by stitching them together and used the HapMap (Gibbs *et al.* 2003) recombination map. We set the recombination rate between two consecutive chromosomes to log2, as recommended in Msprime’s documentation. We used the Wright–Fisher simulation engine of Msprime for the most recent 200 generations and then switched to the more efficient Hudson’s simulation engine. This hybrid simulation approach accurately captures IBD sharing (Nelson *et al.* 2020). We simulated IBD segments up to 2,500 generations backward in time, as deeper coalescence effectively does not contribute to IBD sharing longer than a few centimorgans.

### Simulating IBD detection errors

As explained in Section, we consider three types of IBD detection errors: false positives, limited recall, and length errors. To simulate false positives, we add segments to the ground-truth set. For example, if the false positive rate of IBD of length *l* is f(l), then we model the number of false positive segments whose length between *l* and l+Δl as following a Poisson distribution with mean f(l)Δl. We draw n∼Poisson(f(l)Δl) segments of length $l+\frac{\Delta l}{2}$ and add these false positives to the ground-truth set. In practice, we use Δl=0.25cM, and we set the false positive rate to be equal to the expected sharing rate of a population with $N_{e}=25,000$ (Fig. 4a). To simulate recall and length bias, we keep each segment in the ground-truth set with the probability of recall(l). For simulation purposes, we use the functional form $\text{recall}(l)=1-\frac{1}{1+0.025\sqrt{l}e^{0.25l}}$, which is adapted from Ralph and Coop (2013) (Fig. 4b). If kept, the segment’s new length is its original length plus ϵ∼N(0,1.5). If a segment’s length drops below the length cutoff of 8 cM (we use IBD segments longer than 8 cM for inference in our simulation), we discard it. We note that ϵ does not depend on *l* because we found length bias is similar across different segment lengths (Supplementary Fig. 3b). We depict an example of ground-truth IBD vs. ground-truth IBD with errors simulated as described above in Fig. 4c.

### Empirical data analysis

**Fig. 3.**
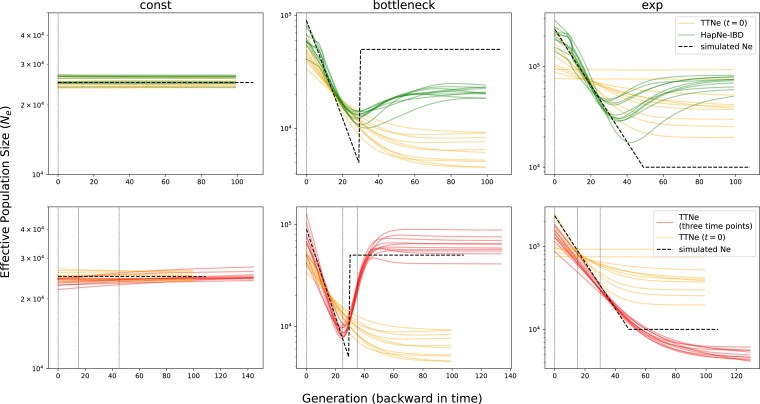
Performance of Ttne in various simulated demographic scenarios. Each column visualizes one of the three scenarios. The first row compares the results of Ttne and HapNe-IBD using contemporaneous samples at t=0. The second row visualizes the results of Ttne using contemporaneous samples at t=0 (in orange) or samples from three different time points (in red, sampling times indicated by vertical lines). All results shown here use n=30 samples at each time point for simulations with three sampling times or n=90 for simulations with only a single set of contemporaneous samples. We show 10 independently simulated replicates for each scenario. We depict the results of other sample sizes in Supplementary Figs. 24–28.

**Fig. 4.**
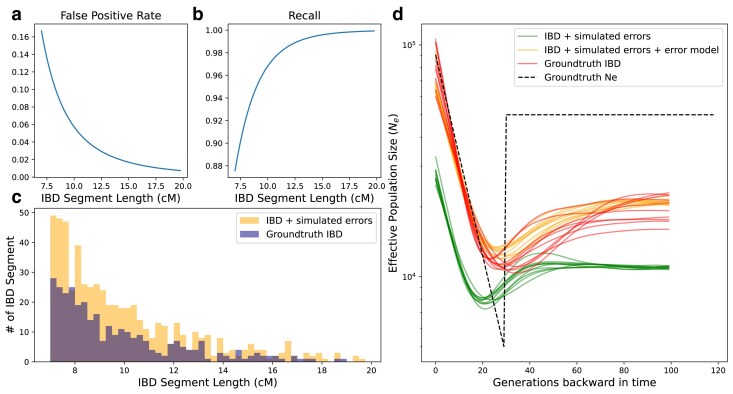
Explicit IBD error modeling alleviates biases from IBD detection errors. a) we simulated false positive IBD segments using the expected sharing rate of two random haplotypes drawn from a well-mixed population of effective size 25,000. As a result, in our simulated bottleneck scenario, the noise-to-signal ratio is roughly 1:1. b) Simulated recall. The recall function with respect to tract length *l* (in cM) is $1-\frac{1}{1+0.025\sqrt{l}e^{0.25l}}$. c) Histogram of ground-truth IBD vs. IBD with simulated detection errors. The histogram visualizes IBD distribution for 180 diploid individuals sampled contemporaneously at t=0 from the same bottleneck demography as in Fig. 3. Simulated false positive and power are as in (a,b), and the simulated length bias is drawn from a normal distribution with μ=0,σ=1.5 (so that 99.7% of the segments should be within 4.5 cM from their original length). d) Inferred $N_{e}$ using ground-truth IBD (red), ground-truth IBD with simulated errors but without error correction, and with error correction. Here 180 diploid individuals were sampled at t=0. We show 10 independent replicates for each scenario. In this simulation, the error model is specified as used in simulation.

**Fig. 5.**
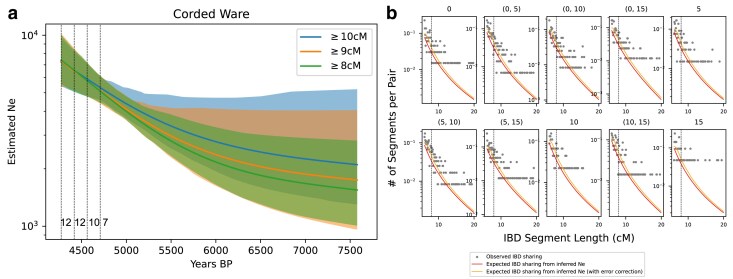
Inferred effective population size trajectory of Corded Ware individuals. a) We visualize the estimated effective population size trajectories with different IBD length cutoffs. The shaded areas depict the 95% C.I. The vertical dashed lines indicate the median age of samples in each of the four sampling clusters. The sample sizes at each sampling cluster are 12, 12, 10, and 7, respectively. We show the sample size in the lower left corner, next to the vertical line that indicates the sampling time for each set of samples. b) We visualize the fit between the empirically called IBD segments (gray dots) and the expected IBD sharing computed using the inferred $N_{e}$ trajectory (estimated with IBD length cutoff ≥8 cM, indicated by dashed vertical line). Detected IBD segments ≥ 6 cM are shown, but segments 6–8 cM are not used for inference. The red and orange lines depict the expected IBD sharing assuming no detection errors and with detection errors, respectively. Four sampling clusters result in 4+4*3/2=10 IBD subplots, including both IBD sharing within one cluster and across two clusters (as described in equation (7)). The sub-figure title indicates the sampling time (measured in generations backward in time starting from the most recent sampling cluster) associated with the sample set. For example, 0 indicates that this subplot shows IBD within the sample set sampled at t=0. (0,5) indicates that this subplot depicts IBD between two sample sets dating to t=0,t=5, respectively.

To apply Ttne to empirical aDNA time-transect, we compiled published data from two periods and regions. Throughout, we imputed ancient genomes one by one using GLIMPSE1 (Rubinacci *et al.* 2021) and the 1000 Genome haplotype reference panel (1000 Genomes Project Consortium 2015). We then ran Ancibd (Ringbauer *et al.* 2023) using its default setting. We selected a subset of unrelated individuals passing the coverage requirement for accurate IBD calling (>1x for 1,240 k data and >0.25x for WGS data, (Ringbauer *et al.* 2023), raw IBD output is available in Supplementary Tables 2 and 3). To filter ancestry outliers, we performed PCA using smartPCA. As widely done in ancient DNA analysis, we projected ancient genomes onto coordinates calculated from modern West Eurasian Human Origins samples using shrinkage correction (Lazaridis *et al.* 2014). Based on the median date of each individual’s radiocarbon or context date, we grouped samples by five generations (Supplementary Fig. 32), assuming a generation time of 29 years (Tremblay and Vézina 2000; Helgason *et al.* 2003; Moorjani *et al.* 2016). Grouping samples by time is necessary because runtime scales quadratically with the number of distinct sampling points. We found that this level of grouping has negligible effects on the estimated $N_{e}$ (Supplementary Note 3, Fig. 5). To estimate error models for IBD detection, we first computed the average coverage of 1,240 k and WGS data in the empirical samples (Supplementary Table 1) and then simulated IBD (described in detail in Supplementary Note 2, Figs. 3 and 4) by downsampling 1,240 k/WGS BAM files to the average coverage, and used the average of the estimated 1,240 k and WGS error models for the empirical data analysis. Given that our estimated $N_{e}$ has overall the same order of magnitude as $N_{e}$ estimated from LD using hapNe-LD (Fournier *et al.* 2023) (Supplementary Figs. 31 and 36) and that the inferred Ne trajectory is robust to modest misspecification of the error mode, we believe that our estimate is not substantially biased.

## Result

### Performance on ground truth IBD

We simulated IBD under three different demographic scenarios: first, a constant population at Ne=25,000; second, a constant population undergoing an instantaneous 10-fold bottleneck (from 50,000 to 5,000) 30 generations ago and then growing back exponentially to 100,000 at a steady rate; and, third, an exponentially increasing population at a constant rate starting from 50 generations ago (from 10,000 to 250,000).

As a baseline comparison, we first used contemporaneous samples (where all samples are assumed to be taken at t=0) and compared Ttne with HapNe-IBD. We found that both methods perform similarly in this common setting (first row in Fig. 3). At deeper time depth, HapNe-IBD estimated $N_{e}$ tends to converge to the average $N_{e}$ over time, while Ttne tends to converge to the estimated $N_{e}$ value from the more recent time. These different behaviors mainly stem from different choices of regularization.

Next, we simulated IBD sharing among three sampling points for each demographic scenario to evaluate the advantage of utilizing samples from multiple time points. For the constant demography, we sampled at t=0,15,45 generations ago. For the bottleneck demography, we took samples at t=0,25,35 generations ago (this sampling scheme has samples both before and after the bottleneck). For the exponential growth demography, we sampled at t=0,15,30 generations ago. As, to the best of our knowledge, no other IBD-based method can estimate $N_{e}$ trajectories from time-series sampling, we could not compare Ttne to others in such sampling regimes. Instead, we compared the results of time-series samples against those of contemporaneous samples. To make the comparison fair, if *N* samples were taken at the three sampling time points, then 3N samples were taken at t=0 for the respective contemporaneous sampling.

We found that time-series sampling can more accurately recover rapid changes in $N_{e}$ than contemporary sampling. In the simulated bottleneck demography, for example, the time-series sampling inferred changes of $N_{e}$ around the bottleneck more precisely than contemporaneous sampling (second row in Fig. 3). We also experimented with two sampling points postdating the bottleneck. Even without samples predating the bottleneck, this sampling more accurately recovered both the magnitude of the bottleneck and the large population size before the bottleneck (Supplementary Fig. 23), compared with the baseline with only contemporaneous samples. We note that when the sample size becomes too small, both Ttne and HapNe-IBD lose the power to recover the fluctuating $N_{e}$ history and output an effectively constant $N_{e}$ over time (Supplementary Figs. 24–28). This flattening in the low data regime is expected because regularization dominates the signal.

#### Performance on ground-truth IBD with simulated errors

We simulated IBD detection errors as described in (also see Fig. 4a,b,c). We then inferred Ne from the IBD calls with added errors, applying the correction formula described in. The results demonstrate that our correction model effectively removes biases resulting from IBD detection errors (Fig. 4d). We note that the error parameters are correctly specified in our simulations, while these parameters can only be estimated in practice. Therefore, we investigate the effects of misspecified error models in Supplementary Note 4. We simulated IBD detection errors at various coverages (Supplementary Figs. 6–9) and inferred $N_{e}$ using different error models. We found that severe misspecification mainly affects the absolute value of the inferred $N_{e}$ trajectory, and the misspecified model can still correctly recover qualitative dynamics of $N_{e}$ (e.g. whether the population size is growing or remains constant, see Supplementary Figs. 10–13,34,35).

### Applications to individuals associated with the Corded Ware culture

Individuals associated with the Corded Ware culture, hereafter referred to as “CW”, were early carriers of steppe-related ancestry that arrived in a major genetic turnover in a broad region across Central, Northern, and Eastern Europe in the third millennium BCE. CW individuals derive ca. ∼75% of their ancestry from a source similar to the Pontic-Caspian steppe pastoralists(Allentoft *et al.* 2015; Haak *et al.* 2015; Papac *et al.* 2021), and the remaining ∼25% of their ancestry from a source similar to the earlier European Copper Age farmers associated with Globular Amphora culture (GAC) (Ringbauer *et al.* 2023).

Because our demographic model assumes a single panmictic population, we screened for ancestry outliers using principal component analysis (PCA) (Supplementary Fig. 17). We removed nine individuals outside the main cluster of CW individuals (following a similar approach as in Mathieson and Terhorst 2022). We only kept the individual with higher coverage from pairs of closely related individuals, here defined as having at least three IBD segments longer than 12 cM or the summed length of all IBD segments longer than 12 cM exceeding a total of 100 cM. This filtering results in 42 individuals. We grouped individuals of similar dates by five generations, resulting in four groups with 12, 12, 10, and 7 individuals each (see Supplementary Fig. 32 and Fig. 5a). We excluded the remaining one individual from $N_{e}$ inference. We also explored different grouping (e.g. grouping by two or seven generations), which yielded qualitatively similar results (Supplementary Fig. 19).

Applying Ttne, we inferred a ∼3-fold population expansion in a ∼1,000 year period starting ∼5,500 BP (Fig. 5). This signal is consistent with archaeological evidence that the CW culture rapidly expanded into central, northern, and northeastern Europe from a currently unknown origin (Kristiansen *et al.* 2017). The inferred $N_{e}$ curve does not provide a conclusive date on the exact onset of this population growth, possibly due to the relatively small sample size so that the regularization scheme smooths the inferred $N_{e}$. We also note that because we can only infer comparably long IBD segments in imputed aDNA data, we have minimal power to infer population sizes at deeper time depths: Under this inferred CWC demography, 97.5% of the IBD segments used as signal originate from coalescence within the past 50 generations (Supplementary Fig. 18). Therefore, the estimated $N_{e}$ beyond 50 generations likely does not reflect the true $N_{e}$ but is the result of regularization.

Notably, the previously described recent admixture of CW individuals (Chintalapati *et al.* 2022) may bias LD-based methods to infer $N_{e}$ due to admixture LD. For instance, when applying HapNe-LD on the CW individuals, it reports substantial cross-chromosome LD and warns that the result may be substantially biased by recent admixture (Supplementary Fig. 31). The inferred $N_{e}$ either suggests a constant population when using early CWC individuals (dated to before 4,460 BP) or a recently declining population when using later CWC individuals (dated to after 4,460 BP). As noted in Fournier *et al.* (2023), admixture LD can lead to falsely inferred population contraction, explaining the mismatch to the result of Ttne based on IBD segments. To investigate whether this known admixture will affect IBD-based estimate, we performed simulations (described in detail in Supplementary Note 5) using realistic Corded Ware demography (Supplementary Fig. 14) and found that the IBD-based recent population size estimate is still accurate (Supplementary Fig. 15).

### Inferring population size trajectories in post-Anglo-Saxon Medieval Britain

The Anglo-Saxon migration in the 5–7th century CE constituted a significant gene flow into the British Isles (Gretzinger *et al.* 2022). We compiled ancient genomes from Southeastern Britain during the Anglo-Saxon migration period (Gretzinger *et al.* 2022), and individuals from Cambridgeshire (also Southeastern Britain) during the Medieval age around the time of the Black Death (1,346–1,353 CE) (Hui *et al.* 2024). A PCA analysis suggests that genetic diversity within individuals from Hui *et al.* (2024) is a subset of that of individuals from Gretzinger *et al.* (2022) (Supplementary Fig. 33), which is expected given that ancestry clines will contract following a gene flow period. For data from Hui *et al.* (2024), we used individuals from sites labeled as “later medieval” and excluded sites labeled as “post-medieval” (1,550–1,855 CE), of which 69 samples remained after coverage filtering. The “later medieval” sites date from 940 to 1,561 CE, spanning approximately 20 generations. Because we found this level of sample time heterogeneity leads to only a moderate upward bias in estimated $N_{e}$ (Supplementary Note 3) and because only a few samples are radiocarbon-dated, which prevents a more fine-grained time stratification, we group all samples from Hui *et al.* (2024) and apply the time heterogeneity correction formula described in Supplementary Note 3. We use the median age of these samples (1,239 CE) and set the time radius to be 10 generations in both directions. For the rest of the samples, we grouped them by five generations as described in Section ‘Empirical data analysis’. This grouping results in four sets of samples of size 69, 20, 76, 35.

Applying Ttne, we inferred a continuously growing population from the early Middle Ages to the onset of the Black Death in England in the 14th century. The inferred Medieval population growth is consistent with historical demographic estimates (Cipolla 1972). Agriculture expanded, and advancements in medieval technology allowed more land to be farmed. Population growth only stopped with the 14th century Crisis of the Late Middle Ages (including famines and the Black Death) when population counts fell abruptly (Cipolla 1972). Given that our latest samples are from the 1,500s, this demographic decline is not yet observable.

## Discussion

We have introduced Ttne, a tool for estimating population size trajectory from IBD segments in time-series ancient DNA data. We show using simulations that our method’s ability to utilize IBD sharing signals among multiple time points recovers population size changes more accurately than previous methods that assume that all samples are contemporaneous or fit an average $N_{e}$ curve for time heterogeneous samples (e.g. HapNe-IBD). IBD-based methods have a unique advantage because IBD-sharing signals originate exclusively in the recent past (usually within 50–100 generations, or equivalently, ∼ 3,000 years). The shallow time depth of IBD sharing shields an IBD-based signal from confounding by deep population history and admixtures common in the human past that would bias allele-frequency or LD-based methods. Moreover, IBD-based methods is robust to various technical biases, as calling IBD works for various sets of ascertained variants and can tolerate substantial genotyping errors as is typical in ancient DNA data (Ringbauer *et al.* 2023).

A limitation of our method is its assumption of a single, well-mixed population. Violations of this model complicate interpreting inferred changes in $N_{e}$. As effective population size as estimated by our method is the inverse of the coalescent rate, any factor that affects the coalescent rate also affects estimated $N_{e}$ trajectory, including effects from migration, selection (Schrider *et al.* 2016; Cousins *et al.* 2024), admixture (Supplementary Note 5), and population structure (Mazet *et al.* 2015). While this model of a panmictic population allows for a helpful summary quantity $N_{e}$ that is widely relevant in many population genetic theories, we recommend always considering such factors when interpreting $N_{e}$ as population size.

Regularization is a challenging but necessary technique in many inverse problems for which various approaches have been developed. Prior work to infer effective population size trajectories regularize by restricting the solutions to specific functions, such as piecewise constant growth rates (e.g. Browning and Browning 2015; Fournier *et al.* 2023). Terhorst (2024) uses a Bayesian approach to sample from the posterior of population size histories and obtain point estimates by averaging over posterior samples. In this work, we use a penalized maximum-likelihood estimation based on a combination of the $\ell_{2}$ norm of the first and second derivatives, similar to Ralph and Coop (2013) and DeWitt *et al.* (2021). Our specific hyperparameter selection scheme maximizes regularization strength until the regularized function causes a significant drop in goodness of fit to the observed data. This design avoids over-fitting in applications with little data by adequately increasing regularization strength. Despite this automatic choice, there is no theoretical guarantee that this hyperparameter selection will avoid over- or underfitting. We recommend checking model fitting (for example Supplementary Fig. 2) and hope that future works will systematically explore various regularization approaches against simulated data and compare their relative advantages and weaknesses.

To apply Ttne to empirical aDNA studies, the temporal distribution of samples has important implications for inference. While sampling is often limited by aDNA preservation and availability of human remains, our simulations can guide selecting samples in empirical studies targeting specific demographic events. Unsurprisingly, sampling closer to the demographic event of interest increases resolution. In Supplementary Fig. 23, for example, we found that having an additional set of samples aside from samples at t=0 improves the inference of the strength and timing of a bottleneck.

**Fig. 6.**
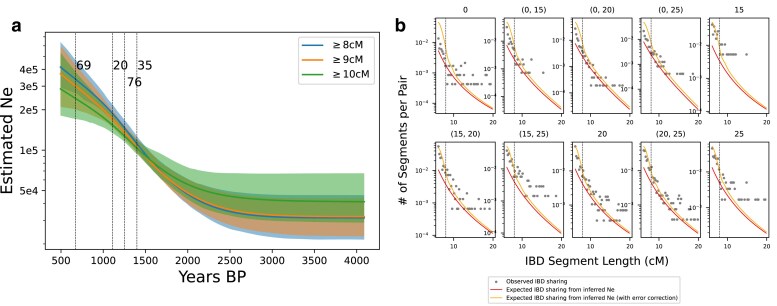
Inferred effective population size from British genomes. a) Inferred $N_{e}$ using different minimum IBD length cutoffs. We indicate 95% C.I. computed from bootstraps as shaded areas. We note that the inferred $N_{e}$ starts 10 generations more recent than the median age of the youngest set of samples because of the time-heterogeneity correction described in the main text. b) Similar to Fig. 5, we visualize the fit between empirically called IBD segments (≥6 cM) and the model predicted IBD using the $N_{e}$ estimated using all segments ≥8 cM (indicated by vertical dashed black lines). The IBD segments between 6 and 8 cM are not used during inference but are visualized here for comparison.

We applied Ttne to two empirical datasets: individuals associated with the Corded Ware culture ca. 5,000 years ago and individuals from medieval Britain (6th–14th century). In both cases, we found evidence of recent population growth. Notably, the inferred effective population size of the British Isles is an order of magnitude larger than that of Corded Ware, demonstrating IBD sharing is a valuable signal even in large populations given sufficient sample size (Fig. 6). One caveat of our analysis of the British dataset is that only a relatively small fraction of individuals’ birth dates in Hui *et al.* (2024) can be unambiguously dated to pre- or post-black death (ca. 1,350 CE). Consequently, we had to group all later medieval samples. To accurately estimate the regional demographic impact of black death, further studies could aim to generate data from dated individuals to construct a time-series dataset spanning the entire black death period.

As in-solution capture kits similar to the 1,240 k capture widely used in aDNA studies have become widely available (Rohland *et al.* 2022; Davidson *et al.* 2024) and whole-genome sequencing is becoming more cost-effective, we anticipate that more studies will be able to generate genome-wide ancient DNA data at increasingly large scales. As the published aDNA record keeps growing rapidly (Mallick *et al.* 2024), more studies can utilize time-transect to study fine-scaled demographic changes in specific regions over time. Therefore, Ttne will also become relevant for currently less well-studied regions, helping to reveal past population size trajectories around the globe, particularly for periods where little direct historical evidence on past population sizes is available.

## Supplementary Material

iyae212_Supplementary_Data

iyae212_Peer_Review_History

## Data Availability

No new DNA data were generated for this study. The BAM files of individuals associated with the Corded Ware culture used in this study were downloaded from ENA repositories associated with their respective publications (Supplementary Table 4). The BAM files of individuals from Britain originate from Gretzinger *et al.* (2022) (ENA accession number PRJEB54899) and Hui *et al.* (2024) (ENA accession number PRJEB59976). The development code of Ttne is deposited and maintained as a GitHub repository at https://github.com/hyl317/IBDTimeSeries. Ttne is available as a python package (https://pypi.org/project/TTNe/) and can be installed via pip. Supplemental material available at GENETICS online.
