# Supplementary material for: Estimating effective population size trajectories from time-series identity-by-descent segments
Source: Genetics. 2025 Jan 24;229(3):iyae212. doi: 10.1093/genetics/iyae212 (PMC11912830; doi:10.1093/genetics/iyae212)
Supplement: iyae212_Supplementary_Data [file iyae212_supplementary_data.zip › Supplemental_Material_GENETICS-2024-307622.pdf]

Supplementary Materials

Yilei Huang[1,2,†], Shai Carmi[3] and Harald Ringbauer[1,†]

[1]Department of Archaeogenetics, Max Planck Institute for Evolutionary Anthropology, Leipzig, Germany
[2]Bioinformatics Group, Institute of Computer Science, Universität Leipzig, Leipzig, Germany
[3]Braun School of Public Health and Community Medicine, Hebrew University of Jerusalem, Jerusalem 9112102, Israel
[†]Corresponding author – Email: yilei_huang@eva.mpg.de, harald_ringbauer@eva.mpg.de

December 17, 2024

Supp. Note S1 Choosing regularization parameters

Our regularization has two components: the first is related to the second derivative of $\mathbf{N_e}$, and the second to the first derivative (Eq.8 and Eq.9 in main). The two components have weight α,β respectively. The second term is intended to stabilize the estimated $\mathbf{N_e}$ at deeper time ranges with little coalescence from long IBD segments. As stated in the main text, we chose to fix $\beta = 250$ throughout.

In contrast, choosing a single α that works across a wide range of demographics and sampling strategies was challenging. Therefore, we used 5-fold cross-validation to select the appropriate value for α. We equally divide all pairs of individuals into five parts. We hold out one part for validation and use the remaining four parts of pairs to estimate $\mathbf{N_e}$ at a given α. We note that pairs of individuals are not independent (as individuals are shared among pairs), violating the assumption for cross-validation. Therefore, we experimented with partitioning by chromosomes or individuals. However, we found that in both cases, the small sample sizes lead to noisy estimates.

We use the inferred $\mathbf{N_e}$ to calculate the so-called deviance statistics on the held-out validation set. The deviance statistics is a commonly used goodness-of-fit measure in Poisson regression. It is defined as follows,

$$D = 2\sum_{i=1}^{n}\left(y_i \log\left(\frac{y_i}{\lambda_i}\right) - (y_i - \lambda_i)\right),$$

where the summation is over small length bins, λ_i denotes the number of IBD segments within that length bin predicted from the inferred $\mathbf{N_e}$, and y_i the number of observed IBD segments falling within that length bin. The deviance statistic quantifies how much the observed counts deviate from those predicted by the fitted model. It equals zero exactly when $y_i = \lambda_i \forall i$. When $y_i = 0$, we set the individual summand, otherwise undefined due to the log, to 0 because $\lim_{x \to 0^+} x \log x = 0$.

We then use the average value of deviance statistics over the five validation sets, denoted by $D(\alpha)$, as the metric to select α. We perform a grid search for α from 50 to 1e7, using 30 values evenly spaced on a log scale. Usually, one would choose the α that yields the smallest $D(\alpha)$. However, rapid oscillations of $\mathbf{N_e}$ will not substantially worsen the fit, even though that makes the $\mathbf{N_e}$ highly irregular. Based on this heuristic rationale, we aim to choose the strongest regularization (i.e., the smoothest predicted $\mathbf{N_e}$) that does not yield a substantially worse fit: We chose the biggest α so that $D(\alpha)$ remains within one plus the smallest $D(\alpha)$. Formally, the regularization α^* is then determined by

$$\alpha^* = \max\{\alpha : D(\alpha) \leqslant 1 + \min_{\alpha} D(\alpha)\}$$

We observed that all other things being equal, a dataset with a bigger sample size tends to have a smaller α^*, hence weaker regularization (Fig.S1). Heuristically, this is a desirable feature as more data contains more signal to infer sharp changes in $\mathbf{N_e}$, and the signal should increasingly overwhelm regularization. However, this automatic hyperparameter selection scheme based on an intuitive heuristic is not foolproof. We always recommend examining how the estimated $\mathbf{N_e}$ fits the observed IBD segment distribution to explore whether over-regularization occurs (see Fig.S2 for one example).

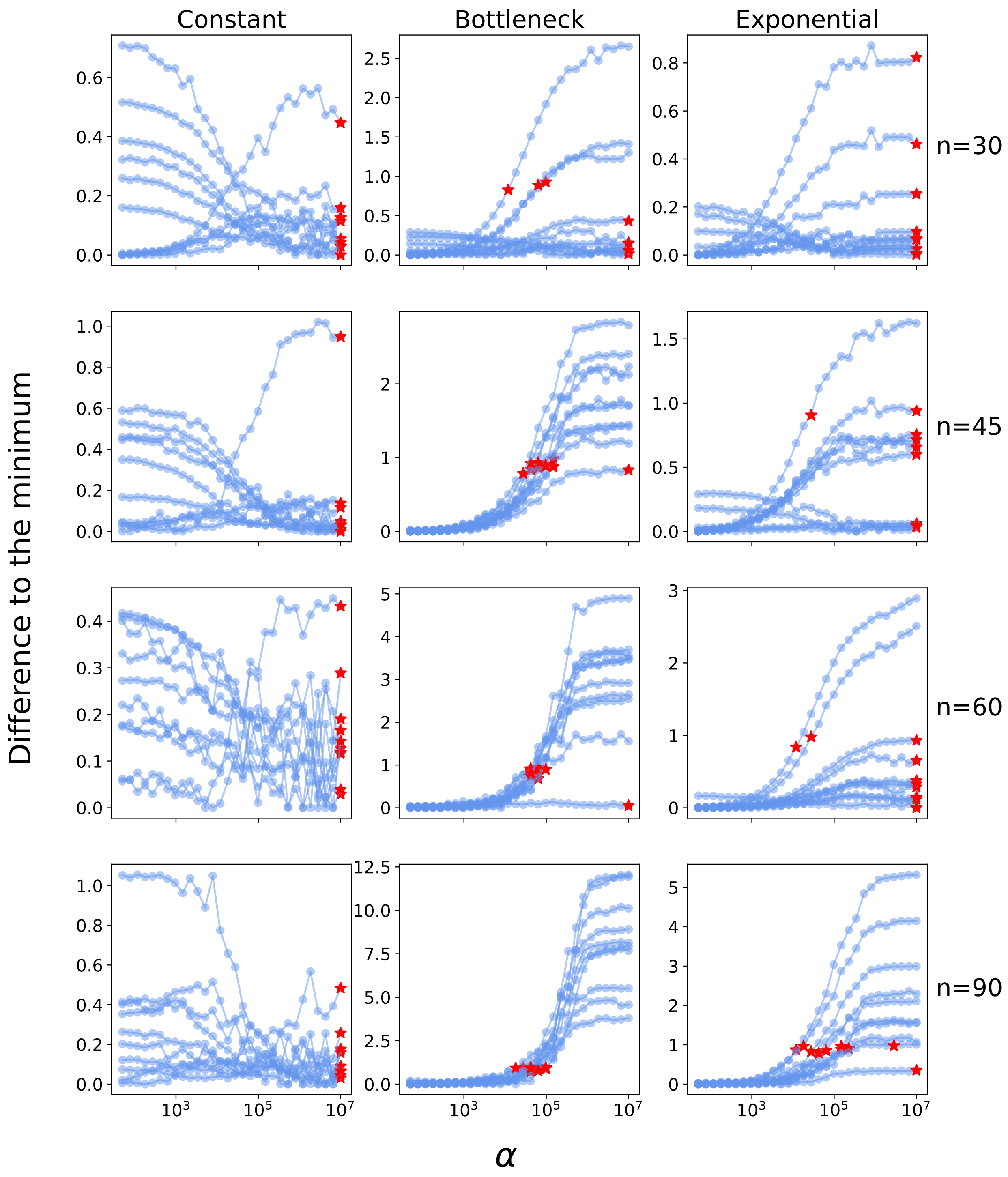

Figure S1: Effects of α on deviance statistics. We visualize deviance statistics $D(\alpha)$ for varying α values (i.e. strength of regularization). The y-axis is the difference to the minimum deviance statistics (over the search space of α). We show the results using a single sampling point at $t=0$ for various sample sizes (rows). Ten independent simulated replicates are depicted for each scenario (blue curves). We indicate α*, the "optimal" α we chose for each replicate (red star). We observed that models with multiple sampling time points have qualitatively similar behaviors (results not shown).

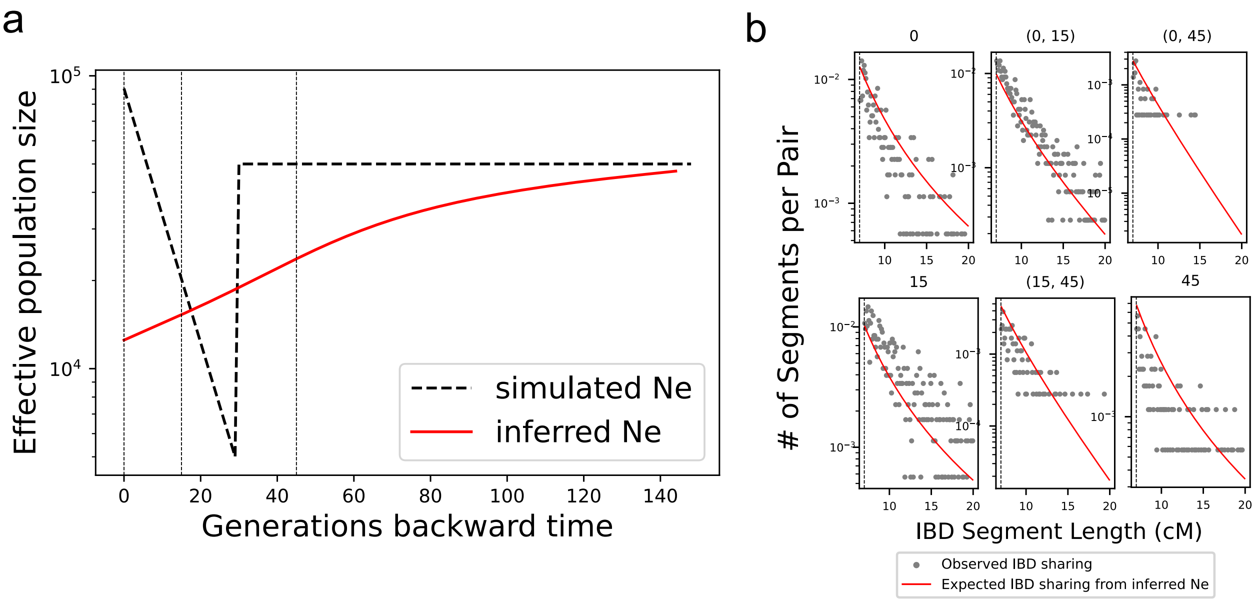

Figure S2: An example of over-regularization. We depict an example of over-regularized estimates of the bottleneck demography (when manually fixing $\alpha = 10{,}000{,}000$). **a** Inferred $\mathbf{N_e}$ (red) vs. simulated $\mathbf{N_e}$ (dashed black line).The bottleneck occurs at $t = 30$, and the three sampling times are $t = 0, 15, 45$ (vertical dashed black lines). **b** Comparison of empirical IBD to that from model fit. The sub-figure title indicates the sampling time (in generations). For example, 0 indicates that this subplot shows the empirical (gray dots) and predicted (red line) IBD within the first sample set sampled at $t = 0$. $(0, 15)$ indicates that this subplot depicts the empirical and predicted IBD between the first and second sample set, sampled at $t = 0, t = 15$, respectively. We note that the IBD sharing expected from the estimated $\mathbf{N_e}$ does not fit well with the empirical IBD within the $t = 15$ samples and across the $t = 0, t = 15$ and $t = 15, t = 45$ pairs. Because the model is over-regularized, the estimated N_e around the bottleneck period gets over-smoothed, and the model cannot recapitulate the large population size before the bottleneck. The former leads to underestimated IBD sharing between the $t = 0$ and $t = 15$ sample clusters, and the latter leads to overestimated IBD sharing between $t = 15$ and $t = 45$ sample clusters.

Supp. Note S2 Estimating IBD calling Error Parameters for Empirical aDNA Data

We consider three types of IBD detection errors (as described in the main text): false positives, imperfect power, and length bias. The following subsections describe how we estimated these three error types from simulations.

Supp. Note S2.1 Estimating False Positive Rates

We empirically estimated the false positive rate for the average coverage of samples. The estimation procedure is the same as outlined in Supplementary Note 7 in Ringbauer et al. [2023]. Briefly, eleven samples (Supplementary Table 1) with no IBD were selected and downsampled to a target coverage (in this case, the average coverage of samples used in our empirical data analysis). All eleven samples have both high-coverage WGS and 1240k data publicly available. To estimate false positive rates for a specific data type (1240k or WGS data), we downsampled the corresponding BAM files. We then estimated the false positive rates at various IBD lengths from downsampled data using 100 independently downsampled replicates. For each replicate, we counted the number of inferred IBD segments (as the eleven samples do not share IBD, any detected IBD is by definition false positive) in each length bin and divided by the number of pairs (in this case $\frac{11*10}{2}=55$) to get the false positive rate per pair at each length bin. We then averaged this false positive rate across the 100 replicates.

Supp. Note S2.2 Estimating IBD Length Bias

We define IBD length bias as the difference between the inferred and actual IBD length. To correctly estimate this bias, we need simulations where the precise boundary of ground truth IBD is known, and the data should be qualitatively similar to empirical aDNA data. Toward this end, we started with empirical aDNA BAM files on chromosome 3 and copied in IBD segments at known locations. We first subset reads by genomic regions they aligned to and then merge reads from different genomic regions and individuals to form synthetic pairs of individuals with IBD shared at defined genomic positions. In particular, we utilize the fact that parent-offspring pairs naturally share IBD along their whole genome. Therefore, to simulate a pair of individuals with one IBD of length lcM, we first randomly select a lcM along the chromosome. The first simulated individual takes reads aligned to the selected region from the BAM file of the parent, and the second individual

from the BAM file of the child. For the rest of the genomic regions, the two simulated individuals take reads from the BAM files of two genomes without IBD. We illustrate this approach graphically in Fig.S3a.

To simulate IBD sharing in WGS-like data, we used the WGS BAM files of a high coverage father-son pair (I3950, I3949) published in Wohns et al. [2022] for the IBD region. This father-son pair is associated with Afanasievo culture and was dated to 2879-2632 calBCE and 2844-2496 calBCE, respectively. To simulate IBD sharing in 1240k-like data, we used the 1240k BAM files of a mother-son pair (GRG080, GRG041) published in Rivollat et al. [2023]. The mother-son pair originates from a Neolithic burial site (Gurgy 'les Noisats') in present-day France from 4850–4500 BC. For the non-IBD region, we used I3255 (2139-1947calBCE, England_Bellbeaker, [Olalde et al., 2018]) and I2105 (3300-2800BCE, Ukraine_EBA_Yamnaya, [Mathieson et al., 2018]) for each of the two individuals in a simulated pair. No IBD sharing > 5cM is detectable in this pair - as the two individuals are separated by ca. 1000 years. The I3255 and I2105 were initially published with 1240k-capture aDNA data and were later also whole genome sequenced to high-coverage (data publicly available at `https://reich.hms.harvard.edu/ancient-genome-diversity-project`). We used the respective data types for our WGS and 1240k simulations.

For the Corded ware samples used in our empirical analysis (we performed analogous simulations for the UK data), the average coverage for 1240k data is 1.65x (calculated on the 1240k SNP set), and that for WGS data is 1.1x. Therefore, we simulated IBD of 8cM, 12cM, 16cM, and 20cM with coverage and data type corresponding to the empirical data (Fig.S3b). To calculate length bias, for each simulated groundtruth IBD, we recorded inferred IBD segments that cover at least half of the groundtruth segment (therefore, there can only be one unique inferred segment satisfying this condition, if it exists at all). The length bias is the length difference between the inferred and the groundtruth segments. If no such inferred segment exists, then this ground truth segment is considered not inferred, and no length bias is recorded.

We found that the length bias is asymmetrical (Fig.S3b,c). Most segments are inferred to be within 2cM longer than their groundtruth lengths. However, a much smaller albeit non-negligible proportion of segments are broken apart due to errors in the imputed genotypes. In addition, we found that length bias is similar across different IBD lengths (Fig.S3b). Therefore, we modeled length bias across different groundtruth IBD lengths with the same model for simplicity. We also found that length bias is overall smaller for 1.1x WGS data compared to 1.65x 1240k data (Fig.S3c), consistent with the results of Ringbauer et al. [2023], which finds that in terms of IBD calling performance WGS data is similar to 1240k data with approximately 3x more coverage. For simplicity, we estimated

a single-length bias distribution using the combined simulated WGS and 1240k data. We simulated 12cM IBD with 500 independent replicates for each 1240k and WGS data. Then, we fitted a single density estimation (KDE) with Gaussian kernels to estimate the length bias distribution (shown by red line in Fig.S3c).

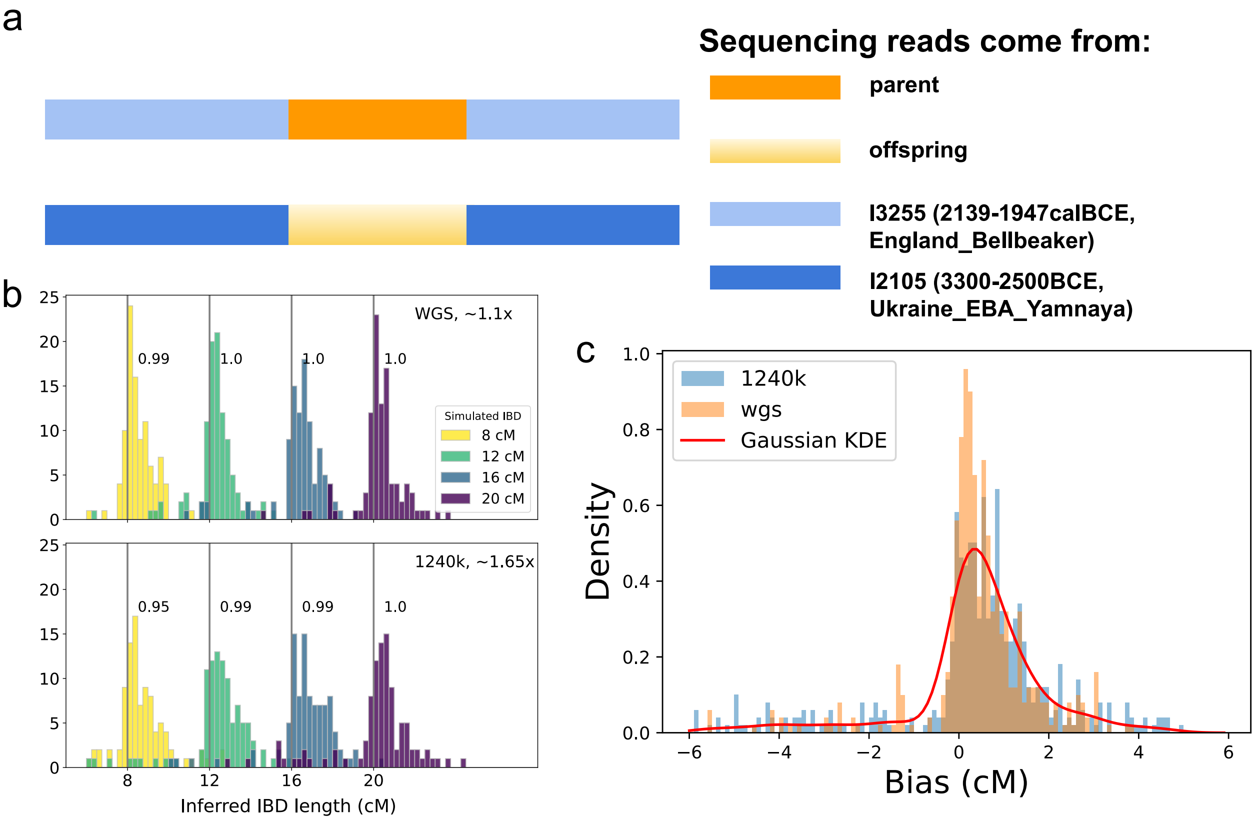

Figure S3: Estimating IBD length bias from simulated data. **a** A schematic showing our procedure of mixing sequencing reads aligned to different genomic regions to simulate IBD. **b** Histogram of inferred segment lengths on simulated IBD with 1.1x WGS data and 1.65x 1240k data. For each data type and IBD length, we simulated 100 independent replicates and then plotted the histogram of inferred IBD length. The number next to the vertical line indicates the recall for the corresponding segment length class. **c** Distribution of length bias of simulated 12cM groundtruth IBD for 1240k (shown in blue) and WGS data (shown in orange). We simulated 400 independent replicates in addition to that shown in **b** to fit the KDE. A red line shows the fitted Gaussian KDE.

Supp. Note S2.3 Estimating Recall

We use the same simulation scheme explained in the previous section (Supp. Note Supp. Note S2.2) to estimate recall. We simulated groundtruth IBD of 2cM,3cM,4cM,5cM,6cM, 7cM,8cM,9cM,10cM,11cM,12cM,16cM,20cM. Similar to Supp. Note S2.2, a groundtruth IBD segment is considered to be inferred if an inferred segment covers at least half of the true IBD segment. Because in calling IBD in empirical aDNA data we used length threshold of 6cM, here we apply the same length threshold of 6cM, regardless of the groundtruth segment length. Consequently, the recall for true segments less than 6cM is very low. We

found that the recall for 1.65x 1240k data is similar to that of 1.1x WGS data (Fig.S4). In the error model for our empirical data analysis, we use the average of the two. We linearly interpolated recall for segment lengths between the simulated IBD lengths.

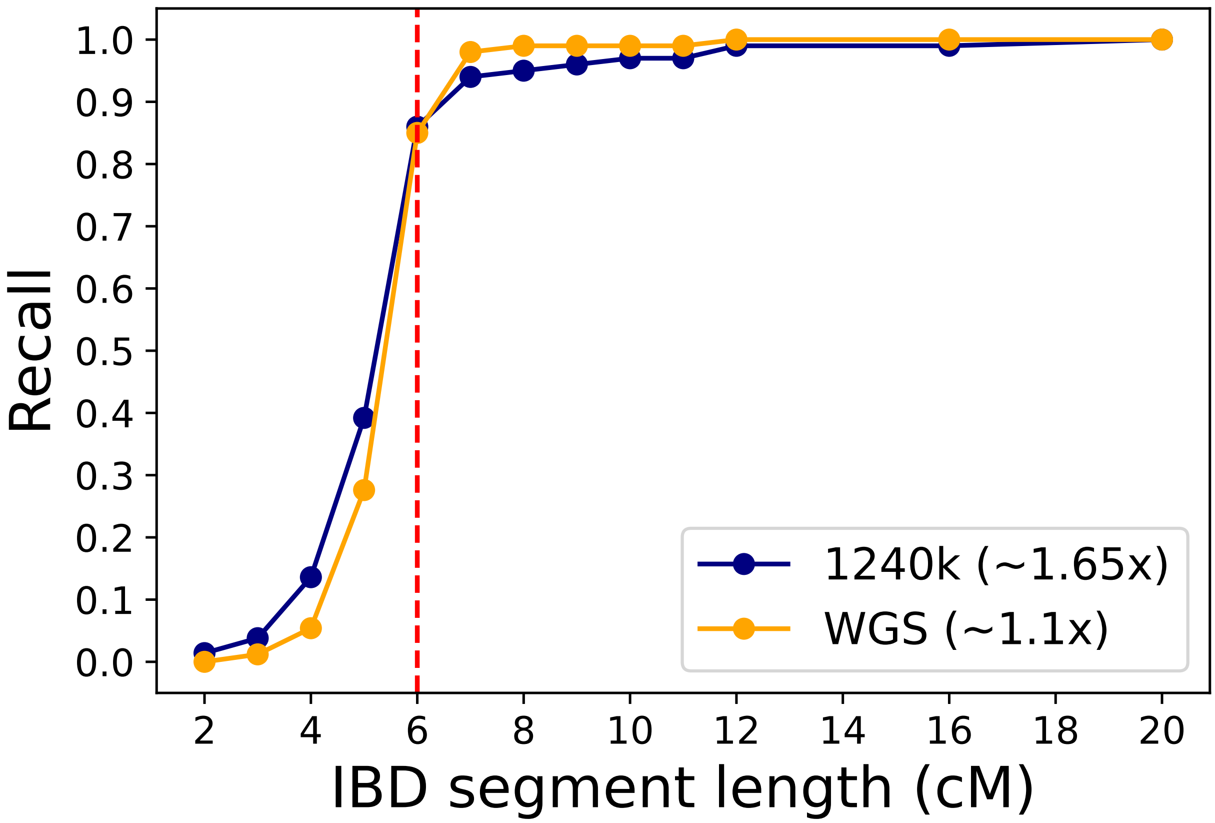

Figure S4: Recall as a function of segment length. We simulated groundtruth IBD of various lengths (see Fig.S3) and recorded ANCIBD's recall for 1240k (in navy) and WGS (in orange) data. We simulated 500 independent replicates for groundtruth segment less than 6cM and 100 replicates for the longer length classes.

Supp. Note S3 Sampling Time Uncertainty

Unlike contemporary DNA samples, aDNA samples typically do not have an exact date. Instead, they are dated either by radiocarbon dating or archaeological context, giving a plausible sample time range. This time interval can be broad, such as during the Hallstatt plateau that spans 400 years [Stuiver and Pearson, 1986]. Therefore, we describe how we can incorporate such sampling time uncertainty into our model and investigate how time uncertainty biases our estimates.

We assume all samples within a sample set S_i have the same time range. This may not be entirely accurate in practice; however, we consider this a reasonable modeling assumption to keep the model tractable while accommodating some of the complexities of empirical data. Each sampling time point t_i is associated with a radius r_i such that all samples in S_i are assumed to be uniformly sampled from the time range $[t_i - r_i, t_i + r_i]$. Our model and implementation do not require the interval to be centered around t_i, but we assume the time interval is symmetrical around t_i for notational simplicity. In practice, the radius r_i could be determined from 95.4% CI of calibrated radiocarbon age. Consider two sampling clusters S_1, S_2, each with time range $[t_1 - r_1, t_1 + r_1]$ and $[t_2 - r_2, t_2 + r_2]$. Denote the likelihood of observed IBD segments between S_1, S_2 with fixed sampling time $t_1^{'}, t_2^{'}$ as $\mathcal{L}(S_1, S_2 | \mathcal{N}, t_1^{'}, t_2^{'})$, which can be calculated as described in Methods. We can then integrate the likelihood over the time range as follows,

$$\begin{aligned} \mathcal{L}(S_1, S_2|\mathcal{N}) &= \int_{t_1 - r_1}^{t_1 + r_1} \int_{t_2 - r_2}^{t_2 + r_2} \mathcal{L}(S_1, S_2|\mathcal{N}, t_1^{'}, t_2^{'}) P(t_1^{'}, t_2^{'}) dt_1^{'} dt_2^{'} \\ &= \frac{1}{4 r_1 r_2} \sum_{t_1^{'} = t_1 - r_1}^{t_1 + r_1} \sum_{t_2^{'} = t_2 - r_2}^{t_2 + r_2} \mathcal{L}(S_1, S_2|\mathcal{N}, t_1^{'}, t_2^{'}) \end{aligned} \tag{1}$$

To explore the effect of having samples originating from different time points but modeled as contemporaneous, we simulated the constant and bottleneck model with samples taken uniformly from a time interval (specified by radius r) centered at specified time points. For example, for radius r and time point t, samples are uniformly taken from the time interval from $t - r$ to $t + r$. We found that when r is large (e.g., $r = 10$), not accounting for time heterogeneity leads to overestimates of N_e. Although the effect is moderate, this bias aligns with the findings from Fournier et al. [2023]. The correction procedure described in Eq.1 alleviates this upward bias (Fig.S5). However, as expected, this correction procedure runs $O(r^2)$ times slower because of the double summation in Eq.1.

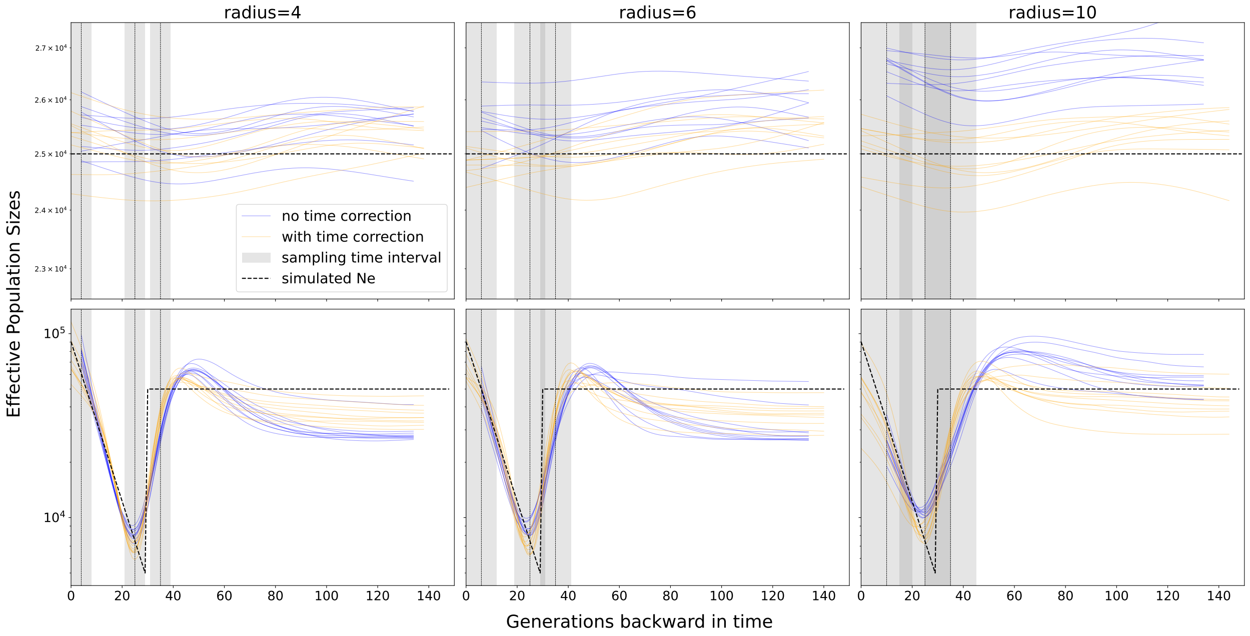

Figure S5: Simulated constant and bottleneck demography with samples drawn from intervals of various widths This plot shows the inferred $\mathbf{N_e}$ of simulated constant/bottleneck demography with samples uniformly drawn from intervals of width 8,12,20 generations (with and without the time heterogeneity correction in Eq.1). The vertical dashed lines indicate the mean sample ages for each of the three sampling points, and the grey-shaded area represents the time interval over which samples are drawn. The inferred $\mathbf{N_e}$ without Eq.1 correction does not start at $t = 0$ because the model assumes that the first sampling point is at the mean age of the most recent set of samples.

Supp. Note S4 Misspecification of IBD Error Models

In Fig. 4 we showed that when the error model is correctly specified, the effective population size trajectory $\mathbf{N_e}$ is inferred without bias. However, in practice, the error model is unknown and can only be estimated (e.g., via simulation or downsampling experiments). Therefore, we investigate whether our model can tolerate certain levels of error model misspecification without substantially impacting its performance.

We simulated groundtruth IBD using WGS and 1240k BAM files as described in Supp. Note S2.2. We downsampled WGS data to 2x, 1x, 0.5x, 0.25x, and 1240k data to 2x, 1.75x, 1.5x, 1.25x coverage. We estimated length bias and recall for the abovementioned scenarios following the same procedure as in Supp. Note S2.2 and Supp. Note S2.3. In addition, we estimated false positive rates following the same procedure as in Supp. Note S2.1. We visualized the estimated false positive rate (Fig.S6), recall (Fig.S7) and length bias (Fig.S8, S9). Using these estimated length bias, recall, and false positive model, we simulated IBD detection errors on the groundtruth IBD from the bottleneck demography (as in Section 1.7), and then inferred $\mathbf{N_e}$ applying each of the four estimated error models. Together with groundtruth IBD (i.e., no error), this experiment yields $5\times5=25$ combinations.

As expected, if IBD detection error exists but no error correction is applied, our model tends to underestimate N_e, plausibly driven by false positive IBD segments. This is most pronounced in 1240k data (for example, the first column of Fig.S12 and Fig.S13) and to a lesser extent in WGS data (first column of Fig.S10 and Fig.S11), plausibly because in the tested coverage range WGS data has far fewer false positives (Fig.S6). Conversely, if one over-corrects IBD detection errors, our model tends to overestimate N_e (see the first row of the abovementioned figures). Overall, we found that our method can tolerate reasonable levels of error model misspecification, as in most of the subpanels of Fig.S10, Fig.S11, Fig.S12, Fig.S13, the inferred $\mathbf{N_e}$ remains largely unbiased.

Importantly, the effect of misspecified error models on the inferred magnitude of $\mathbf{N_e}$ depends on the relative order of magnitude between the false positive rate and true IBD sharing rate. When the false positive rate is large relative to the actual IBD sharing rate, which usually happens for populations with large $\mathbf{N_e}$, model misspecification will cause substantial bias in the magnitude of inferred $\mathbf{N_e}$, particularly for 1240k data (Fig.S34, Fig.S35); conversely, when the false positive rate is small relative to the IBD sharing rate, model misspecification only has marginal impact on inferred $\mathbf{N_e}$ (Fig.S11, Fig.S13). Eq.5 in the main text shows that the amount of IBD sharing approximately scales with the inverse of N_e. Therefore, if the inferred number of IBD segments is inflated by a factor of p (either due to false positives or other reasons), the inferred N_e will drop to a fraction $1/p$

of the true value.

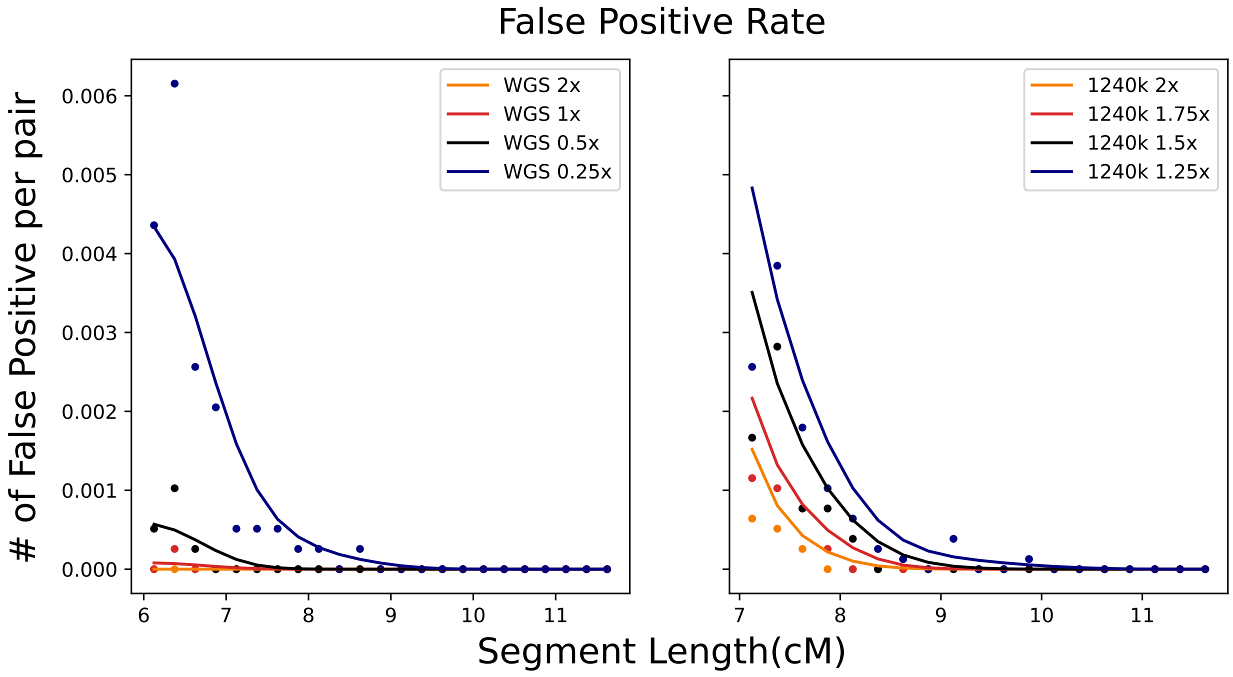

Figure S6: Estimated false positive rate at various coverages for WGS and 1240k data. The y-axis depicts the average number of false positive IBD segments on chromosome 3 within a length bin of 0.25cM for a pair of diploid individuals. The dots depict empirical values obtained from simulated data, and lines are numerically fitted to the dots.Left: WGS data; right: 1240k data. Note that in the two subfigures, the x-axes have different ranges.

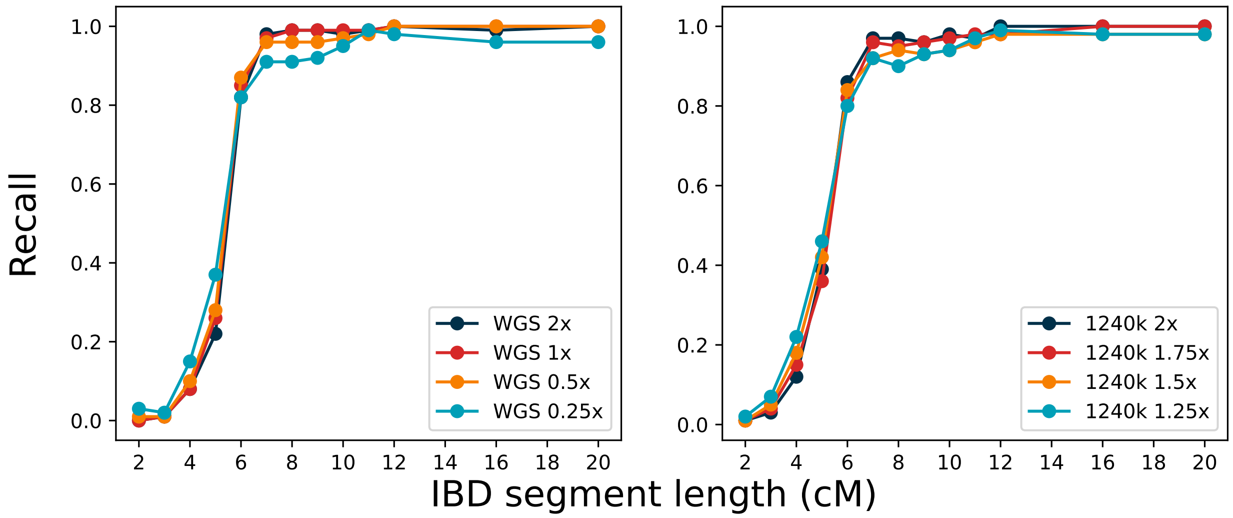

Figure S7: Estimated recall at various coverages for WGS and 1240k data. The Y-axis depicts the recall. Left: WGS data; right: 1240k data. We estimated Recall on 100 simulated IBD segments.

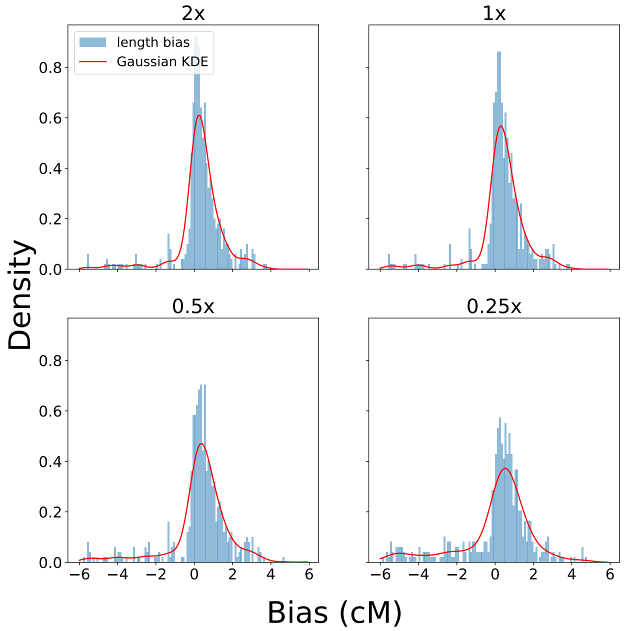

Figure S8: Length bias of inferred IBD segments for WGS data at various coverage The y-axis of the histogram (light blue bars) represents density. All subpanels share the same x and y-axis range. The red lines depict the fitted Gaussian KDE.

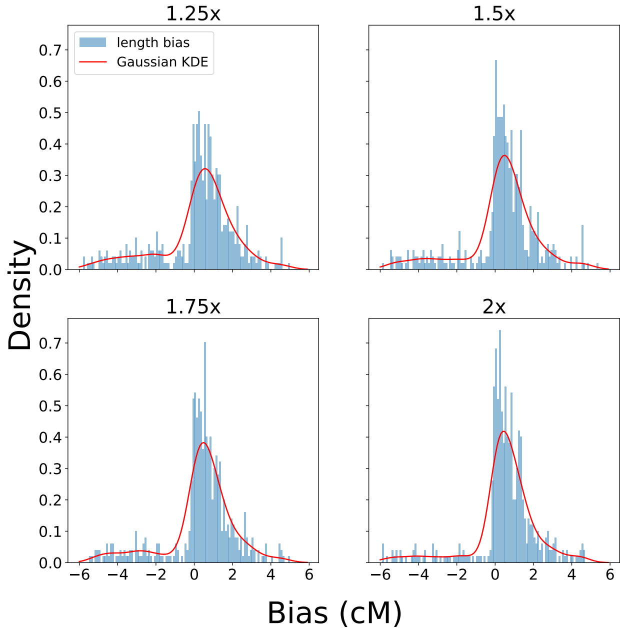

Figure S9: Length bias of inferred IBD segments for 1240k data at various coverage Same as Figure S8 but simulated with 1240k data.

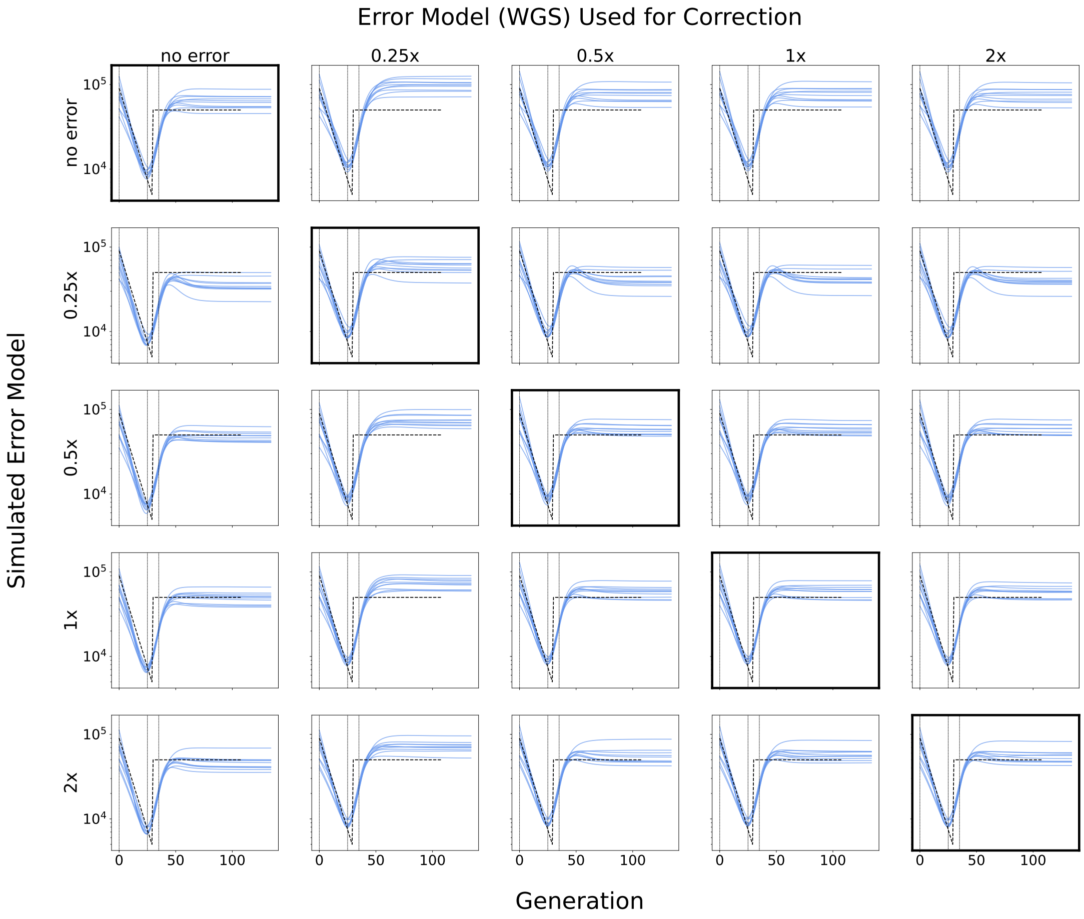

Figure S10: Inferred N_e of a bottleneck demography with various simulated WGS-like error models Vertical dashed lines represent the sampling time point. This is the same bottleneck demography as in main Fig.3. All results in the same row have the same simulated error model, while all results in the same column have the same error model used for correction during inference.

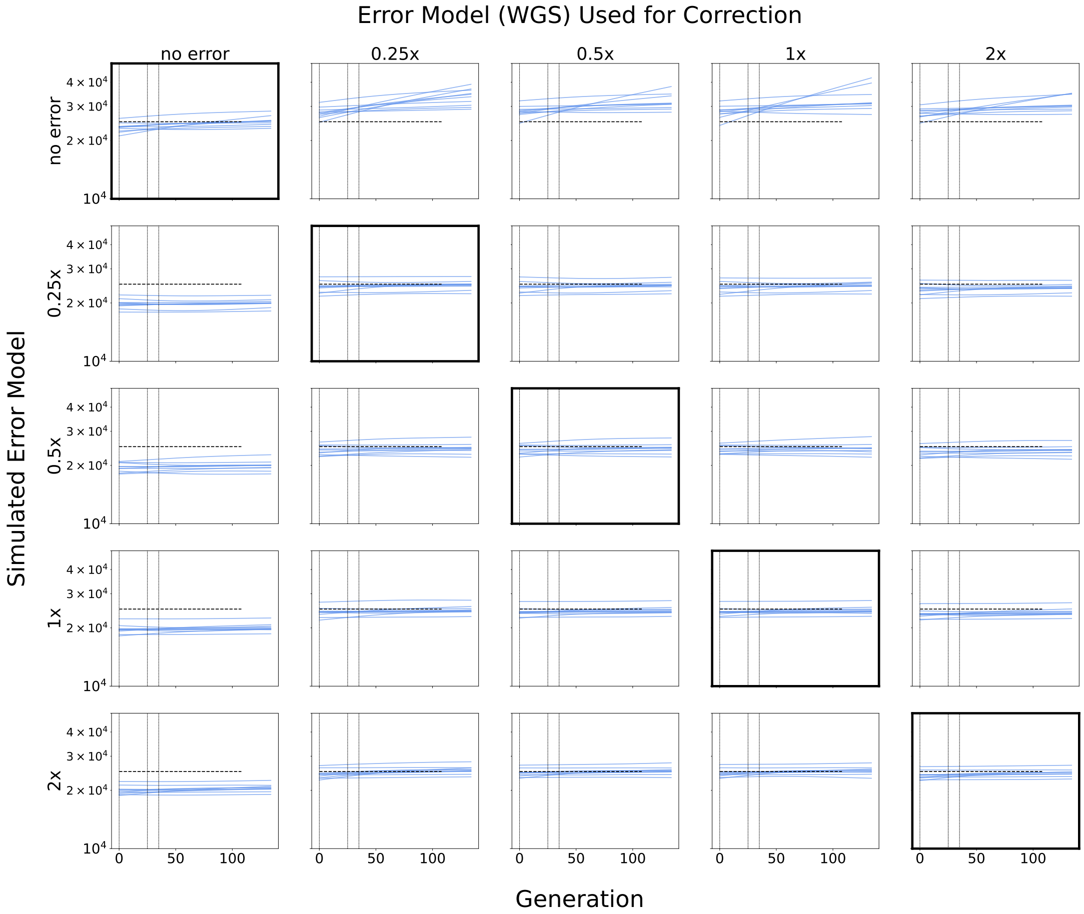

Figure S11: Inferred N_e of a constant demography with various simulated WGS-like error models This is the same constant demography as in main Fig.3.

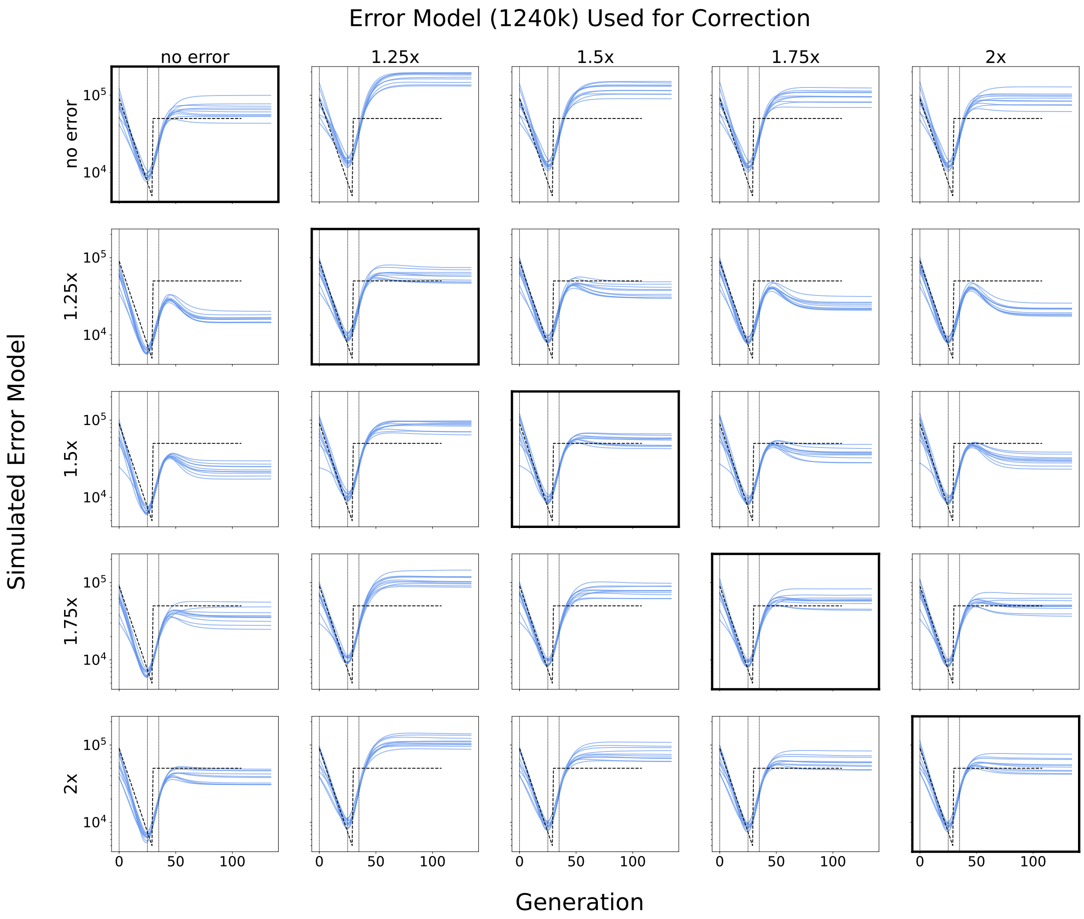

Figure S12: Inferred N_e of a bottleneck demography with various simulated 1240k-like error models

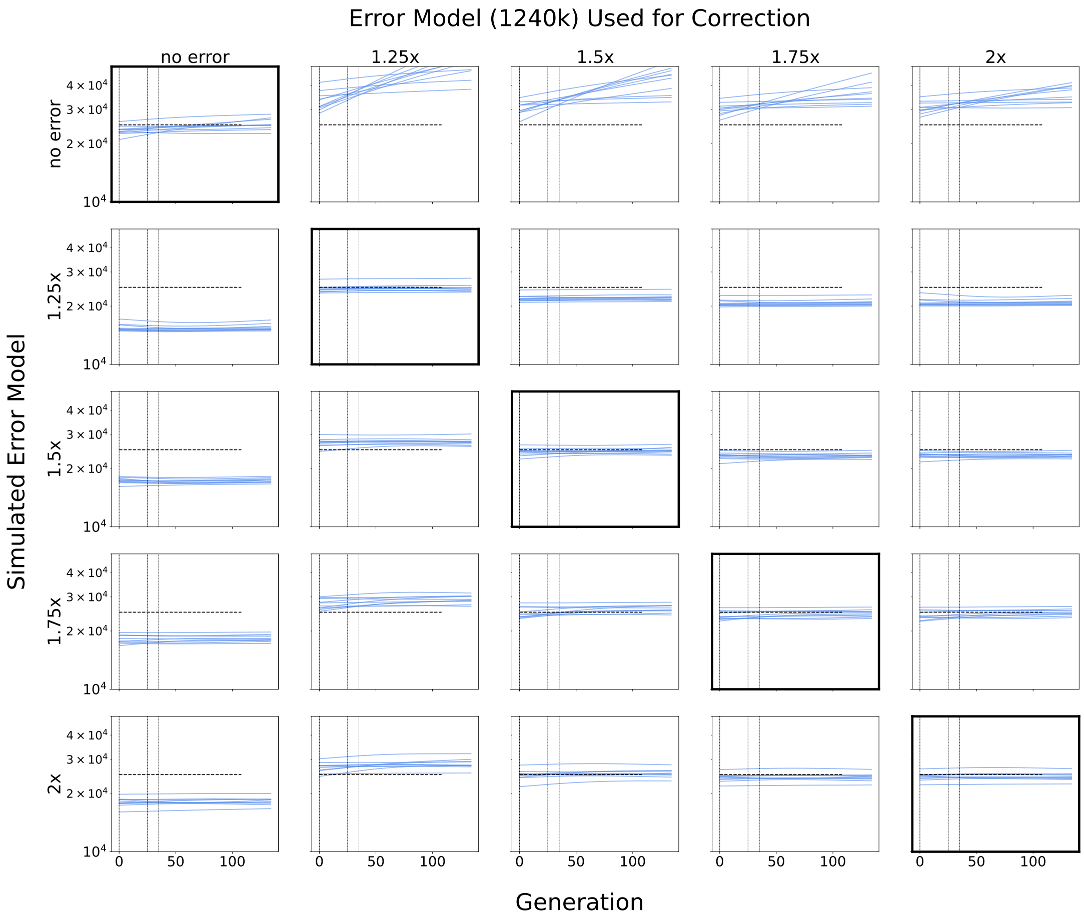

Figure S13: Inferred N_e of a constant demography with various simulated 1240k-like error models

Supp. Note S5 Simulating Bronze-age Demography

To aid the interpretation of our results on the inferred demography of the Corded ware groups, and in particular, to investigate how relatively recent admixture would affect the interpretation of inferred $\mathbf{N_e}$, we performed simulations using the current knowledge of Bronze Age European demography. Our Bronze Age demography (Fig.S14) is adapted from the AncientEurope_4A21 demography from stdpopsim [Adrion et al., 2020]. For our purpose, we made three changes. We set the admixture date between YAM and NEO to 170 generations before present (the original value is 140) to more accurately align with the estimated admixture date in Corded ware samples (3000–2900 BCE, Chintalapati et al. [2022]). We set the admixture date between CHG and EHG (i.e, the formation of the Yamnaya ancestry) to 200 generations before present (the original value is 180) so that it is more consistent with the empirically estimated admixture date of 4100BCE [Chintalapati et al., 2022]. We also added migrations between Bronze and both of its source populations (YAM and NEO) at a rate of 5% per generation for a period of 10 generations post admixture from $t=160$ to $t=170$.

The goal of this simulation is to examine how admixture, which violates our modeling assumption of a single, well-mixed population, affects the inferred effective population size. We note that, in the period before admixture, there does not exist a well-mixed single population ancestral to the sampled Corded Ware individuals; however, the so-called coalescent rates for the lineages sampled from Corded Ware individuals are always well-defined. For the particular demography simulated here, during the time before the admixture between YAM and NEO that gave rise to the Corded Ware complex, the effective population size of Corded Ware does not correspond to any populations per se; rather, it is simply the inverse of the coalescent rate. One can also think of the estimated effective population size at $t=160-170$ as the N_e of a hypothetical meta-population composed of the two admixing sources and the admixed population with limited gene flow among them. At $t>170$ and t less than the origin of both NEO and YAM, because the lineages from CW trace back to either NEO or YAM, the estimated effective population size is

$$\frac{1}{\alpha^2 \frac{1}{2*N_e(\text{NEO})} + (1-\alpha)^2 \frac{1}{2*N_e(\text{YAM})}},$$

where α is the admixture proportion of the source NEO. In short, when our assumption of a single, well-mixed population is not met, the inferred effective population size should be interpreted as the inverse of the coalescent rate (see related ideas in Chikhi et al. [2018], Boitard et al. [2022]). This quantity is always well-defined, but one should be cautious not

to take the inferred effective population size at its face value.

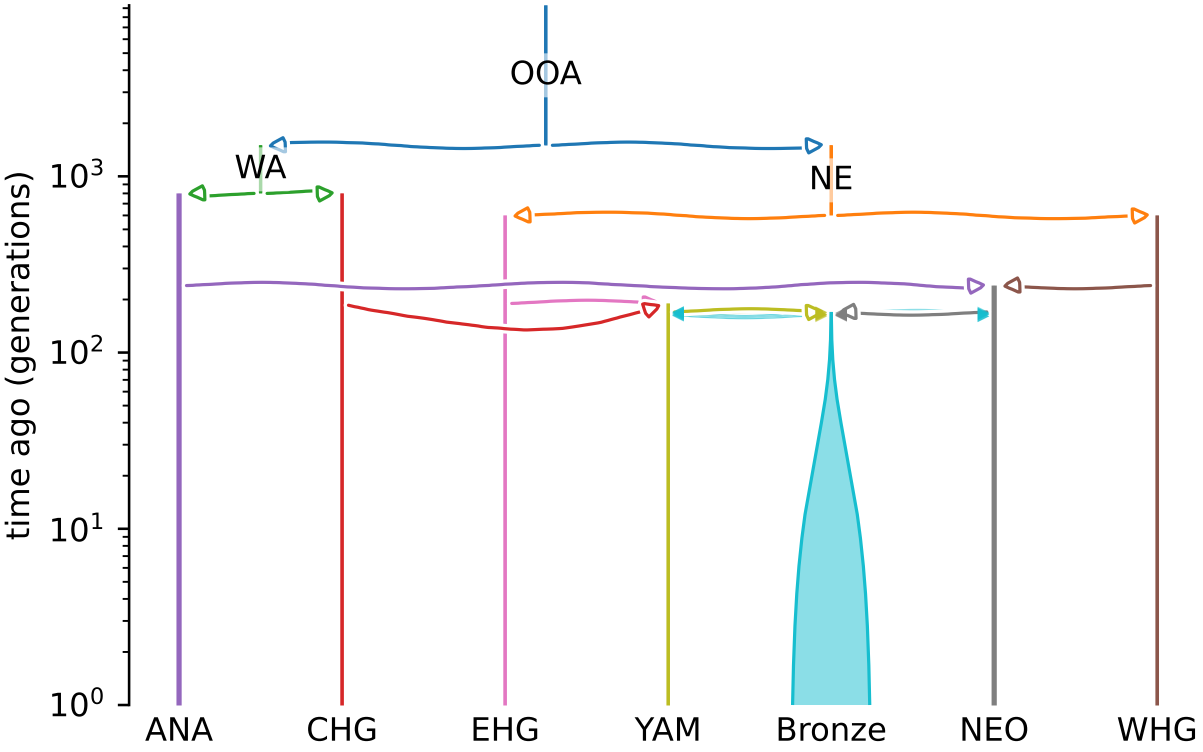

Figure S14: Simulated Demography of Bronze Age Populations A schematic of simulated demography of European Bronze Age populations, which is a proxy for Corded ware. OOA:out of Africa. NE: Northern European. WA: West Asia. ANA: Anatolia. CHG: Caucasus Hunter-gatherers. EHG: Eastern Hunter-gatherers. YAM: Yamnaya. NEO: Neolithic. WHG: Western Hunter-gatherers.

We simulated this Bronze Age demography following the same approach described in the main text. The ground truth $\mathbf{N_e}$ trajectory is calculated as the inverse of the coalescent rate for two lineages sampled from the Bronze population (function coalescence_rate_trajectory() from the debugger object of MSPRIME's demography class). The Bronze population samples were taken at $t=140, 145, 150, 155$ (indicated by vertical dashed lines in Fig.S15), which roughly corresponds to the sampling distribution of empirical Corded Ware samples used in this study. We note that inferred $\mathbf{N_e}$ at the time period before admixture ($t>170$) generally follows the inverse of coalescent rate, as explained above, although due to regularization and the fact that coalescence signal rapidly diminishes as one goes deeper in time, our methods underestimate $\mathbf{N_e}$ (or equivalently, overestimate the inverse coalescent rates). We also observe that with shorter IBD segments (e.g., $\geqslant$2cM), the inverse coalescent rates at deeper time depth can be better estimated. This is expected because longer IBD segments provide less information about deeper history. Therefore, the inferred $\mathbf{N_e}$ after certain time period is merely driven by regularization. The fact that with typical aDNA data quality we can only call relatively long IBD segments is a double-edged sword. On

the one hand, this means we have little power to recover population size beyond a few dozen generations. On the other hand, this makes our method robust to biases resulting from demographic events older than a few dozen generations from the sampling time, as long IBD segments exclusively originate from the most recent past.

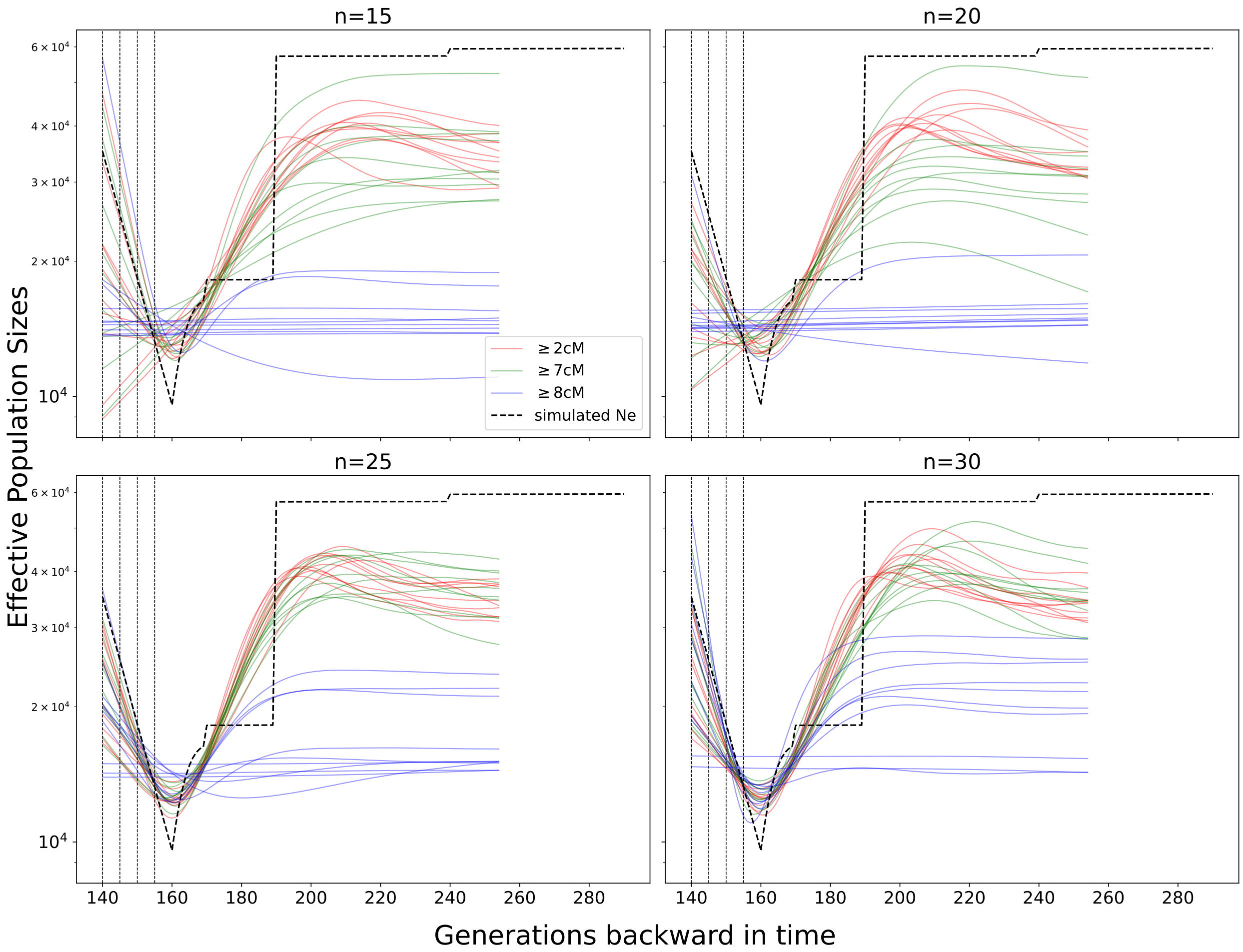

Figure S15: Inferred N_e of simulated Bronze Age populations using multiple sample sets. We applied TTNE to Bronze samples taken at four different time points (indicated by vertical dashed lines). We inferred N_e with four different IBD length cutoffs. The sample sizes in the sub-figure titles indicate the number of samples taken at each sampling time point. For example, for $n=15$, 60 samples are taken, evenly distributed over the 4 sampling points.

Additional Supplementary Figures

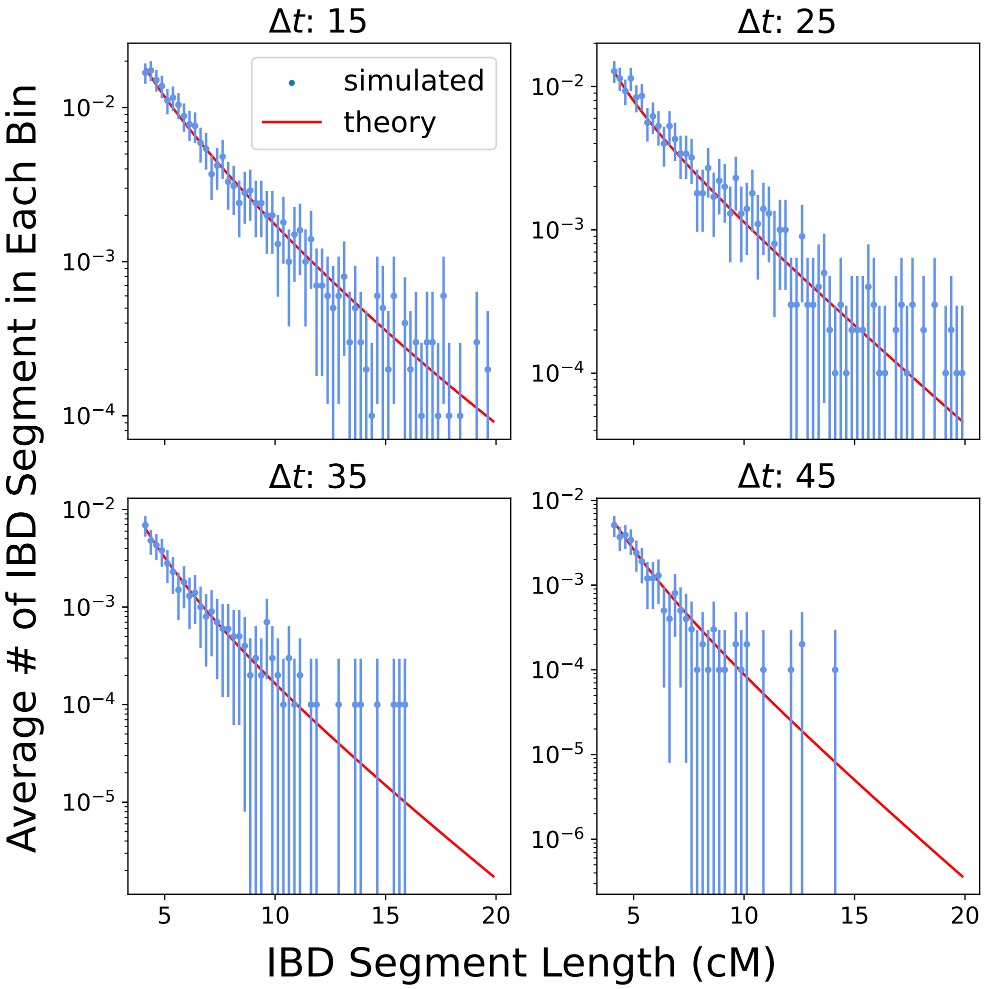

Figure S16: Simulated IBD and theoretical prediction. Same as main Fig.2c, but we also depict 95% confidence intervals of the number of IBD segments in each length bin as error bars.

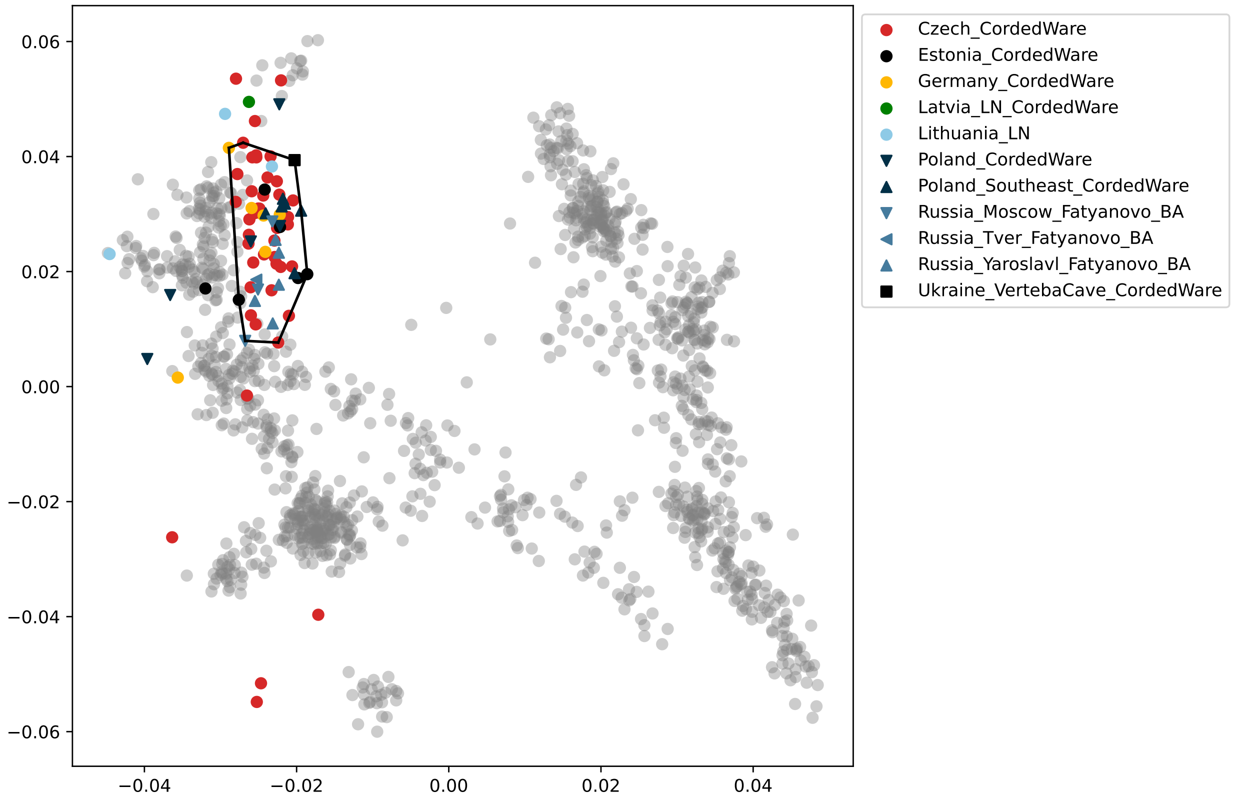

Figure S17: PCA of published Corded Ware samples. Principal Component Analysis (PCA) of published Corded Ware samples passing coverage requirement for HapNe-LD (having at least 300k 1240k SNPs covered, as recommended in Fournier et al. [2023]). The gray dots are the typical West-Eurasian Human Origins samples widely used as a PCA reference dataset for aDNA studies [Mallick et al., 2024]. The black boundary indicates the CW genomes we used to infer population size trajectory. We exclude individuals falling outside the black boundary as ancestry outliers. For HapNe-LD analysis, we used all samples with at least 300k 1240k SNPs covered, as recommended Fournier et al. [2023]. For ANCIBD analysis, we included all samples with at least 600k 1240k SNPs covered, a subset of samples shown in this PCA plot.

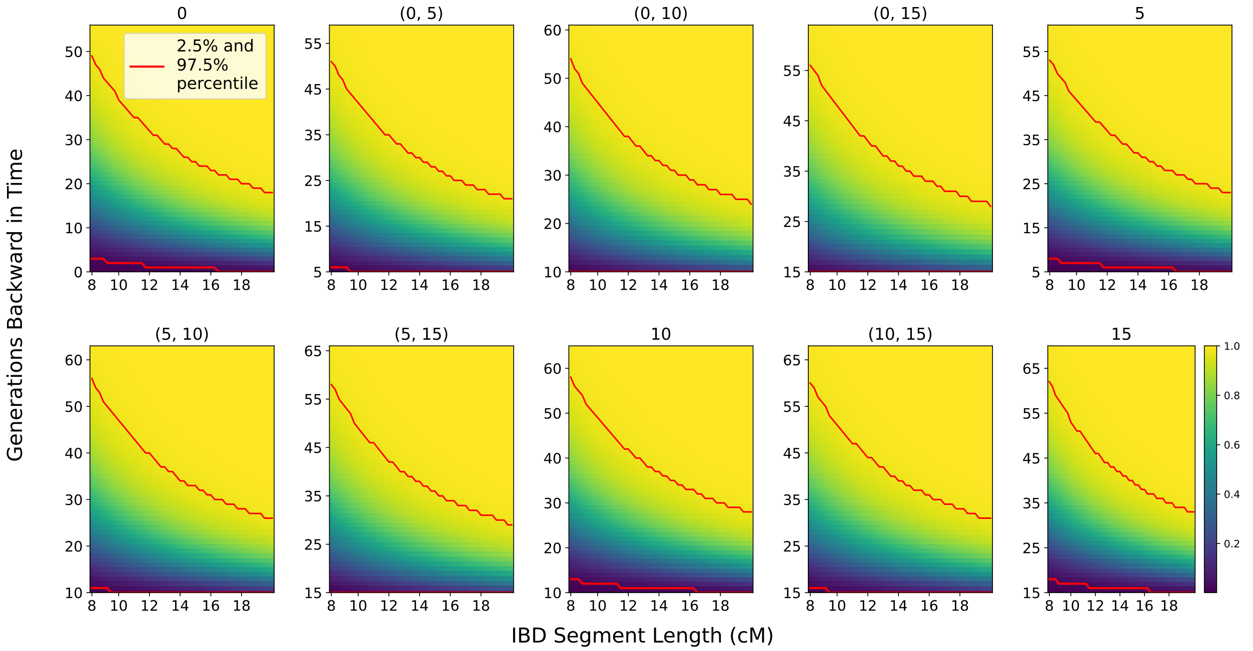

Figure S18: Cumulative Density of TMRCA for IBD segments of various lengths. Cumulative density plot of TMRCA of IBD segments of various lengths (x-Axis) calculated when using the inferred CW demography from IBD-sharing in CW individuals (see Fig. 5 in the main text). The two red lines depict the 2.5% and 97.5% percentiles.

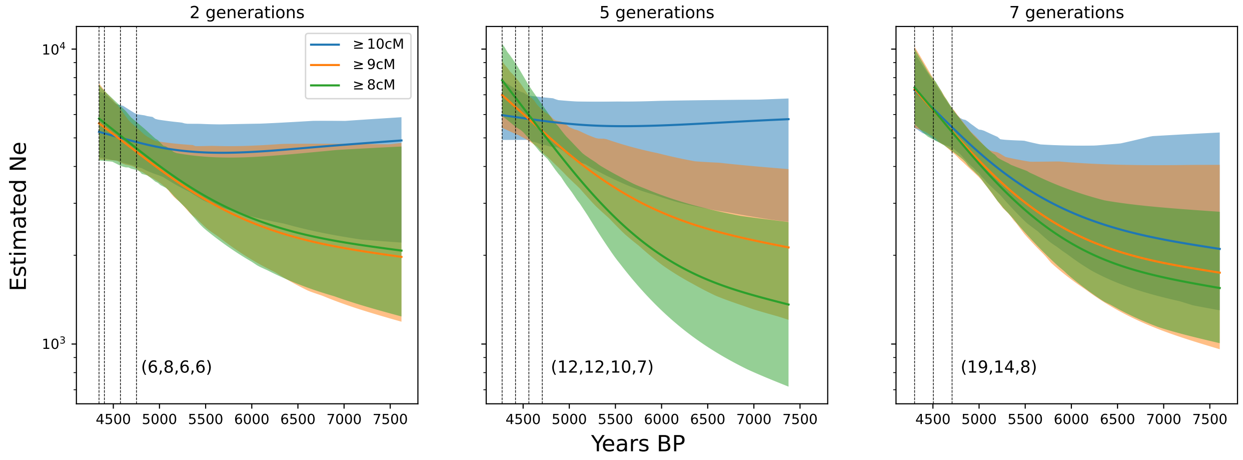

Figure S19: Inferred CW N_e trajectories across different levels of sample binning. We tested different levels of sample binning (e.g., grouping samples whose median radiocarbon date or archaeological context date are within 2,5,7 generations of one another) and inferred N_e trajectories. As a convention, we took 29 years as the generation time. The small text on the lower left of each sub-figure indicates the sample size at each sampling time point. A minimum of six samples is required for a group to be included in inference.

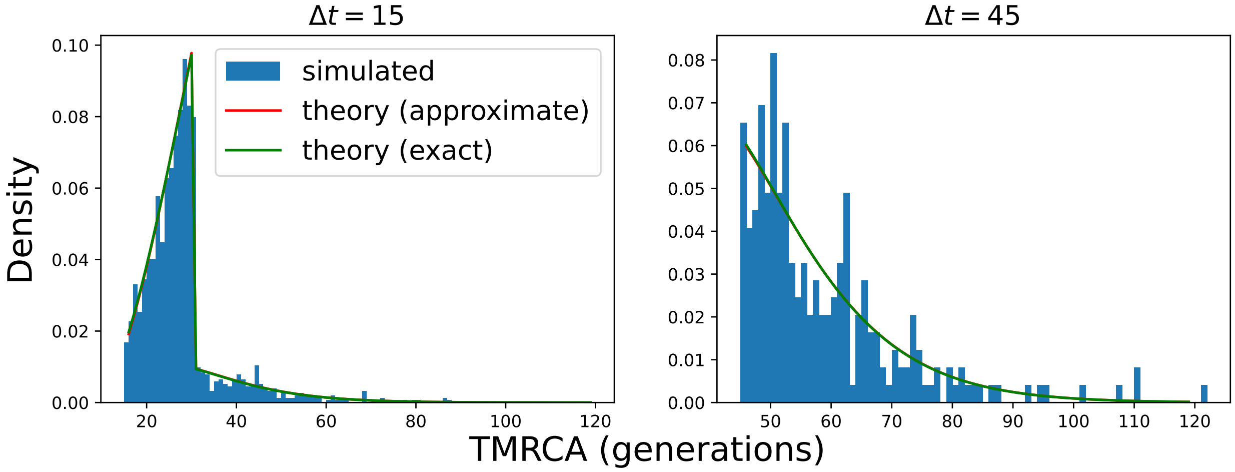

Figure S20: Comparing TMRCA of simulated IBD segments with analytical predictions. We simulated IBD segments under the bottleneck demography as in Fig.2b, except that we scaled the effective population size by a factor of 0.1 in order to obtain a sufficient number of IBD segments from 10,000 replicates to study the statistical properties of their TMRCA. We recorded the TMRCA of IBD segments shared between two haplotypes, one sampled at $t=0$, and the other at $t=15$ (left) or $t=45$ (right). We plotted the TMRCA distribution of simulated IBD segments with lengths between 5.75cM and 6.25cM as a histogram (in blue) and superimposed posterior TMRCA distribution theoretically calculated as described in Methods (the exact calculation in green and the approximate calculation in red, with segment length $l=6cM$), showing that the theoretical calculation matches the empirical simulations.

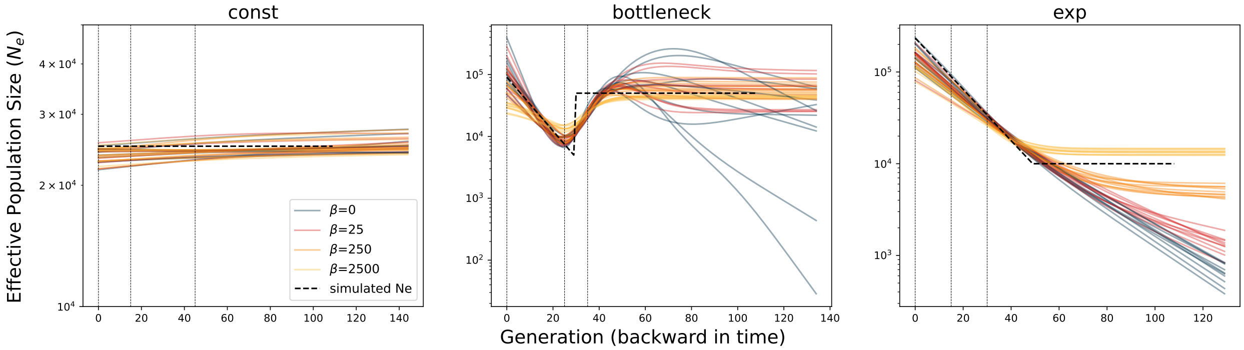

Figure S21: Effects of varying β on the inferred $\mathbf{N_e}$. Results of inferred $\mathbf{N_e}$ (n=30) by varying β from 0 to 2500. We note that $\beta=250$ or $\beta=2500$ yield similar results, but too small values of (e.g., 0 or 25) make the $\mathbf{N_e}$ estimate unstable at deeper time depth. Because of the decaying weights we employed (Eq.9 and Eq.10 in the main text), the choice of β has negligible effects on the recent past, which is where most of the IBD signal comes from. Therefore, we think it is safe to fix $\beta=250$, as this value is big enough to guard against unstable $\mathbf{N_e}$ at deeper time depth, where one has essentially no signal from long IBD segments.

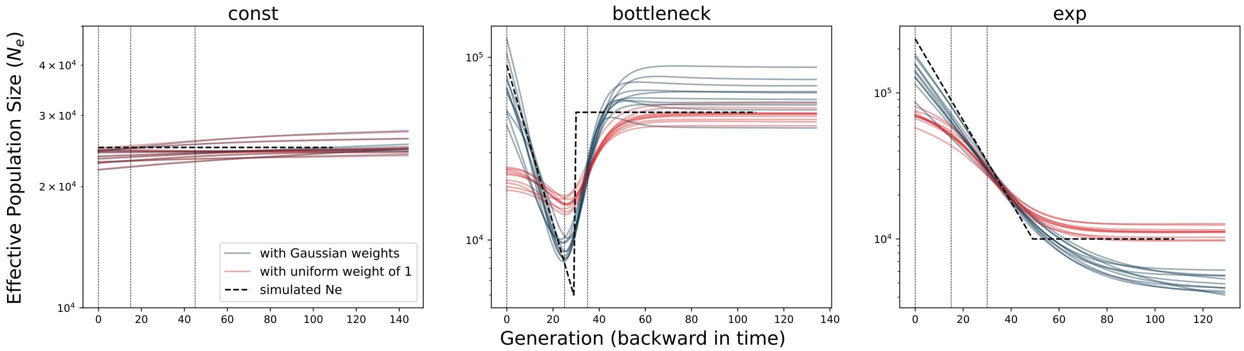

Figure S22: Effects of Gaussian weights on β. Comparison of inferred $\mathbf{N_e}$ (n=30) with the Gaussian positional decay (see Eq.9) and without (so that $w_t=1\forall t$). β is fixed at 250 for this experiment.

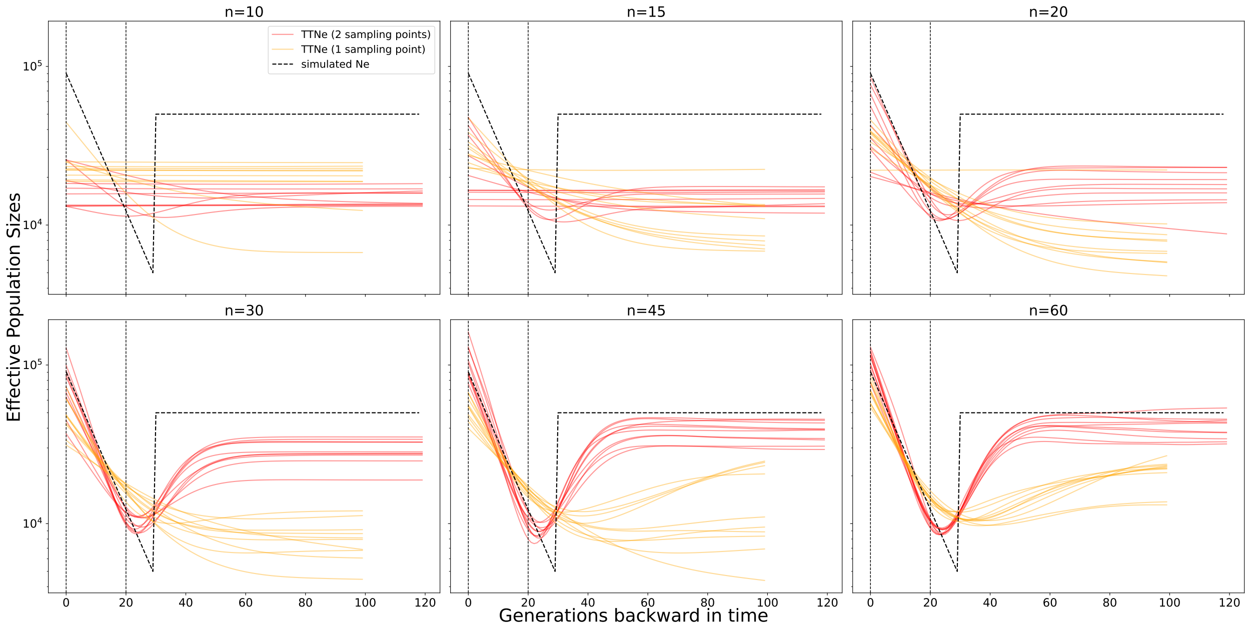

Figure S23: Inferring $\mathbf{N_e}$ in the bottleneck demography with two sampling points after a bottleneck event. Results of inferred $\mathbf{N_e}$ with two sampling points in the bottleneck demography (in red). The vertical dashed lines indicate the two sampling points. The subtitle indicates the sample size taken at each time point. As a baseline comparison, we also plot the results of inferred $\mathbf{N_e}$ using only contemporaneous samples (visualized in orange). The baseline has $3n$ samples at $t=0$. Although a comparison with $2n$ samples at $t=0$ is fairer, we did not conduct additional simulation with $2n$ samples as this does not seem necessary.

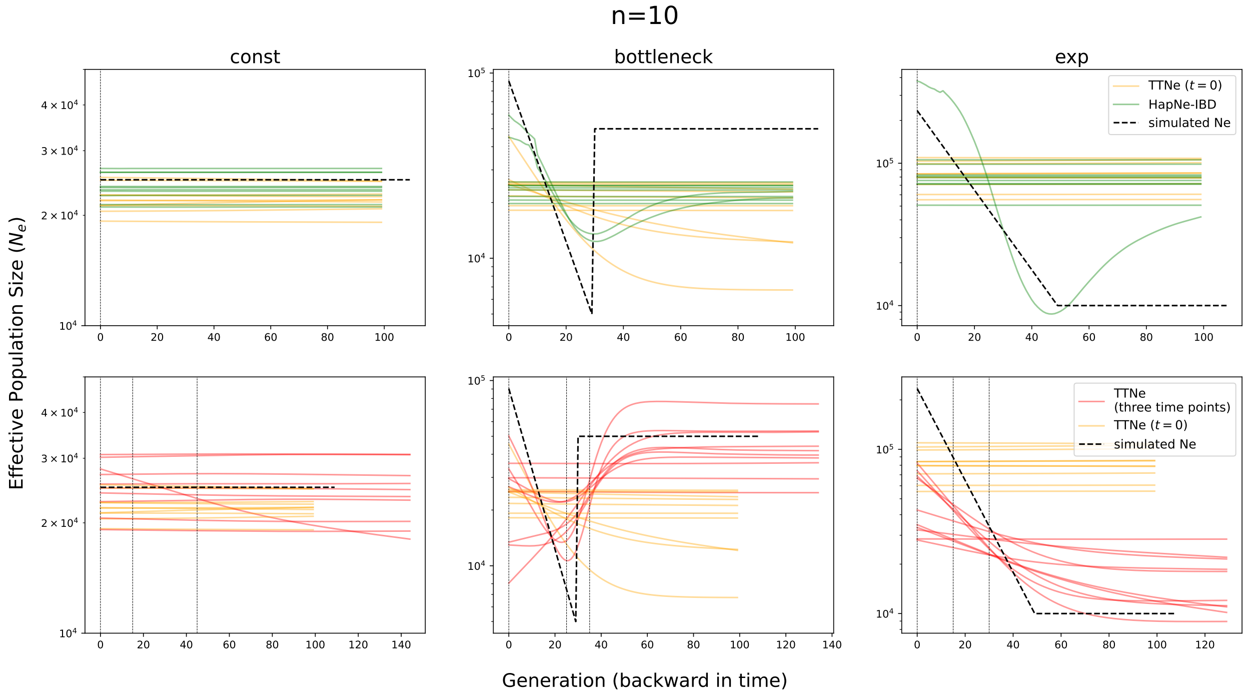

Figure S24: Performance of TTNE in various simulated demographic scenarios with $n=10$. Same as Fig. 3 in the main article but with $n=10$ at each sampled time point for models with multiple sampling points or $n=30$ for models with contemporaneous samples only.

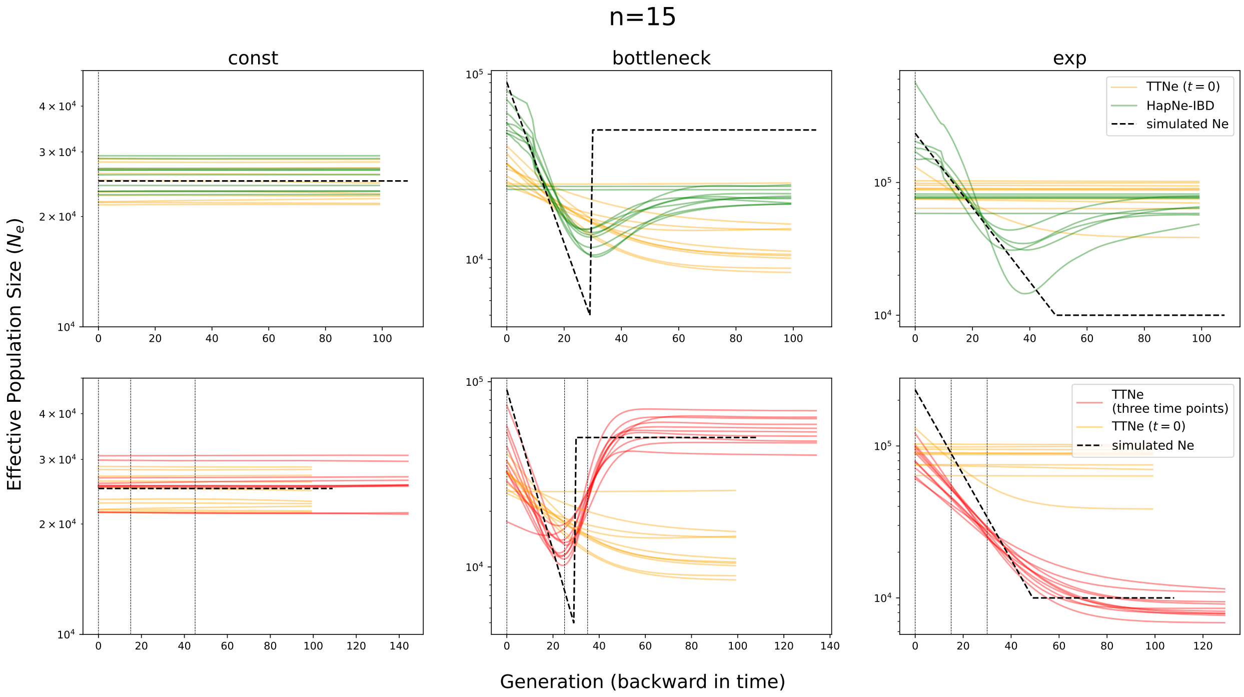

Figure S25: Performance of TTNE in various simulated demographic scenarios with $n=15$. Same as Fig. 3 in the main article but with $n=15$ at each sampling time point for models with multiple sampling points or $n=45$ for models with contemporaneous samples only.

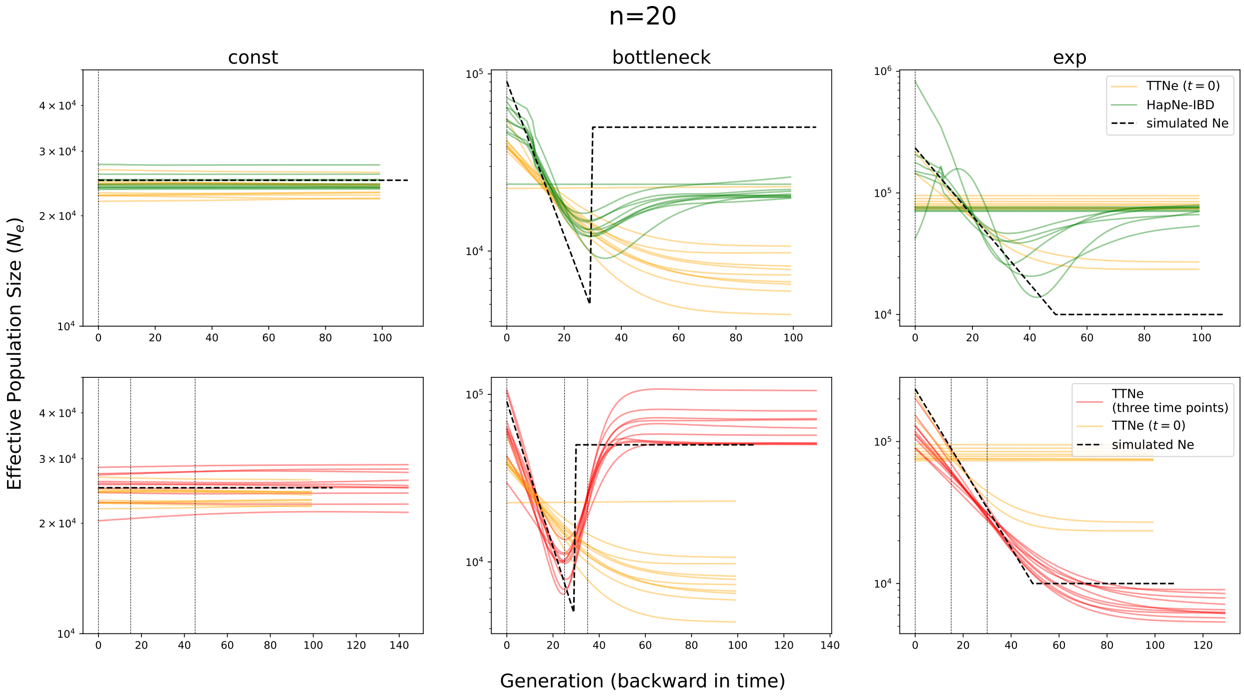

Figure S26: Performance of TTNE in various simulated demographic scenarios with $n=20$. Same as Fig.3 in the main article but with $n=20$ at each sampled time point for models with multiple sampling points or $n=60$ for models with contemporaneous samples only.

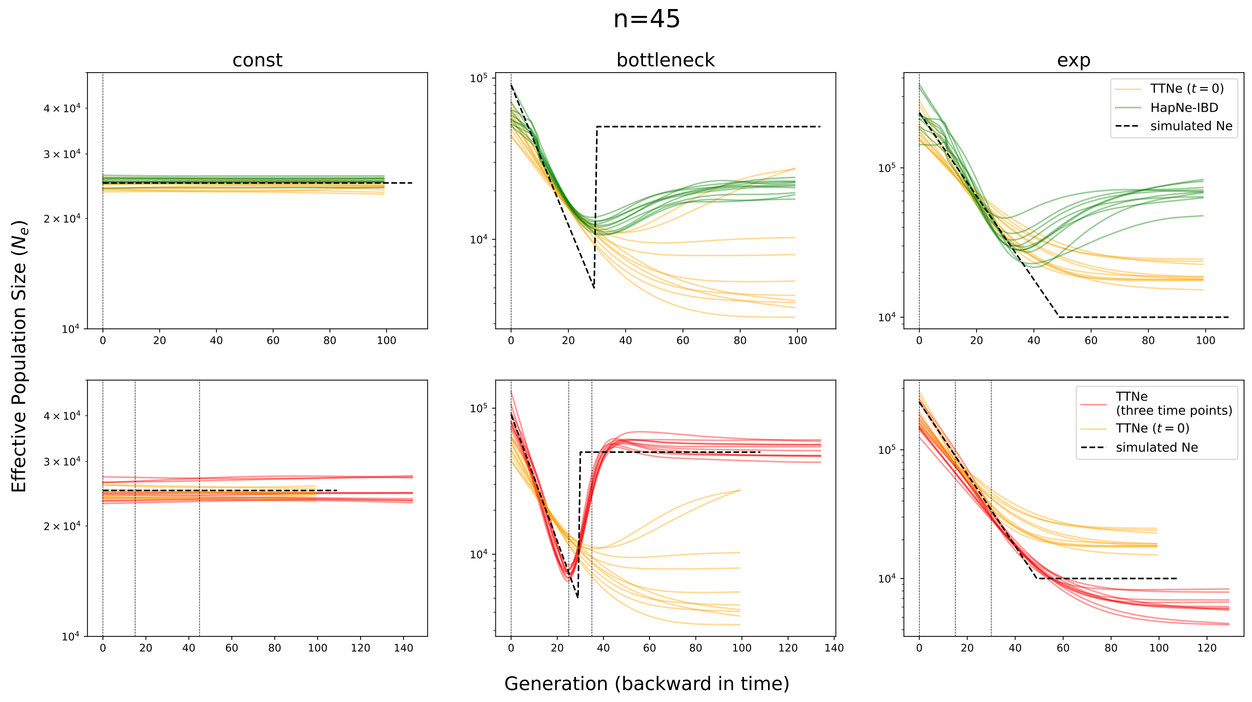

Figure S27: Performance of TTNe in various simulated demographic scenarios with n=45. Same as Fig.3 in the main article but with n=40 at each sampled time point for models with multiple sampling points or n=135 for models with contemporaneous samples only.

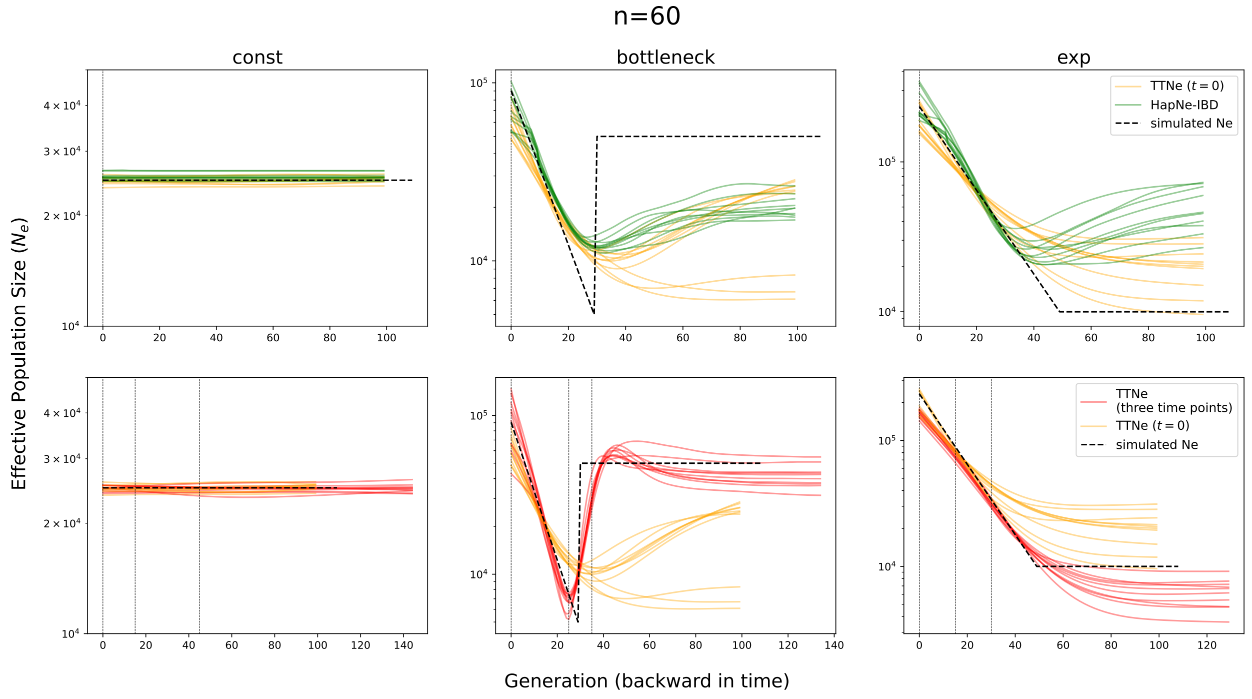

Figure S28: Performance of TTNe in various simulated demographic scenarios with n=60. Same as Fig.3 in the main article but with n=60 at each sampled time point for models with multiple sampling points or n=180 for models with contemporaneous samples only.

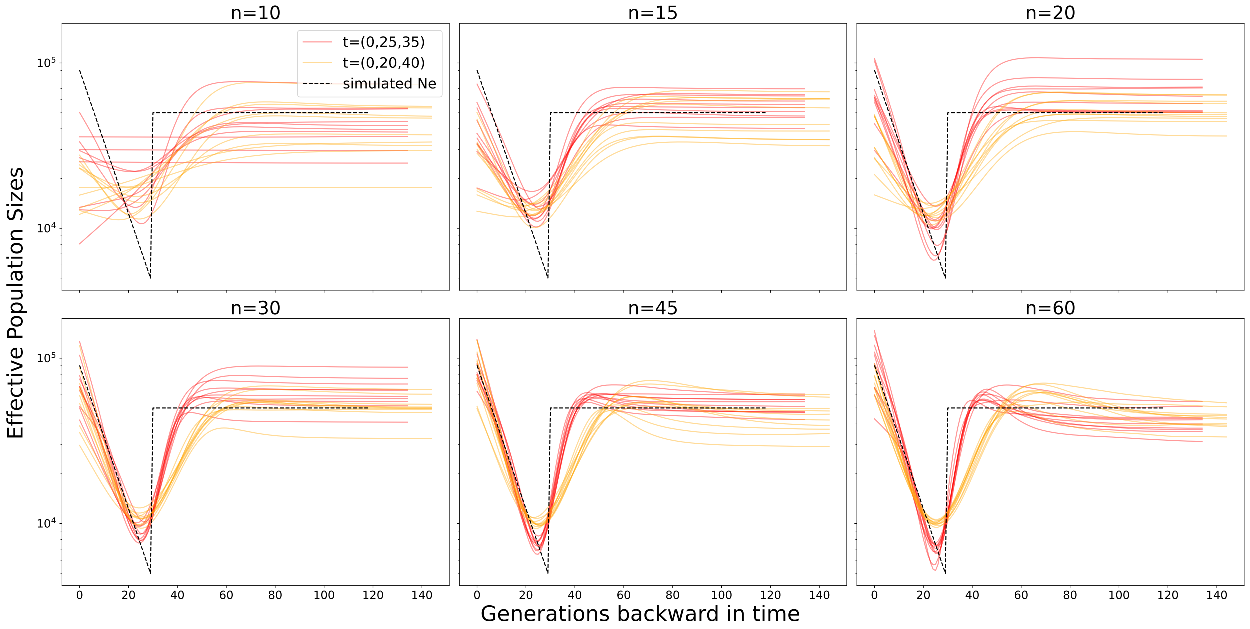

Figure S29: Performance of TTNE using two differing sampling strategies. For the simulated bottleneck demography, we designed two sampling strategies. The first is to take samples from $t = 0, 25, 35$ and the second is to take samples from $t = 0, 20, 40$. We found that the former performs better than the latter, especially with larger sample sizes.

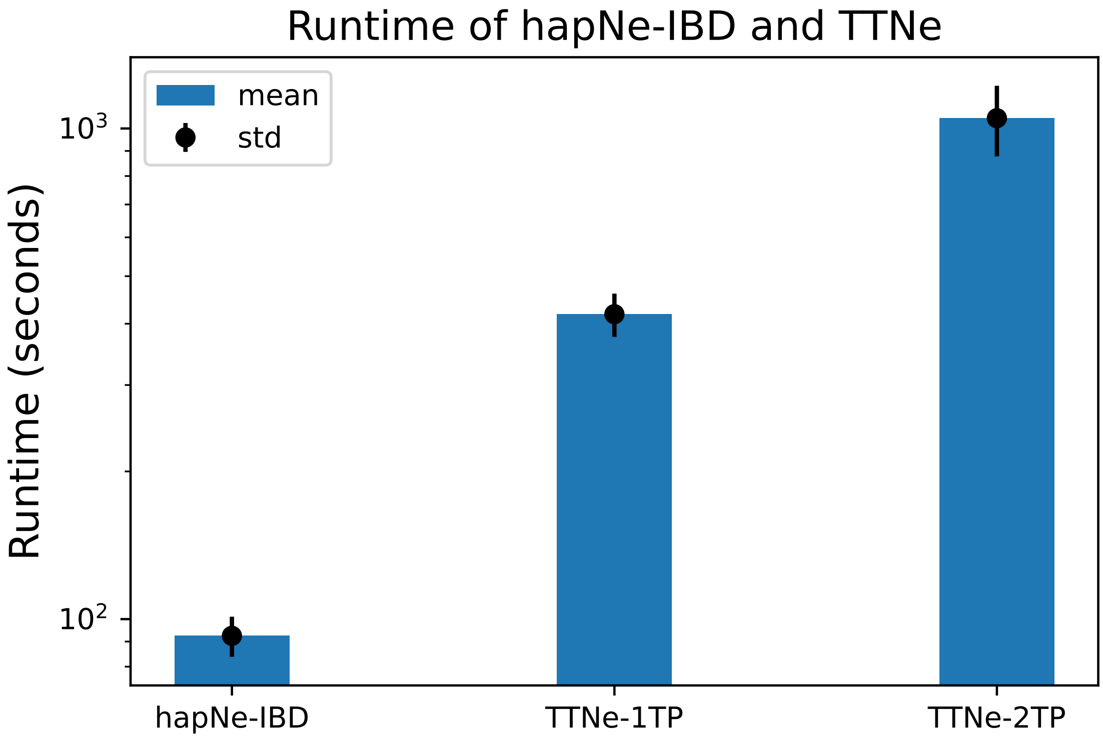

Figure S30: Runtime of TTNe in comparison with hapNe-IBD. Runtime of hapNe-IBD and TTNe using one and two sampling points. The runtime experiments were performed on AMD EPYC 7543 32-Core Processor and 8 processes were used to parallelize bootstraping. The average and standard deviation of runtime from 10 runs are shown in the bar plot. The runtime of TTNe only depends on the number of sample sets (the number of distinct time points from which samples are drawn), not on number of samples, because it only uses IBD histograms for inference. The same applies to hapNe-IBD as well. For both methods, the reported runtime includes the main iteration as well as hyperparameter search and bootstrap.

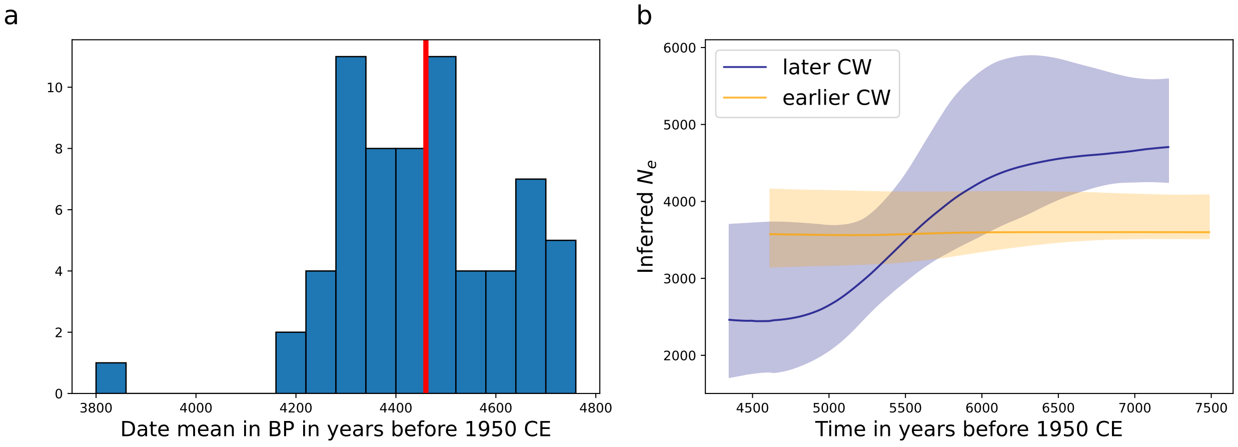

Figure S31: Inferring Corded Ware N_e using hapNe-LD a We plotted the histogram of the mean date (in BP in years before 1950CE) of individuals falling inside the main Corded Ware cluster (as shown in Fig.S17). We divided them into two groups (indicated by the thick red vertical bar), the earlier CW cluster consists of individuals dated before 4460BP, and the later CW cluster consists of individuals dated after 4460BP (but excluding VERT113B, an individual dated to 3823BP), and applied hapNe-LD separately to each of the two groups. **b** Inferred N_e of the earlier (in orange) and later CW (in navy) using hapNe-LD. For both groups, hapNe-LD warned about cross-chromosome LD, indicating that recent admixture may substantially bias the results.

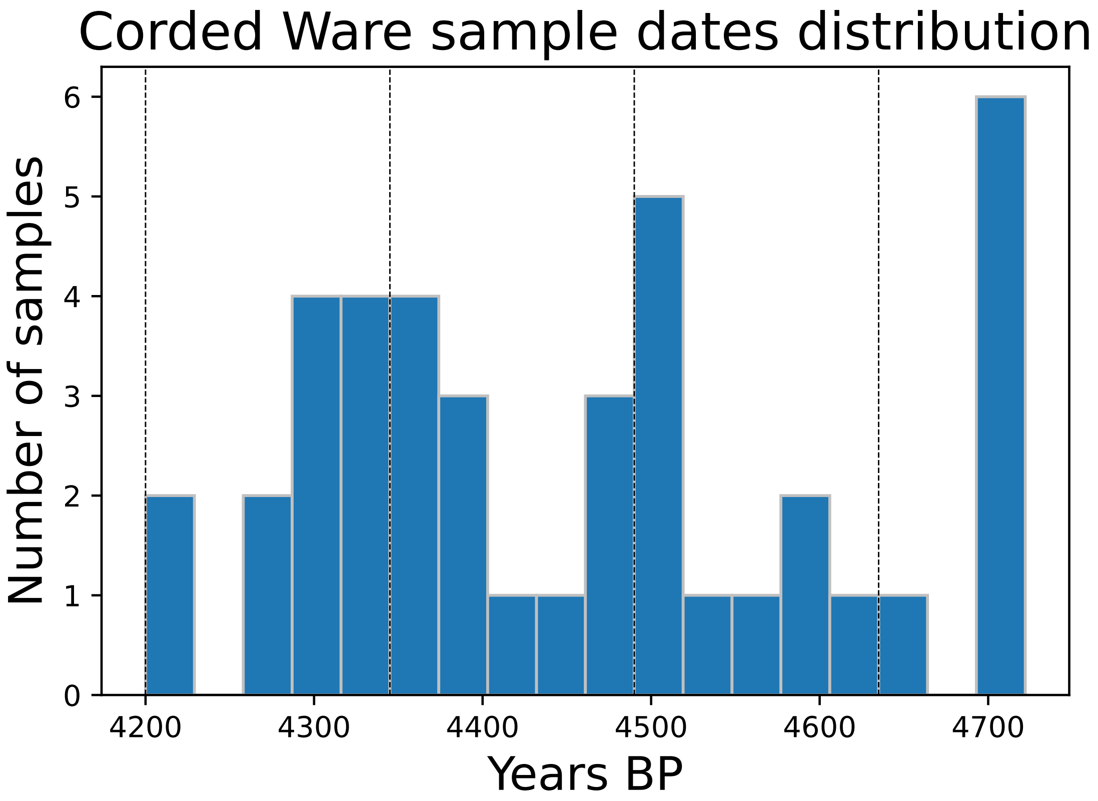

Figure S32: Date distribution of CW samples used for IBD analysis. Date distribution of CW samples used for IBD analysis and the grouping of temporally close samples. The dashed black vertical lines indicate the date delimiter of each temporal group. Each group spans 145 years (or five generations, assuming a generation time of 29 years).

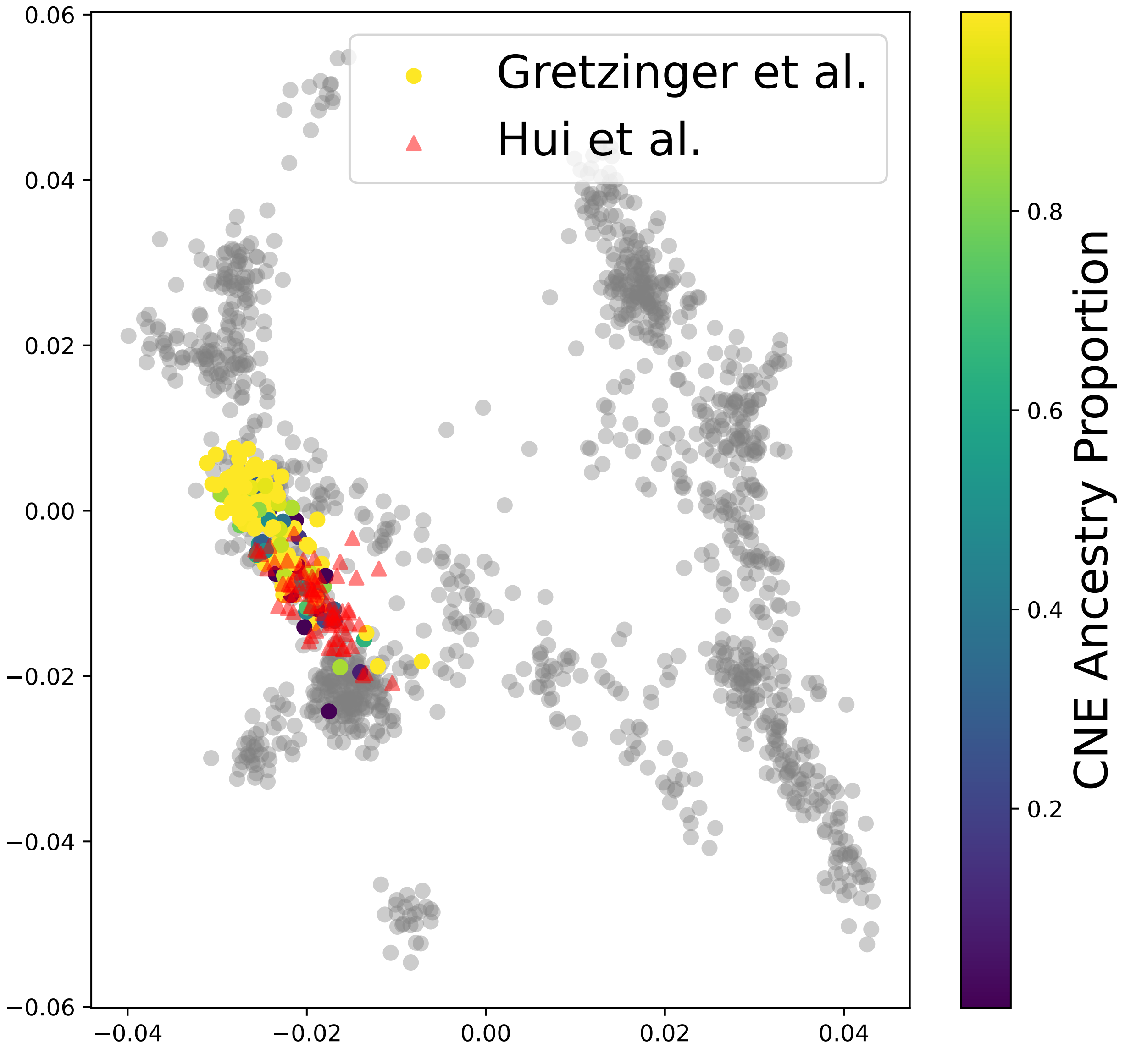

Figure S33: PCA of samples from the British Isle. PCA of samples from Gretzinger et al. [2022] and Hui et al. [2024]. For the samples from Gretzinger et al. [2022], we color-coded each individual according to its CNE (continental northern European) ancestry proportion. The CNE ancestry proportion was reported in Supplementary Table S3.7 in Gretzinger et al. [2022] by performing supervised admixture with source populations CNE and WBI (Western British and Irish).

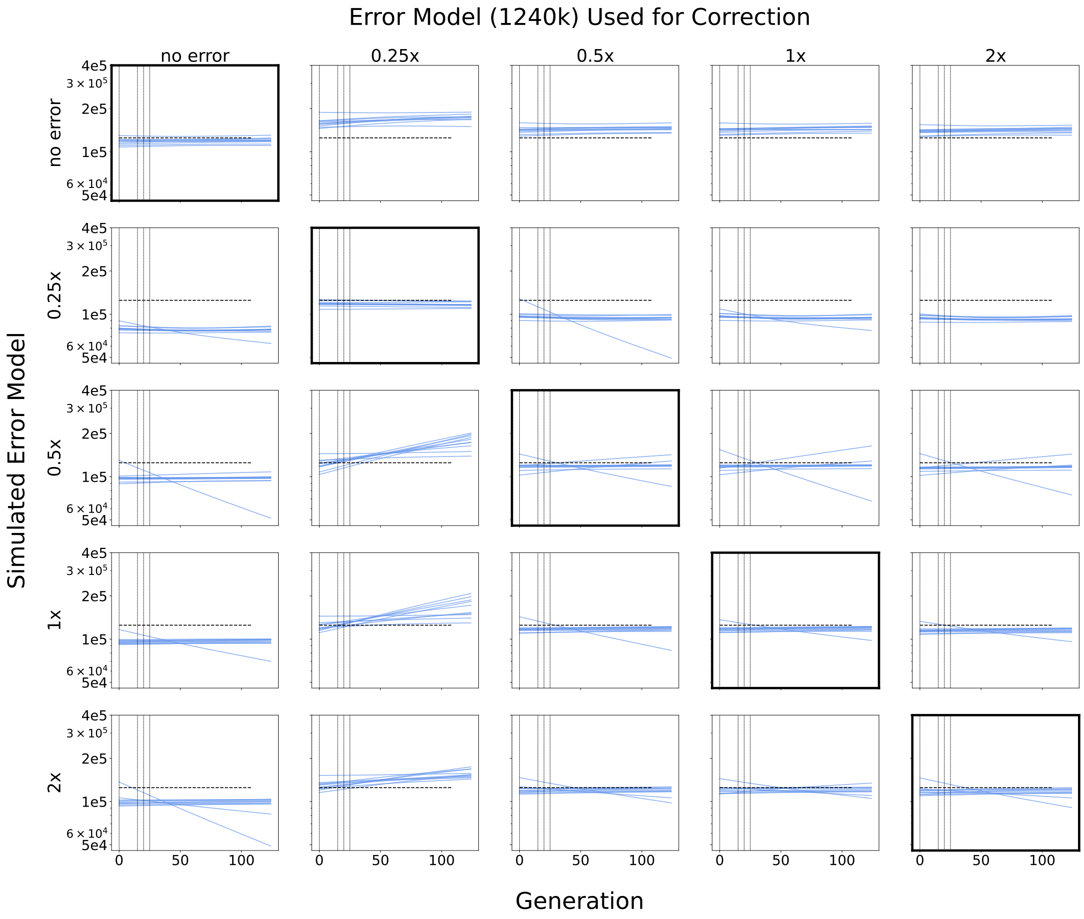

Figure S34: Inferred N_e of a constant demography (with N_e comparable to that of Medieval Britain) with various simulated wgs-like error models Simulation done as described in Supp. Note S4. The constant demography has a N_e=1.25e5, which was chosen explicitly to match the effective population size of empirical Medieval British samples used in this study (Fig.S36). The four sampling points are at t=0,15,20,25, each with sample size of 70,20,75,35, also chosen to explicitly match the sampling distribution of empirical British samples. This is to illustrate that the bias introduced by misspecified error model is dependent on the underlying population size. When the population size is small, the signal-to-noise ratio is high, and the model can tolerate greater levels of misspecification. This is mostly clearly seen by comparing this figure with Fig.S11.

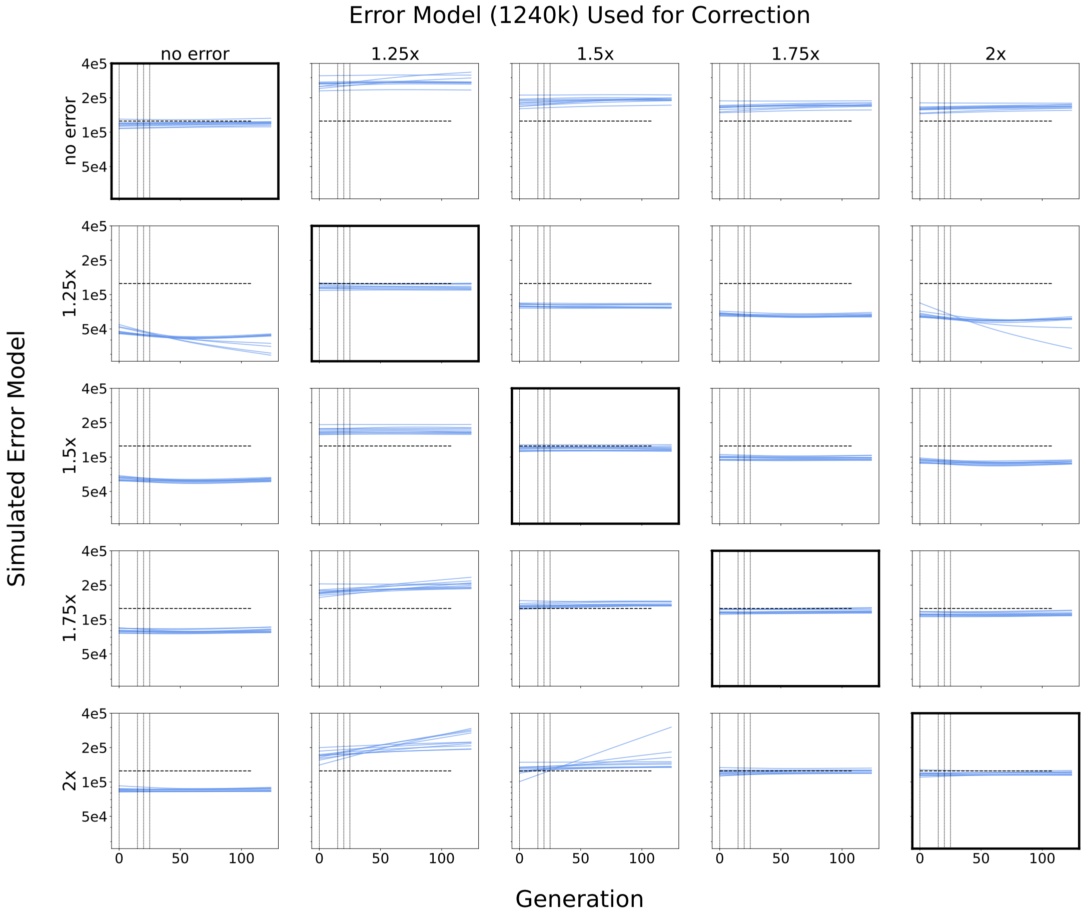

Figure S35: Inferred N_e of a constant demography (with N_e comparable to that of Medieval Britain) with various simulated 1240k-like error models Same as Fig.S34 but with 1240k-like simulated IBD detection errors.

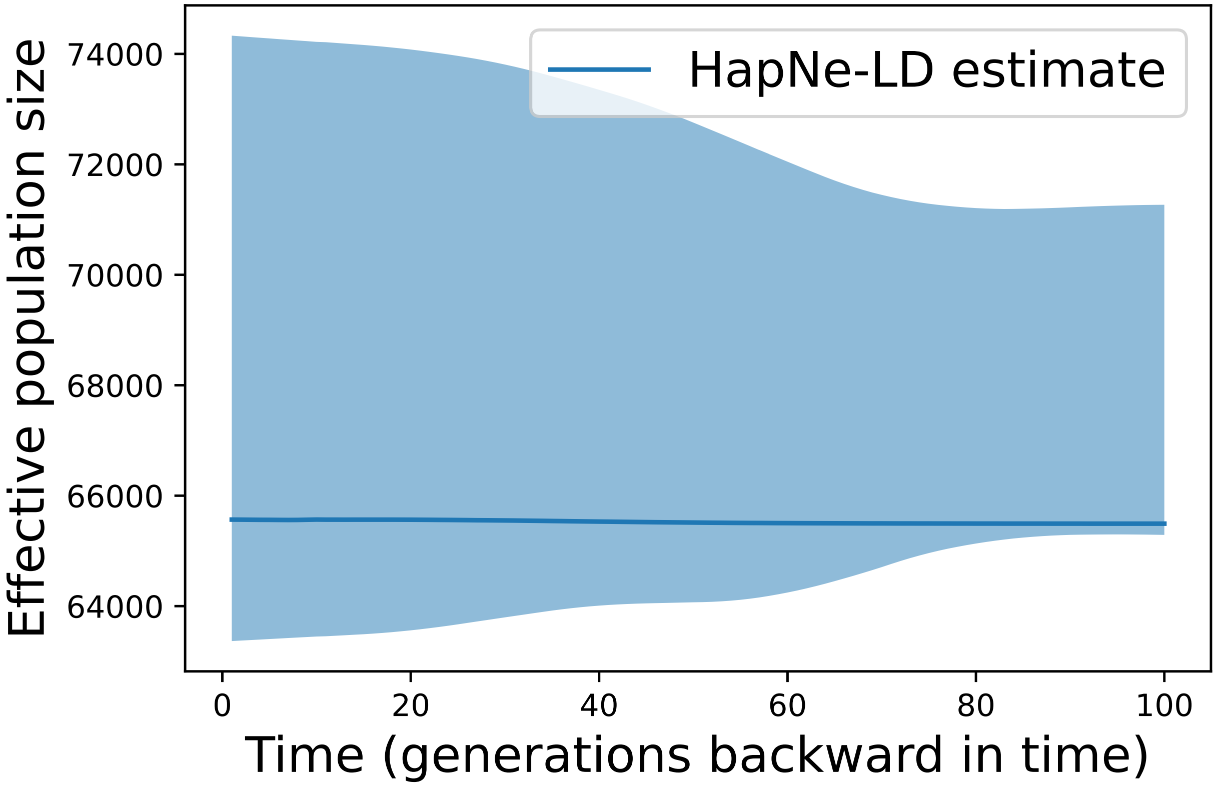

Figure S36: hapNe-LD inferred N_e using samples from Hui et al. [2024]

References

Jeffrey R Adrion, Christopher B Cole, Noah Dukler, Jared G Galloway, Ariella L Gladstein, Graham Gower, Christopher C Kyriazis, Aaron P Ragsdale, Georgia Tsambos, Franz Baumdicker, et al. A community-maintained standard library of population genetic models. *Elife*, 9:e54967, 2020.

Simon Boitard, Armando Arredondo, Lounès Chikhi, and Olivier Mazet. Heterogeneity in effective size across the genome: effects on the inverse instantaneous coalescence rate (iicr) and implications for demographic inference under linked selection. *Genetics*, 220(3):iyac008, 2022.

Lounès Chikhi, Willy Rodríguez, Simona Grusea, Patricia Santos, Simon Boitard, and Olivier Mazet. The iicr (inverse instantaneous coalescence rate) as a summary of genomic diversity: insights into demographic inference and model choice. *Heredity*, 120(1):13–24, 2018.

Manjusha Chintalapati, Nick Patterson, and Priya Moorjani. The spatiotemporal patterns of major human admixture events during the european holocene. *Elife*, 11:e77625, 2022.

Romain Fournier, Zoi Tsangalidou, David Reich, and Pier Francesco Palamara. Haplotype-based inference of re-

cent effective population size in modern and ancient dna samples. *Nature Communications*, 14(1):7945, 2023.

Joscha Gretzinger, Duncan Sayer, Pierre Justeau, Eveline Altena, Maria Pala, Katharina Dulias, Ceiridwen J Edwards, Susanne Jodoin, Laura Lacher, Susanna Sabin, et al. The anglo-saxon migration and the formation of the early english gene pool. *Nature*, 610(7930):112–119, 2022.

Ruoyun Hui, Christiana L Scheib, Eugenia D'Atanasio, Sarah A Inskip, Craig Cessford, Simone A Biagini, Anthony W Wohns, Muhammad QA Ali, Samuel J Griffith, Anu Solnik, et al. Genetic history of cambridgeshire before and after the black death. *Science Advances*, 10(3):eadi5903, 2024.

Swapan Mallick, Adam Micco, Matthew Mah, Harald Ringbauer, Iosif Lazaridis, Iñigo Olalde, Nick Patterson, and David Reich. The allen ancient dna resource (aadr) a curated compendium of ancient human genomes. *Scientific Data*, 11 (1):182, 2024.

Iain Mathieson, Songül Alpaslan-Roodenberg, Cosimo Posth, Anna Szécsényi-Nagy, Nadin Rohland, Swapan Mallick, Iñigo Olalde, Nasreen Broomandkhoshbacht, Francesca Candilio, Olivia Cheronet, et al. The genomic history of southeastern europe. *Nature*, 555(7695):197–203, 2018.

Iñigo Olalde, Selina Brace, Morten E Allentoft, Ian Armit, Kristian Kristiansen, Thomas Booth, Nadin Rohland, Swapan Mallick, Anna Szécsényi-Nagy, Alissa Mittnik, et al. The beaker phenomenon and the genomic transformation of northwest europe. *Nature*, 555(7695):190–196, 2018.

Harald Ringbauer, Yilei Huang, Ali Akbari, Swapan Mallick, Iñigo Olalde, Nick Patterson, and David Reich. Accurate detection of identity-by-descent segments in human ancient dna. *Nature Genetics*, pages 1–9, 2023.

Maïté Rivollat, Adam Benjamin Rohrlach, Harald Ringbauer, Ainash Childebayeva, Fanny Mendisco, Rodrigo Barquera, András Szolek, Mélie Le Roy, Heidi Colleran, Jonathan Tuke, et al. Extensive pedigrees reveal the social organization of a neolithic community. *Nature*, 620(7974):600–606, 2023.

Minze Stuiver and Gordon W Pearson. High-precision calibration of the radiocarbon time scale, ad 1950–500 bc. *Radiocarbon*, 28(2B):805–838, 1986.

Anthony Wilder Wohns, Yan Wong, Ben Jeffery, Ali Akbari, Swapan Mallick, Ron Pinhasi, Nick Patterson, David Reich, Jerome Kelleher, and Gil McVean. A unified genealogy of modern and ancient genomes. *Science*, 375(6583):eabi8264, 2022.
