# Supplementary material for: Estimating effective population size trajectories from time-series identity-by-descent segments
Source: Genetics. 2025 Jan 24;229(3):iyae212. doi: 10.1093/genetics/iyae212 (PMC11912830; doi:10.1093/genetics/iyae212)
Supplement: iyae212_Peer_Review_History [file iyae212_peer_review_history.pdf]

GENETICS

Estimating effective population size trajectories from time-series Identity-by-Descent (IBD) segments

Yilei Huang, Shai Carmi, and Harald Ringbauer

NOTE: The reviews and decision letters are unedited and appear as submitted by the reviewers.
In extremely rare instances and as determined by a Senior Editor or the EIC, portions of a review may be redacted. If a review is signed, the reviewer has agreed to no longer remain anonymous.
The review history appears in chronological order.

Review Timeline:

Submission Date:	2024-05-09
Editorial Decision:	2024-06-26
Resubmission Received:	2024-11-07
Editorial Decision:	2024-11-14
Revision Received:	2024-11-27
Accepted:	2024-12-12

1st Editorial Decision: June 26, 2024

June 26, 2024

GENETICS-2024-307074
Estimating effective population size trajectories from time-series Identity-by-Descent (IBD) segments

Dear Dr. Huang:

Three experts in the field have reviewed your manuscript, and I have read it in detail as well. All reviewers and myself agree that the manuscript describes some very interesting and useful technical work, demonstrating a potentially powerful method for population genetics. I would also like to appreciate the substantial and good work you've put into many of the technical aspects of this. While your manuscript is not currently acceptable for publication in GENETICS, we would welcome a substantially revised manuscript. Both reviewers have comments and concerns to be addressed in a revised manuscript. You can read their reviews at the end of this email.

There are two general classes of reviewer comments below that should be addressed in a revised manuscript: first, the main text needs substantial editing in places so that it accurately and unambiguously describes the methods. Second, the reviewers (particularly Reviewer 2) have many suggestions for how additional investigations would improve the robustness and wider applicability of the work, in particular around the error model. I would hope that a revised manuscript would address all of these concerns, either through additional exploration or through careful discussion of the issue. (For instance, a full exploration of the many factors affecting an error model is probably beyond the scope of this paper, but some more general guidance to using this aspect of the python package could be given, and perhaps even a well-described and simple procedure for estimating the parameters in the error model.) We acknowledge the review process has been lengthy so far, and hope that a revised paper can be produced without major additional experiments. We look forward to receiving your revised manuscript. Please let the editorial office know approximately how long you expect to need for revisions.

Upon resubmission, please include:
1. A clean version of your manuscript;
2. A marked version of your manuscript in which you highlight significant revisions carried out in response to the major points raised by the editor/reviewers (track changes is acceptable if preferred);
3. A detailed response to the editor's/reviewers' feedback and to the concerns listed above. Please reference line numbers in this response to aid the editor and reviewers.

Your paper will likely be sent back out for review.

Additionally, please ensure that your resubmission is formatted for GENETICS
https://academic.oup.com/genetics/pages/general-instructions

Follow this link to submit the revised manuscript: Link Not Available

Sincerely,

Peter Ralph
Associate Editor
GENETICS

Approved by:
David Begun
Senior Editor
GENETICS

Associate Editor comments for the Authors:

I appreciate the exploration of various regularization strategies! Some discussion of how future work might improve on this would be helpful. Also, I'm surprised that you can find so much very long IBD among these ancient samples - although I probably should not be, given the smaller sample sizes. This is a good thing, because shorter IBD would be substantially noisier. I think this is a neat and interesting

result that could be highlighted in the abstract or Introduction.

By my reading (apologies if I've missed it), there is not sufficient description of what (in particular: how many) samples were used in each of the empirical applications. Sample sizes should be given in the main text, and a table of sample identifiers and estimated dates should be provided in the Supplement.

Equation (1): Here the function f( ) on the two sides of the equation mean different things. If taken literally, equation (1) suggests that there is a single function f( ) that is evaluated with two different arguments, N_l^t and K_l^t, on the two sides of the equation. A more usual way to denote what the authors want to say here would be to define, say, f_N(l,t) to be the expected number of segments of length l from time t ago, and similarly f_K(l, t). (I see that maybe the authors are following the notation of Ringbauer et al but replacing \mathbb{E}[ ] with f( ); however, for this to make sense one needs to at minimum define the random variables N_l^t and K_l^t.)

Equation (1): Units should be more precisely specified for both time and length: is this a density?

Equation (1): the factor from K is not "the average number of uninterrupted ancestry blocks", although it is related to that.

Above equation (2): the text says "\Delta t" but the inline equation has only \Delta.

Equation (2): If looking for additional justification for this modification to Ringbauer, you can cite Ralph & Coop, which is phrased more generally than Ringbauer (to obtain this equation, differentiate equation (6) in Ralph & Coop with respect to x).

Equation (9): Script N is not defined.

l.185: The expression that involves both a numpy expression in python and an undefined script N denoting a probability density should be written out clearly.

Several places: "principal component analysis" not "principle component analysis"

Reviewer #1 (Comments for the Authors (Required)):

Summary:
This manuscript describes a new method, TTNe, for inferring population size history from IBD distribution of time-stratified genomic sequences. They derived the expected IBD distributions across time under a given Ne(t), then use the likelihood function (with penalty terms) to estimate optimal \hat{Ne}(t) with a SciPy's built-in optimization algorithm. They showed with coalescent simulations that their framework can reliably predict IBD distributions, and the subsequent Ne(t) inference are also satisfactory. The authors then applied TTNe on human aDNA data, producing inferences consistent with previous studies.

Major comments:
- Although the authors later considered admixture in empirical analyses and further discussed it in Discussion, it's not addressed in simulation studies. Even though all methods for inferring pop size history are inevitably affected by violations of assuming the population to be panmictic, it would still be nice to see how susceptible they are compared with each other.

- When it comes to temporal genetic data, one issue that frequently comes up is the sampling scheme design. Although it is relatively non-negotiable for aDNA datasets, geneticists studying experimental evolution would greatly benefit from advice on (even just rules-of-thumb) appropriate sampling intervals and sample sizes. I noticed that the authors chose different sampling times for the 3 demographic models in the simulation, were they just arbitrarily decided or is there any justification for choosing these time points? I see that the authors have explored further on this topic in Supp. Note S2.3, where (in my humble opinion) the main findings in this section should be mentioned in the main text.
- I also wonder how much the DNA decays in aDNA datasets affect downstream inferences. I see that it was later discussed in Supp. Note S2.2, though I think this issue is worth addressing in the main text.
- It might be less of a concern from a theoretical/modeling perspective, but I'm curious about the relative computational performance of TTNe (say, compared with HapNe-IBD).

Minor comments:
- Line 110: typo? $2t - \Delta$ is missing a t
- Fig 2c: it'd also help to show how the framework aligns with simulation to plot the $\Delta_I(t)$ as shades around the theoretical lines
- Eq. 9: is there a particular reason for $\sigma = T_{max}/3$? Why?
- Line 283--5: why aren't the sampling times identical across all three demographic models? How does the choice of sampling time affect the inference?
- Line 329: the latter half of this sentence reads very confusing to me. Is the "> 12cM" supposed to be removed?
- Line 349: missing citation
- Line 393: the en-dash ("--") should be em-dash ("---") here
- Line 417: typo?
- About how to address selection from black death affect the inference: have the authors tried excluding certain well-known immune-related genomic areas (e.g., HLA complex, and/or reading frames of all immune-related genes?), and see how much the Ne(t) inference changes?

Reviewer #2 (Comments for the Authors (Required)):

In this article, the authors consider the problem of estimating population size trajectories based on inferred identity-by-descent (IBD) segments detected between pairs of samples coming from different points in time. This temporal dimension allows them to reconstruct historic changes in population size by relating the observed number of IBD segments of a specific length to the corresponding theoretical quantities through the use of mathematical machinery introduced in previous literature on IBD segments, thereby recovering demographic features such as bottlenecks and population expansions. The authors illustrate the correctness of their method by running it on simulated data, and subsequently applying it to two different ancient DNA (aDNA) datasets, the Corded Ware (CW) dataset, and a dataset comprising samples from Britain and pertaining to the Middle Ages.

The main new feature introduced in this work is the inclusion of a time offset in the calculations which properly accounts for differences in sampling times between non-contemporaneous samples. Thus, whilst the manuscript is not groundbreaking nor does it propose any revolutionary innovations to the underlying mathematical modelling, it constitutes a valid contribution to the field by providing a method through which demographic inference for aDNA can be improved.

The manuscript is generally well-written, however in several places the authors omit details relating to the mathematical models they use and cite, and fail to provide any accompanying intuition or justification. There are some additional gaps in the methodology (for instance it is not made explicitly clear how the likelihood is computed, nor how it relates to the expected number of IBD segments of a certain length), and several quantities are not properly defined and left vague. In certain sections the manuscript would also benefit from a more thorough investigation, as certain aspects are under-explored. I strongly believe that suitably addressing the above and below raised points would greatly improve both the quality of the manuscript as well as the appeal of the proposed method.

Below please find a section-by-section breakdown of further comments:

General comments
1. In several locations (pointed exactly below) the mathematical framework lacks justification/intuition with only a reference being provided. Given that the authors do not significantly expand the mathematical framework in their work, it would be useful if they at least provided some basic intuition on the quantities they use/cite, to further the reader's understanding. Citing previous work is of course the correct thing to do, but simply listing references without much else offers little in the way of understanding and makes the results seem opaque.
2. In a number of sections throughout the manuscript I would expect the authors to have performed an extensive analysis of the various aspects that might affect the method, however this does not seem to be the case. See the ``Results" section for more details.
3. In various parts of the manuscripts the authors are slightly imprecise in the terminology and language used. It would benefit the reader's understanding and the manuscript's readability in general if this was streamlined and made consistent throughout.
4. Further simulations and testing is needed for the IBD error detection rates section. Several concepts there require reviewing or further investigation, as a number of factors affect the recall, precision and false positive rate of a given inferential procedure. I would certainly expect an exhaustive review to be conducted before one proposes simulation-based estimates of these quantities to be used within real data applications.

Introduction
1. It would be good if the authors explicitly mentioned what precisely they assume constitutes an IBD segment. Will they impose a minimum length cutoff or will segments of any length coalescing before a specific time in the past be considered IBD?

Methods
1. l.86-88 : ``...sample from n different time points and denote the set of samples in each time point t_i, ... by S_i with

sample size n_i"
- What precisely constitutes a ``sample"? What values do elements in S_i take? The general mathematical framework should be made clearer and more precise.
2. Whilst citing previous work that developed the mathematical framework used in the manuscript is the right thing to do, it should also be accompanied by an explanation or some details on the models being used, not simply deferring this to the cited literature (especially when the method proposed does not expand on nor extend said methodology). Simply explaining what assumption allows for the independence in for instance equation (1) would give some intuition, besides the fact that it should require some motivation/justification.
3. l.103-104 : ``$f(K^t_l)$ denotes the average number of uninterrupted ancestry blocks of length l at time t backward in time"
- What are ancestry blocks? Why are these not called IBD segments? Ancestry block should be formally defined if they are not IBD segments, and if they are maybe just stick to the term IBD segment.
4. Equation(2) warrants further explanation rather than just a reference - I appreciate that this is just a version of the quantity in Ringbauer et al 2017 with time changed, but precisely for this reason it should be made clearer how one arrives to this quantity, especially any assumptions required to obtain the end result. It would greatly improve the reader's understanding and intuition, without them having to dig through a string of previous work on the subject.
5. Equation (3) presumably only holds for $t > \max(t_i, t_j)$?
6. l.115-116 : ``To obtain the number of segments of length l between the two haplotypes h_1 ,h_2, we sum up $f(N_{l}^{t})$ over t from $\textnormal{max}(t_i, t_j)$ to G generations into the past"
- Isn't $f(N_l)$ in equation (4) an expectation of the number of segments rather than the number of segments? Also $f(N_l)$ isn't formally defined anywhere.
7. Equation (5) and the preceding discussion seem to suggest one can only use a continuous time approximation for the case of constant population size - one could do this for time varying Ne, say piecewise constant Ne. In this case the integrals become slightly cumbersome to write out but not too onerous to compute. It might also lead to a more apt starting Ne rather than a constant function?
8. Fig 2c, the y-axis says \# of IBD segments in each bin, but the values are all <1?
9. Fig 2c, ``For each pair of haplotypes, we took one at $t=0$ and the other at various times in the past."
- If each haplotype is associated to a sampling time ($h_1 \in S_i$, belonging to genome i which has sampling time t_i), then one would only want to consider pairs of haplotypes such that one of the two is sampled at time 0 and the other is not, rather than all possible pairs?
10. l.132-134 : ``As described in Ringbauer et al. [2017], the expected number of IBD segments whose length falls into a small length bin $[l, l + \Delta_l]$ shared between a pair of haplotypes can be approximated by $f(N_l)\Delta_l$."
- Again, this statement merits further elaboration/justification/intuition.
11. l.137 : ``Assuming that each length bin is independent"
- This is unlikely to be true - any justification/intuition?
12. The $f(N_l^t)$ notation is introduced at the start of this section, however the likelihood is presented as $\mathcal{L}(S_i, S_j|Ne)$, with no link ever being mentioned between the two, nor how the likelihood itself is calculated. This should be spelled out precisely as it is not a-priori clear how any of the likelihoods are computed.
13. l.155-156 : ``Our early exploratory experiments showed that ℓ_1 norm performed less well than ℓ_2 norm for our optimization problems. Therefore, we use quadratic penalty functions."
- Justification, intuition behind this?
14. "effectively discouraging V or Λ-shaped solutions"
- This is very imprecise terminology.
15. l.162 :``The second penalty term is the sum of the square of the first difference"
- You take the modulus, not square in eq (8)?
16. Penalty2 merits further elaboration - why the choice of Gaussian weights? This is completely mysterious, particularly given the awkward choice of normalisation.
17. l.176-178 : ``Unless otherwise stated, we fix β=250 as this term becomes negligible for the most recent part of inferred Ne and does not require fine-tuning for different scenarios."
- Why? Justification? Citations?
18. l.191-192 : ``After Ne is inferred, obtaining information about the time depth of which IBD segments are informative is helpful"
- This sentence does not make sense. Helpful for what?
19. Equations (13), (14) and (15) merit further elaboration rather than just citations.
20. Justification behind number of false positive segments being Poisson?
21. Formula for recall(l) again needs more justification and intuition!

Results
1. In the simulated data, are the ground truth simulated genotypes being used or is imputation and phasing being applied to the simulated genotypes? In case of the latter, how does this affect output? If not incorporated, the authors should analyse the effect of these two procedures on the simulated data, as they are definitely necessary to analyse aDNA data and are likely to bias the output in a non-trivial way. The authors should get a precise idea of how the induced biases affect the downstream population size estimation estimates and highlights this in their results.
2. Again in the case of simulated data, is the typical aDNA coverage mimiced somehow, and is any downsampling performed? Again this will likely impact the method's output.

3. How sensitive are the results to the choice of ancIBD parameters? Have the authors tried to vary the parameters of this method and analyse the resulting population trajectories?
4. As remarked by the authors, the IBD estimation errors used when analysing the simulated data match the true ones, so it is clear that the corrections should improve the estimation of Ne. The authors should investigate this thoroughly - are the error rates estimated indeed usable in other scenarios? A priori my guess is that they are a function of various factors (demography, coverage, etc, not least the IBD detection algorithm itself), and thus estimates on one dataset will mismatch those from another. Thus using them in any study will bias results in ways which are unclear and not understood at all. The authors should look into this, and perhaps estimate the error rates on unrelated data with different demographies and check how this impacts their inference of Ne. If this does indeed work independently of the simulated demographies/coverage/IBD detection algorithm/etc then this would prove to be a rather useful tool to account for IBD detection errors.

Discussion
1. Sentence on l. 416 does not end?

Supplementary Material
1. l.12 : Justification with regards to the choice of beta? Plots to show it doesn't affect much?
2. l.53-59 : A brief explanation of the FPR estimation should be included, not just a citation.
3. l.93-98 : ``To calculate length bias, for each simulated groundtruth IBD, we recorded inferred IBD segments that cover at least half of the groundtruth segment (therefore, there can only be one unique inferred segment satisfying this condition, if it exists at all). The length bias is the length difference between the inferred and the groundtruth segments. If no such inferred segment exists, then this ground truth segment is considered not in98 ferred, and no length bias is recorded."
- This seems arbitrary, and does not account for the segments that IBD routines call but overlap the true IBD regions marginally/not at all. Surely those segments that are called but do not correspond to a true segment should have a length bias equal to their inferred length? Otherwise $R(z|y)$ is effectively being truncated over $(0.5*y, 1.5*y)$?
4. l.116-118 : ``Similar to Supp. Note S2.2, a groundtruth IBD segment is considered to be inferred if an inferred segment covers at least half of the true IBD segment"
- This is unduly generous - why not use the proportion of genomic length (say in cM) that the inferential scheme calls as a measure of recall? Using the approach proposed in the manuscript can lead to a non-negligible inflation in the recall being reported - in the extreme case that all predicted segments overlapped the true ones by the bare minimum (i.e. 50\%), the true recall (i.e. the actual proportion detected) should be 0.5 whilst the reported recall using the author's approach ends up being 1.

Minor comments
1. The acronym IBD is used l.52 but is only introduced in l.67.
2. Ne on l.62, N_e on l.66, choose one and stick to it.
3. Citation for the choice of generation time being 29 years?
4. l.349 is a missing citation.

Reviewer #3 (Comments for the Authors (Required)):

See attached file.

Associate Editor Comments:

Review of: Estimating effective population size trajectories from time-series Identity-by-Descent (IBD) segments

Authors: Yilei Huang, Shai Carmi, and Harald Ringbauer (Huang et al.)

For publication in: Genetics

Due date: June 17, 2024

Recap of submission

The authors introduce a new pipeline called Time-Transact Ne (TTNe) to model time-transact sampling for the purpose of inferring the recent trajectories of Ne (effective population sizes). The data are identity-by-descent (IBD) segments from DNA expressed in time series. They then perform a simulation study where they analyze three different demographic settings to validate their approach. They also use TTNe for an empirical analysis.

To the best of my knowledge, the authors provide a literature review that is cognizant of the existing methodology and provide a new method to enhance the study of inheritance under different demographic scenarios. The simulation study seems to well-validate their approach for the examples provided as seen in displays like Figure 3, though I have a few technical concerns that I would like to see addressed.

Major comments

Line 195, equation (12): The Bayes' rule is applied for the probability distribution conditioned on Ne, so both lines on the right-hand side of (12) should have P(\ell|T = t, Ne) instead of P(\ell|T = t). Was it stated/proven somewhere that P(\ell|T = t) actually equals P(\ell|L = l,Ne)?

Minor comments

Lines 84-86: Each (not bold) Ne is taken to be a function over time. It may be a good idea to emphasize how time-dependence is notated here.

Line 89: Must the samples $S_1,\ldots,S_n$ be such that $t_i < t_j$ whenever $i < j$? This seems to be a conventional assumption that is affirmed in the likelihood formula in equation (6), but it might be worth it to say here that $t_1 < t_2 < \ldots < t_n$.

Note to editors/reviewers:

While investigating the issues raised by reviewer 1 on the effect of selection on the estimated Ne for the British samples, we found that the centromere region on chromosome 1 has an exceptionally high rate of IBD. We found this is due to an accidental lack of filtering IBD segments by SNP density, as recommended for high-quality IBD calls with *ancIBD* (Ringbauer et al., 2024). We have now fixed this issue and updated our results/figures/text accordingly. This error does not affect our core conclusion that the Medieval British population was steadily growing, and the general trend of Ne remains the same, while the magnitude of Ne is now about three times that of the previous estimate. We apologize for this oversight.

Associate Editor comments for the Authors:

I appreciate the exploration of various regularization strategies! Some discussion of how future work might improve on this would be helpful.

Thanks for your positive feedback! We have added a brief paragraph on regularization and potential future extensions to our discussion section.

L495 - L510:
Regularization is a challenging but necessary technique in many inverse problems for which various approaches have been developed. Prior work to infer effective population size trajectories regularize by restricting the solutions to certain classes of functions, such as functions of piecewise constant growth rates [e.g., Browning and Browning, 2015, Fournier et al., 2023]. Terhorst [2024] uses a Bayesian approach to sample from the posterior of population size histories and obtain point estimates by averaging over posterior samples. In this work, we use a penalized maximum likelihood estimation based on combining the ℓ_2 norm of the first and second derivatives, similar to Ralph and Coop [2013] and DeWitt et al. [2021]. Our specific hyperparameter selection scheme maximizes regularization strength until the regularized function causes a significant drop in goodness of fit to the observed data. This design avoids over-fitting in applications with little data by adequately increasing regularization strength. Despite this automatic choice, there is no theoretical guarantee that this hyperparameter selection scheme will avoid over- or underfitting. We hope future works will systematically explore various regularization approaches against simulated data and compare their relative advantages and weaknesses.

Also, I'm surprised that you can find so much very long IBD among these ancient samples - although I probably should not be, given the smaller sample sizes. This is a good thing, because shorter IBD would be substantially noisier. I think this is a neat and interesting result that could be highlighted in the abstract or Introduction.

We added a sentence at the end of the Introduction to highlight this observation:

L80 - L83:

Notably, the inferred effective population size trajectory of the British Isles is an order of magnitude larger than that of Corded Ware, demonstrating IBD sharing is a useful signal even in large populations given sufficient sample size (Fig.6).

By my reading (apologies if I've missed it), there is not sufficient description of what (in particular: how many) samples were used in each of the empirical applications. Sample sizes should be given in the main text, and a table of sample identifiers and estimated dates should be provided in the Supplement.

We now explicitly indicate the sample sizes in Figure 5&6. The sample sizes for the two empirical applications are also mentioned now in the corresponding paragraphs in the main text.

Equation (1): Here the function f( ) on the two sides of the equation mean different things. If taken literally, equation (1) suggests that there is a single function f( ) that is evaluated with two different arguments, N_l^t and K_l^t, on the two sides of the equation. A more usual way to denote what the authors want to say here would be to define, say, f_N(l,t) to be the expected number of segments of length l from time t ago, and similarly f_K(l, t). (I see that maybe the authors are following the notation of Ringbauer et al but replacing \mathbb{E}[ ] with f( ); however, for this to make sense one needs to at minimum define the random variables N_l^t and K_l^t.)

Equation (1): Units should be more precisely specified for both time and length: is this a density?

We now explicitly specify that f is a “density” similar to the probability density function, although it is not a probability measure because it does not integrate to 1.

L119 - L122:

Throughout, functions denoted by $f(\cdot)$ are densities, i.e., the quantity they describe (usually the expected numbers of IBD segments) is obtained by integrating the density over an interval (e.g., over IBD length and time interval). Notably, it is not a probability measure because it does not integrate to 1.

Equation (1): the factor from K is not "the average number of uninterrupted ancestry blocks", although it is related to that.

We have changed our notation (now using f_K and f_N instead of f) and explicitly state that $f(\cdot)$ denotes a density (see answer above). Moreover, we clarified the definition of K::

L114 - L116:

where the first factor $f_K(l,t)$ denotes the expected number of chromosome segments of length l after the chromosome has experienced $2t$ meioses.

Above equation (2): the text says "\Delta t" but the inline equation has only \Delta.

Thank you for spotting this typo. We have corrected it.

Equation (2): If looking for additional justification for this modification to Ringbauer, you can cite Ralph & Coop, which is phrased more generally than Ringbauer (to obtain this equation, differentiate equation (6) in Ralph & Coop with respect to x).

Thank you for this suggestion. We have added this as an alternative derivation in our method description:

L144 - L148:
We note that Eq. 2 can also be derived from Eq.6 in Ralph and Coop [2013], which gives the mean number of pieces of length at least x obtained by cutting the chromosome at the recombination sites of $n = t^*$ meioses. It is related to $f_K(l,t)$ by integrating $f_K(l,t)$ from l to the length of the chromosome (aka IBD over the entire chromosome). Differentiating Eq.6 from Ralph and Coop [2013] with respect to l (and flipping the sign) yields Eq. 2.

Equation (9): Script N is not defined.

We now state that $\mathcal{N}$ denotes a Gaussian probability density:

L209:
and $\mathcal{N}(\mu, \sigma)$ denotes a standard Gaussian density with mean μ and standard deviation σ.

l.185: The expression that involves both a numpy expression in Python and
an undefined script N denoting a probability density should be
written out clearly.

We now use standard math symbols.

Several places: "principal component analysis" not "principle component analysis"

Thank you for spotting these typos. We have corrected them.

Reviewer #1 (Comments for the Authors (Required)):

Summary:
This manuscript describes a new method, TTNe, for inferring population size history from IBD distribution of time-stratified genomic sequences. They derived the expected IBD distributions across time under a given Ne(t), then use the likelihood function (with penalty terms) to estimate optimal \hat{Ne}(t) with a SciPy's built-in optimization algorithm. They showed with coalescent simulations that their framework can reliably predict IBD distributions, and the subsequent Ne(t) inference are also satisfactory. The authors then applied TTNe on human aDNA data, producing inferences consistent with previous studies.

Major comments:
- Although the authors later considered admixture in empirical analyses and further discussed it in Discussion, it's not addressed in simulation studies. Even though all methods for inferring pop size history are inevitably affected by violations of assuming the population to be panmictic, it would still be nice to see how susceptible they are compared with each other.

Admixture will have relatively small effects for many applications that use long IBD segments in aDNA unless the admixture happens temporally close to the samples. Because we use IBD segments longer than 8 cm, older demographic events and admixtures are outside the time depth over which most long IBD segments coalesce. To support this intuitive argument, we simulated the demography of a prominent Bronze Age European group (aka Corded Ware) as an admixture between Yamnaya and Neolithic farmers. We provide the full details about simulation and interpretations in Supplementary Note 5. In short, we found that our method can accurately infer the effective population size for the simulated Corded Ware demography for the time period post-dating the admixture event.

- When it comes to temporal genetic data, one issue that frequently comes up is the sampling scheme design. Although it is relatively non-negotiable for aDNA datasets, geneticists studying experimental evolution would greatly benefit from advice on (even just rules-of-thumb) appropriate sampling intervals and sample sizes. I noticed that the authors chose different sampling times for the 3 demographic models in the simulation, were they just arbitrarily decided or is there any justification for choosing these time points? I see that the authors have explored further on this topic in Supp. Note S2.3, where (in my humble opinion) the main findings in this section should be mentioned in the main text.

We chose different sampling times for the three demographic models based on the time of the demographic event (e.g., around the timing of the bottleneck and at the start of the exponential growth) to demonstrate the advantage of including time-series samples for inferring Ne.

Based on simulation results, we now discuss the sampling scheme in the Discussion section:

L511 - L520:

> To apply TTNe to empirical aDNA studies, the temporal distribution of samples has important implications for inference. While sampling is often limited by aDNA preservation and availability of human osteological remains, our simulations can guide selecting samples in empirical studies targeting specific demographic events. Unsurprisingly, sampling closer to the demographic event of interest increases resolution. In Fig.S23, for example, we found that having an additional set of samples aside from samples at $t = 0$ improves the inference of the strength and timing of a bottleneck. In Fig.S29, we compared two sampling strategies where, in one case,

two sets of samples are taken ten generations apart centered around the bottleneck time, and in the other case, 20 generations apart. We found the former sampling scheme performs better.

- I also wonder how much the DNA decays in aDNA datasets affect downstream inferences. I see that it was later discussed in Supp. Note S2.2, though I think this issue is worth addressing in the main text.

Genotyping errors in aDNA datasets are indeed comparably high and a common concern. For our method to infer Ne, DNA data quality affects inference via its influence on the accuracy of IBD segment calling. This potential bias is addressed directly by our modeling of IBD calling error, which we estimated by simulating aDNA data matching the average coverage of our empirical samples (see Supplementary Note 2 and Fig S3). Fig. S3 shows the power and IBD length bias for different coverages. We depict the false positive rate for different coverages in Fig.S6.

We did not explicitly test the effects of aDNA damage on IBD calling quality because Marques et al. had already benchmarked the effects of aDNA damage on the accuracy of imputed genotypes (which are the input data for our IBD calling) and had found no strong positive correlation between imputation error rate and damage rate (Fig.2C). With read trimming (as commonly applied in aDNA analysis), aDNA samples' remaining average error rate is only 1-2% higher than samples with no damage at all. Therefore, given that we used aDNA samples with similar damage rates as the empirical Corded Ware and Medieval Britain samples for simulations used to estimate our error model, aDNA damage does not introduce substantial bias in our empirical results.

However, we stress that to obtain unbiased results in empirical studies, one must estimate the IBD error model from samples whose imputation accuracy is similar to that of the empirical samples. We also performed simulations to investigate the effect of misspecified error model on inferred Ne. We concluded that the level of misspecification in our empirical data is unlikely to bias our inferred Ne (see details in Supplementary Note S4). We added several sentences clarifying this:

L330 - L338:
To estimate error models for IBD detection, we first computed the average coverage of 1240k and WGS data in the empirical samples and then simulated IBD (described in detail in Supplementary Note 2) by downsampling 1240k/WGS BAM files to the average coverage. We then used the average of the estimated 1240k and WGS error models for the empirical data analysis. Given that our estimated Ne has the same order of magnitude as Ne estimated from linkage disequilibrium (LD) using hapNe-LD [Fournier et al., 2023] (Fig.S29, Fig.S34) and the observation that the inferred Ne trajectory is robust to modest misspecification of the error model, we believe that our approach is not substantially biased.

- It might be less of a concern from a theoretical/modeling perspective, but I'm curious about the relative computational performance of TTNe (say, compared with HapNe-IBD).

Please see Fig.S30 for a runtime comparison with hapNe-IBD. We added a short note about runtime in our main text:

L239 - L245:
Because our method calculates the likelihood for all pairs of sets of samples, the runtime scales quadratically with the number of sample sets. The optimization step of TTNe is slower than that of hapNe-IBD because the solution of TTNe is less constrained than that of hapNe-IBD, hence the iterative

optimization generally takes more iterations to converge. However, the runtime remains manageable for modern high-performance CPUs (Fig. S30, e.g., the two main applications described in this manuscript finished within a few CPU-hours).

Minor comments:
- Line 110: typo? $2t - \Delta$ is missing a t

Thank you for spotting this typo; we have corrected it. For conciseness, we now also abbreviate the recurring term $2t - \Delta t$ as t*.

- Fig 2c: it'd also help to show how the framework aligns with simulation to plot the $\Delta_I(t)$ as shades around the theoretical lines

We are not sure what the reviewer meant by $\Delta_I(t)$. If it refers to the confidence interval of # of IBD segments in the simulated data, we now include this in Supp Fig. S16. After adding the error bars, the plot becomes very crowded, so we chose not to use this version in the main figure.

- Eq. 9: is there a particular reason for $\sigma = T_{max}/3$? Why?

We added a few sentences explaining the intuition and conducted experiments to show the effects of this particular choice:

L210 - L212:
We chose $\sigma = T_{max}/3$ because 99.7% of the probability mass of Gaussian distribution lies within 3σ from its mean. Therefore, w_t is nearly zero for t close to zero while it is close to one for t close to T_{max}. Qualitatively, this term penalizes changes in $\mathbf{N_e}$ deeper in time where signals from IBD diminish. Our simulations confirm that this term helps stabilize the N_e estimate at deeper time depths (Fig.S22)

- Line 283--5: why aren't the sampling times identical across all three demographic models? How does the choice of sampling time affect the inference?

The choices of sampling times vary across the three demographic models because the timing of the demographic event differs among them. We wanted to demonstrate that having samples closer to the demographic event of interest is beneficial for the accurate estimation of Ne. This observation has implications for the design of the genetic study: if researchers have expectations on the timing of a demographic event they want to investigate, they should prioritize genotyping samples from periods close to the event. For example, the bottleneck occurs at t=30 in the bottleneck demography, so we included two sampling times (t=25 and t=35) around t=30. Similarly, for the exponential demography, the onset of population growth happens at t=50, so we included two sampling times (t=15 and t=30) that post-dated the expansion. The constant demography is trivial, and we have chosen three somewhat arbitrary sampling times (t=0, t=15, t=45).

- Line 329: the latter half of this sentence reads very confusing to me. Is the "> 12cM" supposed to be removed?

We meant that we sum the lengths of all IBD segments longer than 12cM. If this sum is over 100cM, it indicates a very close genealogical relationship. We excluded such pairs of genomes that share

exceptionally many IBD segments from our analysis. We updated our wording to clarify this filtering:

L402 - L405:
We only kept one individual with higher coverage from pairs of closely related individuals, here defined as having at least three IBD segments longer than 12cM or the summed length of all IBD segments longer than 12cM exceeding a total of 100cM.

- Line 349: missing citation

Thank you for spotting this. We have fixed this error.

- Line 393: the en-dash ("--") should be em-dash ("---") here

Thank you for spotting this typo. We have fixed this.

- Line 417: typo?

Thank you for spotting this typo. We have fixed this.

- About how to address selection from black death affect the inference: have the authors tried excluding certain well-known immune-related genomic areas (e.g., HLA complex, and/or reading frames of all immune-related genes?), and see how much the Ne(t) inference changes?

Thank you for the valuable suggestion. Masking all reading frames of immune-related genes will make chromosomes too fragmentary for our analysis using long IBD segments. Therefore, we chose to exclude regions showing signals of selection as listed in Browning et al. They detected IBD segments in the UK Biobank for the white British subset of individuals and identified regions whose IBD sharing rate was four standard deviations higher than the genome-wide average as potential targets for natural selection. We show their Table 1 below for ease of reference. The regions contain several well-known examples, such as the MHC and LCT loci and genes related to pigmentation.

Table 1. Regions of Highest IBD Rate in UK Biobank White British Analysis

Chromosome	Peak position (Mb)	Region (Mb)[a]	Max IBD %	Notes[b]
2	135.78	135.05–138.42 (2q21-22)	0.1702	*LCT* (136.55–136.59) is known to be subject to recent positive selection[48]
3	47.60	45.18–53.19 (3p21)	0.0264	chemokine receptor genes including *CCR9* (45.93–45.94) and *CXCR6* (45.98–45.99); this region shows adaptive introgression from Neanderthals[49]
3	123.44	122.36–124.69 (3q21)	0.0251	*ADCY5* (123.00–123.17) is under long-term balancing selection[50]
4	38.84	38.25–40.18 (4p14)	0.0270	Toll-like receptor genes including *TLR1*, *TLR6*, and *TLR10* (38.77–38.86); this locus is known to be under positive selection[51]
5	33.89	32.66–34.57 (5p13)	0.0262	*SLC45A2* (33.94–33.98, previously known as *MATP*) is a pigmentation gene that has undergone recent positive selection in Europe[52]
6	24.87	24.01–28.96	0.0267	MHC locus containing *HLA* genes (28.48–33.45) is known to be subject to strong selection[53]
	33.96	32.70–36.27 (6p21-22)	0.0298	
6	106.33	105.97–107.31 (6q21)	0.0271	*PRDM1* (106.53–106.56); this locus shows a signal of recent selection[54]
12	53.59	52.62–54.60 (12q13)	0.0260	Type II Keratins (*KRT1-8*; 52.63–53.32); this locus has experienced adaptive introgression from Neanderthals[55]
12	113.46	111.64–114.11 (12q24)	0.0316	OAS locus (*OAS*1-3) (113.34–113.45); this locus has experienced adaptive introgression from Neanderthals[56]
15	31.59	30.76–32.58 (15q13)	0.0542	*TRPM1* (31.29–31.45), a pigmentation gene under selection in non-Africans[57]
17	44.75	42.17–45.88 (17q21)	0.0274	17q21.31 *MAPT* inversion (43.44–44.85);[c] H2 form has been positively selected in Europe[58]
22	22.56	21.68–22.99 (22q11)	0.0267	*UBE2L3* (21.90–21.98) is associated with multiple auto-immune diseases[59]

We then examined whether our Medieval British data show evidence of excessive IBD sharing in the these regions, indicated by gray boxes. The sample size is relatively small, so the estimated IBD rates per genomic region are noisy. However, the listed regions do not seem to have excessive IBD sharing

compared with the rest of the genome.

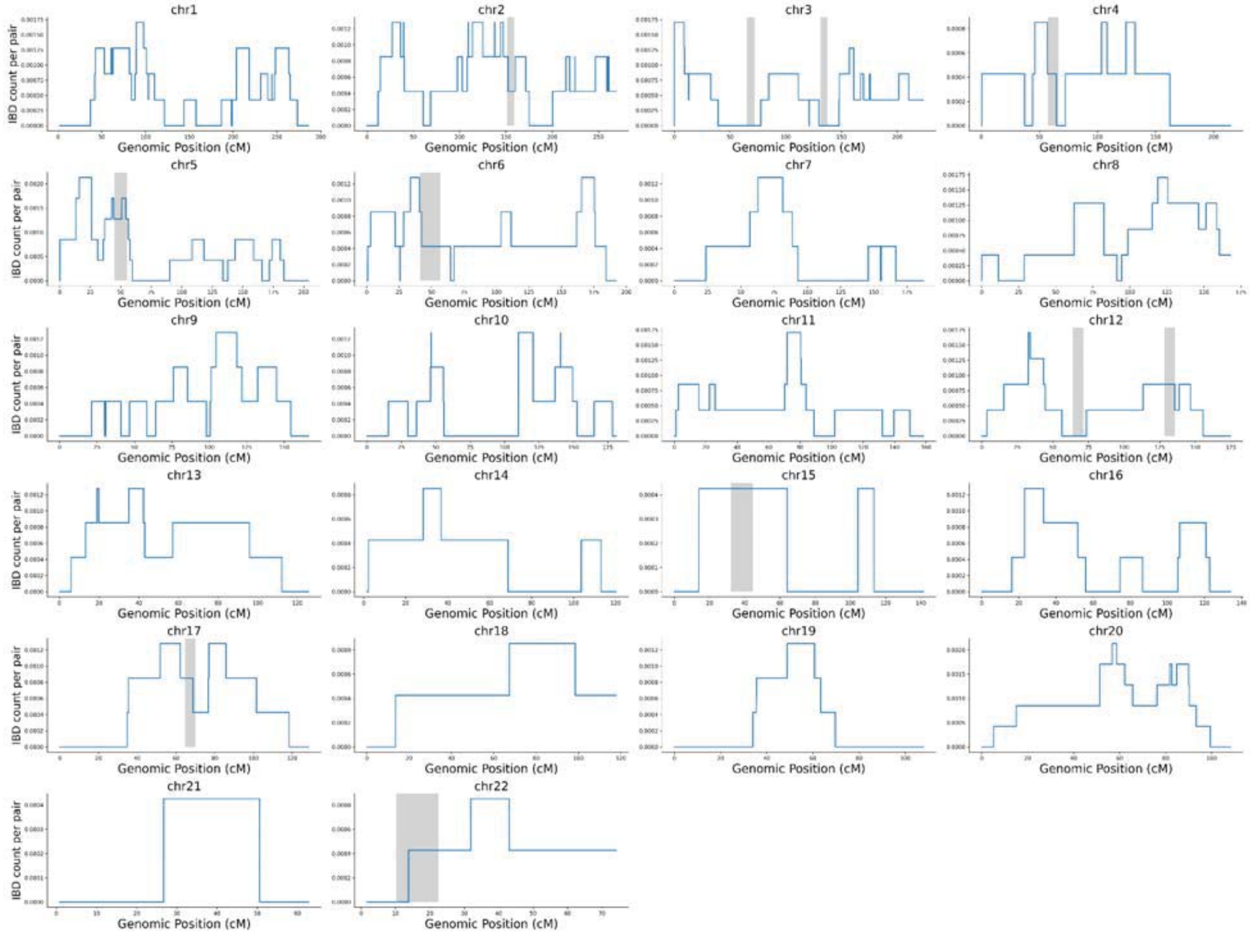

To confirm that the regions under selection do not qualitatively impact the population size inference, we masked IBD segments in these regions and re-ran TTNe. Indeed, the resulting Ne trajectory is nearly identical to that without masking (see Figure below). We now report the results from the masked genome in the main text.

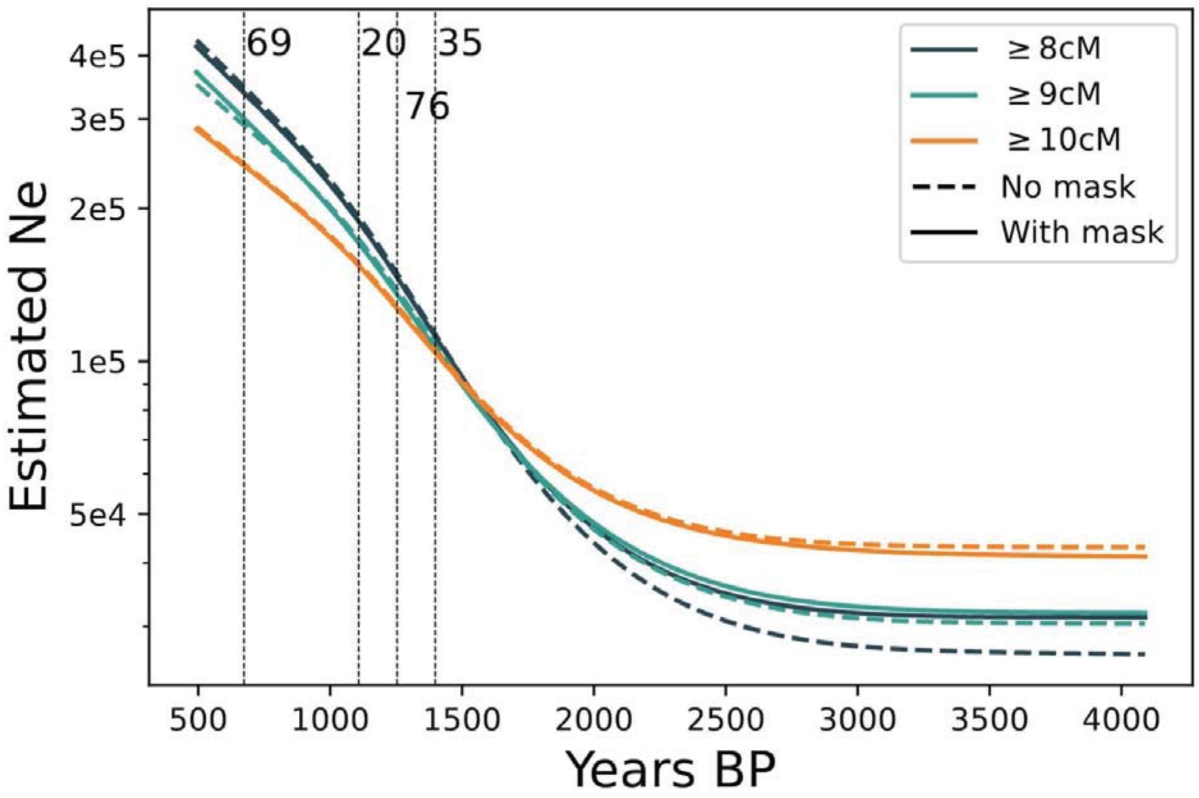

69
20
35
76
≥ 8cM
≥ 9cM
≥ 10cM
No mask
With mask
4e5
3e5
2e5
1e5
5e4
Estimated Ne
500
1000
1500
2000
2500
3000
3500
4000
Years BP

Reviewer #2 (Comments for the Authors (Required)):

In this article, the authors consider the problem of estimating population size trajectories based on inferred identity-by-descent (IBD) segments detected between pairs of samples coming from different points in time. This temporal dimension allows them to reconstruct historic changes in population size by relating the observed number of IBD segments of a specific length to the corresponding theoretical quantities through the use of mathematical machinery introduced in previous literature on IBD segments, thereby recovering demographic features such as bottlenecks and population expansions. The authors illustrate the correctness of their method by running it on simulated data, and subsequently applying it to two different ancient DNA (aDNA) datasets, the Corded Ware (CW) dataset, and a dataset comprising samples from Britain and pertaining to the Middle Ages.

The main new feature introduced in this work is the inclusion of a time offset in the calculations which properly accounts for differences in sampling times between non-contemporaneous samples. Thus, whilst the manuscript is not groundbreaking nor does it propose any revolutionary innovations to the underlying mathematical modelling, it constitutes a valid contribution to the field by providing a method through which demographic inference for aDNA can be improved.

The manuscript is generally well-written, however in several places the authors omit details relating to the mathematical models they use and cite, and fail to provide any accompanying intuition or justification. There are some additional gaps in the methodology (for instance it is not made explicitly clear how the likelihood is computed, nor how it relates to the expected number of IBD segments of a certain length), and several quantities are not properly defined and left vague. In certain sections the manuscript would also benefit from a more thorough investigation, as certain aspects are under-explored. I strongly believe that suitably addressing the above and below raised points would greatly improve both the quality of the manuscript as well as the appeal of the proposed method.

Below please find a section-by-section breakdown of further comments:

General comments
G1. In several locations (pointed exactly below) the mathematical framework lacks justification/intuition with only a reference being provided. Given that the authors do not significantly expand the mathematical framework in their work, it would be useful if they at least provided some basic intuition on the quantities they use/cite, to further the reader's understanding. Citing previous work is of course the correct thing to do, but simply listing references without much else offers little in the way of understanding and makes the results seem opaque.

Thank you for this constructive suggestion. Upon reflection, we agree that referring to and citing previous theoretical results without providing some intuition creates a sense of opaqueness. To avoid frustrating the reader, we have expanded our derivations and attempted to add intuitive descriptions to our formulas.

G2. In a number of sections throughout the manuscript I would expect the authors to have performed an extensive analysis of the various aspects that might affect the method, however this does not seem to be the case. See the ``Results" section for more details.

G3. In various parts of the manuscripts the authors are slightly imprecise in the terminology and language used. It would benefit the reader's understanding and the manuscript's readability in general if this was streamlined and made consistent throughout.

G4. Further simulations and testing is needed for the IBD error detection rates section. Several concepts there require reviewing or further investigation, as a number of factors affect the recall, precision and false positive rate of a given inferential procedure. I would certainly expect an exhaustive review to be conducted before one proposes simulation-based estimates of these quantities to be used within real data applications.

Thanks for this constructive feedback. We have conducted additional experiments to test the robustness of our error rate modeling. See our response to the specific points below.

Introduction

I1. It would be good if the authors explicitly mentioned what precisely they assume constitutes an IBD segment. Will they impose a minimum length cutoff or will segments of any length coalescing before a specific time in the past be considered IBD?

We now explicitly state in the introduction that we use IBD segments longer than a threshold of several centimorgan. This cutoff is motivated by the fact that shorter IBD segments cannot be reliably detected by existing methods. In most human populations, segments of length >8 cM almost exclusively originate from a single co-ancestry event (see Chiang et al).

L67 - L69:

Here, we present TTNe, a method to estimate recent effective population size Ne trajectories from time-series aDNA data based on medium-to-long IBD segments, typically >8 centimorgan (cM).

Methods

M1. l.86-88 : ``...sample from n different time points and denote the set of samples in each time point t_i, ... by S_i with sample size n_i"

- What precisely constitutes a ``sample"? What values do elements in S_i take? The general mathematical framework should be made clearer and more precise.

A sample here refers to an ancient genome sequence available for analysis. Each such sample, or ancient genome, is associated with a date when the person carrying the genome has lived. We have added an explanation:

L91 - L92:

We assume that we have samples (i.e., individual genomes) from people who have lived in n different time points.

M2. Whilst citing previous work that developed the mathematical framework used in the manuscript is the right thing to do, it should also be accompanied by an explanation or some details on the models being used, not simply deferring this to the cited literature (especially when the method proposed does not expand on nor extend said methodology). Simply explaining what assumption allows for the

independence in for instance equation (1) would give some intuition, besides the fact that it should require some motivation/justification.

We have added an explanation of why the left-hand-side equation is the product of the two terms on the right-hand side:
L122 - L124:
Eq.1 holds because the number of IBD segments coming from generation t is the number of chromosome pieces multiplied by the probability of coalescence.

M3. l.103-104 : ``$f(K^t_l)$ denotes the average number of uninterrupted ancestry blocks of length l at time t backward in time"
- What are ancestry blocks? Why are these not called IBD segments? Ancestry block should be formally defined if they are not IBD segments, and if they are maybe just stick to the term IBD segment.

They are not IBD segments. We now explicitly state what K means and we changed the notation from $f(K_l^t)$ to $f_K(l,t)$.

L114 - L117:
where the first factor $f_K(l,t)$ denotes the expectation of the number of chromosome segments of length l after the chromosome has experienced 2t meioses (where each recombination event starts a new segment). The factor 2 appears because tracing two lineages back to the common ancestor t generations ago leads to a total branch length of 2t.

M4. Equation(2) warrants further explanation rather than just a reference - I appreciate that this is just a version of the quantity in Ringbauer et al 2017 with time changed, but precisely for this reason it should be made clearer how one arrives to this quantity, especially any assumptions required to obtain the end result. It would greatly improve the reader's understanding and intuition, without them having to dig through a string of previous work on the subject.

We now expanded our explanation of Eq. 2, detailing where each part of the equation comes from. We hope this aids readers' understanding.

$$f_K(l,t) = \underbrace{2t^* e^{-t^* l}}_{i} + \underbrace{(L-l)(t^*)^2 e^{-t^* l}}_{ii}. \qquad (2)$$

L132 - L148:
Part (i) describes IBD segments ending in one of the two ends of the chromosome, while part (ii) describes IBD segments fully in the interior of the chromosome. For part (i), because the total branch length up to the most recent common ancestor is t^*, after fixing one border of the IBD segment to the chromosome end, the distance to the other border follows an exponential distribution with rate t^*.

Therefore, the probability of having a segment of length l at either of the chromosome ends is $t^* e^{-t*l}$. For the segments in the interior of a chromosome, we note that recombination happens with probability $t * \Delta L$ within a small interval ΔL. Once a recombination happens, the waiting time until the next recombination happens again follows an exponential distribution with rate t^*. Therefore, the probability of obtaining a segment of length l that begins from a small interval ΔL is $t^* e^{-t*l} t^* \Delta L$. Adding up the terms from all possible starting intervals of a segment of length l (which sum up to a total length $L - l$) results in part (ii) in $f_K(l, t)$.

We note that Eq.2 directly follows from Eq.6 in Ralph et al., 2013. Eq.6 in Ralph et al., 2013 gives the mean number of pieces of length at least x obtained by cutting the chromosome at the recombination sites of $n = t^*$ meioses. It is related to $f_K(l, t)$ by integrating $f_K(l, t)$ from l to the length of the chromosome (aka IBD over the entire chromosome). Differentiating Eq.6 from Ralph et al., 2013 with respect to l (and flipping the sign) yields Eq.2.

M5. Equation (3) presumably only holds for $t > \max(t_i, t_j)$?

We have added this natural condition in Eq.3.

M6. l.115-116 : ``To obtain the number of segments of length l between the two haplotypes h_1 ,h_2, we sum up $f(N_{l}^{t})$ over t from $\textnormal{max}(t_i, t_j)$ to G generations into the past"
- Isn't $f(N_l)$ in equation (4) an expectation of the number of segments rather than the number of segments? Also $f(N_l)$ isn't formally defined anywhere.

We now explicitly state that this is an expectation and formally define $f_N(l)$:
L151 - L152: To obtain $f_N(l)$, the expected number of IBD segments of length l between a pair of haplotypes h_1, h_2, we sum up $f_N(l, t)$ over t from $\max(t_i, t_j)$ to G generations into the past:

M7. Equation (5) and the preceding discussion seem to suggest one can only use a continuous time approximation for the case of constant population size - one could do this for time varying Ne, say piecewise constant Ne. In this case the integrals become slightly cumbersome to write out but not too onerous to compute. It might also lead to a more apt starting Ne rather than a constant function?

We updated our writing to reflect that other specific cases may also have analytic solutions:

L154 - L155:
“For some demographic scenarios, inserting a continuous-time approximation of Eq. 3 into Eq. 4 and then performing integration can yield closed-form results. For instance, for a constant population size Ne…… ”

Your suggestion of a piece-wise fit via analytic expressions is a promising alternative. However, obtaining a starting condition from a piece-wise fit is already a high-dimensional optimization, especially given that one should consider how to choose the boundary of each piece. Methods such as hapNe-IBD use the piecewise fitting as their main inference engine, but “the lengths of the time intervals are iteratively tuned so that each time interval contains the same number of expected ancestors of IBD segments”, which is itself quite involved for a simple task of setting the initial values for the optimization step. To allow the optimization to thoroughly explore the solution space, as described in Eq.13 and L234 - L235, we add small Gaussian perturbations to the constant Ne, which we have found to perform well in simulations.

M8. Fig 2c, the y-axis says \# of IBD segments in each bin, but the values are all <1?

Thanks for pointing this out. We meant to say the y-axis is the average number of segments shared between per pair of haplotypes - a value between 0 and 1 indeed. But the y label was not correctly stated. We have updated it.

M9. Fig 2c, ``For each pair of haplotypes, we took one at $t=0$ and the other at various times in the past."
- If each haplotype is associated to a sampling time ($h_1 \in S_i$, belonging to genome i which has sampling time t_i), then one would only want to consider pairs of haplotypes such that one of the two is sampled at time 0 and the other is not, rather than all possible pairs?

We attempted to clarify the legend of Fig.2 as follows:

To test the correctness of Eq.4, in each simulated replicate, we took pairs of haplotypes so that one is sampled at t=0 and the other is sampled at one of t=15,25,35,45 (so that Δt=15,25,35,45).

W stress that we use all possible pairs for Ne inference, i.e., when calculating the log-likelihood of our model (see Eq.7 in main text). In other words, for inference, we are not using only "pairs of haplotypes such that one of the two is sampled at time 0 and the other is not". It is only the case in Fig.2, where the goal is to use some examples to demonstrate the validity of the mathematical modeling.

M10. l.132-134: ``As described in Ringbauer et al. [2017], the expected number of IBD segments whose length falls into a small length bin $[l, l + \Delta_l]$ shared between a pair of haplotypes can be approximated by $f(N_l)\Delta_l$."
- Again, this statement merits further elaboration/justification/intuition.

We clarify this point in L119 - L122:

"Throughout, functions denoted by $f(\cdot)$ are densities, i.e., the quantity they describe (usually the expected numbers of IBD segments) is obtained by integrating the density over an interval (e.g., over a length and time interval)."

Integrating a function f over $[l, l + \Delta_l]$ can be approximated by $f(l)\Delta_l$ for small Δ_l.

M11. l.137 : ``Assuming that each length bin is independent"
- This is unlikely to be true - any justification/intuition?

We agree that different IBD length bins do not contain strictly independent observations. However, it is a reasonable approximation for the following reasons. Once we remove closely related pairs from the analysis (similarly to other methods), most pairs of individuals share at most one long IBD segment (>8cM) on any given chromosome. Further, the genealogies of different chromosomes are approximately independent, given no physical linkage between chromosomes. While different chromosomes still share the population pedigree and are thus not entirely independent (King et al. 2018), these effects are weak beyond very few generations.

Another possible source of dependency is that each genome is involved in multiple pairs (n-1 pairs, for a total sample size n). Therefore, IBD segments from different pairs of samples are not fully independent. However, theory shows this dependence is relatively weak (Carmi et al. 2013). Therefore, it is reasonable to assume that IBD segment counts are independent when looking at different subsets of segments of different lengths.

Finally, the fact that our method can unbiasedly infer effective population size, as shown by simulations in our article, confirms that assuming independence does not substantially bias the estimates.

M12. The $f(N_l^t)$ notation is introduced at the start of this section, however the likelihood is presented as $\mathcal{L}(S_i, S_j|Ne)$, with no link ever being mentioned between the two, nor how the likelihood itself is calculated. This should be spelled out precisely as it is not a-priori clear how any of the likelihoods are computed.

We now write this out explicitly in Eq.6.

13. l.155-156 : ``Our early exploratory experiments showed that ℓ_1 norm performed less well than ℓ_2 norm for our optimization problems. Therefore, we use quadratic penalty functions."
- Justification, intuition behind this?

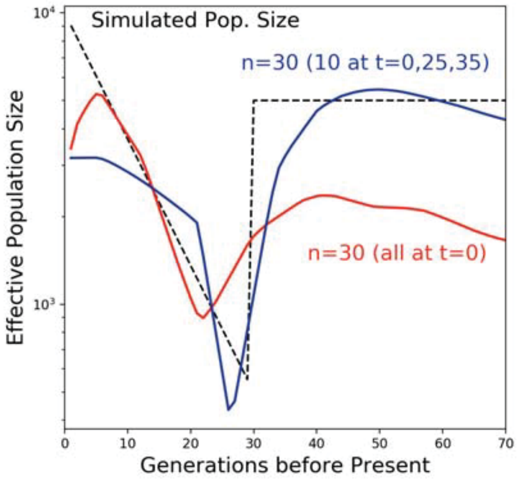

The figure above shows the inferred Ne using the l_1 norm with three sampling point (blue) and one sampling point (red). One can see that in both cases, the inferred Ne curve is discontinuous at various places. This is because using l_1 norm on the kth-order time derivative encourages piecewise (k-1)^th-order polynomial solutions (see for example DeWitt et al 2021., Tibshirani 2013, Kim et al., 2019). This means that when we use l_1 norm on the second derivative of Ne with respect to time, we encourage piecewise linear solutions. However, the points of discontinuity in the piecewise linear solution have less historical and demographic plausibility and may also convey a false sense of certainty about the timing of demographic events. For example, for the blue line, readers not well-versed in the property of l_1 regularization may read the sharp discontinuity at t $\sim$ 26 to be the exact timing of the bottleneck. As shown in the figures presented throughout our manuscript, l_2 regularization tends to smooth drastic changes in Ne, which is more plausible.

M14. "effectively discouraging V or Λ-shaped solutions"
- This is very imprecise terminology.

We now instead state that this penalty term “discourages solutions with large curvature” (Line 204).

M15. l.162 :``The second penalty term is the sum of the square of the first difference"
- You take the modulus, not square in eq (8)?

Thanks for pointing this out. We have fixed the typo in Eq.8

M16. Penalty2 merits further elaboration - why the choice of Gaussian weights? This is completely mysterious, particularly given the awkward choice of normalization.

First, we note that there are many possible weights to choose from — many of them will have similar effects as the Gaussian weights. We chose Gaussian weights simply because Gaussian distributions are widely used, do not need a detailed introduction, and nearly every programming language efficiently implements them. We now add a few sentences explaining why we chose this particular form:

L210 - L213:
We chose $\sigma = T_{max}/3$ because 99.7% of the probability mass of Gaussian distribution lies within 3σ from its mean. Therefore, w_t is nearly zero for t close to zero while it is close to one for t close to T_{max}. Qualitatively, this term penalizes changes in Ne deeper in time where signals from IBD diminish.

We note that there could have been alternative ways to achieve the desired regularization effect. We now include a figure (Fig.S22) comparing the estimated Ne with and without the Gaussian weights, showing that our choice of regularization achieves the desired effect of strong regularization at deep times and weak regularization at recent times. In particular, without this regularization, the Ne in the recent past is strongly biased for the bottleneck and exponential growth model.

M17. l.176-178 : ``Unless otherwise stated, we fix β=250 as this term becomes negligible for the most recent part of inferred Ne and does not require fine-tuning for different scenarios."
- Why? Justification? Citations?

We now include Fig. S21 to show how varying β effects the estimated Ne, which we refer to in the main text. In the figure caption of Fig. S21, we added the following explanation:

Because of the decaying weights we employed (Eq. 9 and Eq. 10 in the main text), the choice of β has negligible effects on the recent past, where most of the IBD signal comes from. We note that $\beta = 250$ or $\beta = 2500$ yield similar results, but too small values of β (e.g., 0 or 25) cause the estimated Ne to be unstable at deeper time depths. Therefore, we fix $\beta = 250$, because this value is big enough to guard against unstable Ne at deeper time depth, where one has essentially no signal from long IBD segments.

M18. l.191-192 : ``After Ne is inferred, obtaining information about the time depth of which IBD segments are informative is helpful"
- This sentence does not make sense. Helpful for what?

As we wrote in the main text,

L351 - L354:
“At deeper time depth, HapNe-IBD estimated Ne tends to converge to the average Ne over time, while TTNe tends to converge to the estimated Ne values in the more recent time. These different behaviors mainly stem from different choices of regularization.”

As one goes deeper in time, there are very few coalescences of long IBD segments. Therefore, regularization is the dominant factor influencing the estimated Ne in this deeper time regime instead of any actual population genetic signal. We think that calculating the posterior distribution of TMRCA for IBD segments helps users to better understand which time period in the inferred Ne starts to be based primarily upon regularization and not actual signal.

M19. Equations (13), (14) and (15) merit further elaboration rather than just citations.

Eq.13 (in the revised manuscript, now Eq.14) is a standard application of the Bayes theorem. For Eq.14 (now Eq.15) and Eq.15 (now Eq.16), we have added explanations, quoted below for convenience:

L251 - L261:
where $P(l|T=t)$ denotes the probability density of having an IBD segment of length l spanning a chosen marker and originating from t generations ago, which is given by Palamara et al. [2012], Baharian et al. [2016]:

$$P(l|T=t) = (2t)^2 l e^{-2tl}$$

The above density is Erlang-2, which is arrived at given that the distance to the next recombination on each side of the focal marker is exponential with rate 2t (the total branch length to the common ancestor). We note that conditioning on a fixed time t to the most recent common ancestor, the IBD length distribution is independent of $\mathbf{N_e}$, as shown in Palamara et al. [2012]. When Δt generations separate two samples from different generations, this term becomes

$$P(l|T=t) = (2t-\Delta t)^2 l e^{-l(2t-\Delta t)}$$

where t is measured from the younger sample.

M20. Justification behind number of false positive segments being Poisson?

A Poisson variable describes the number of randomly and rarely occurring events. Hence, it is the natural choice for modeling the number of false positive segments.

M21. Formula for recall(l) again needs more justification and intuition!

We assume this refers to the recall formula in Fig. 4b. The functional form of this formula is based on Ralph & Coop 2013. We modified some of its constants so that its value is about 90% at IBD of length 8cM. We suppose that Ralph & Coop have designed it to accurately fit their empirically estimated recall curve. The particular functional form has no interpretation beyond that.

For our purposes, we only use this formula for simulation. While there are many other possible choices, the point is that even when IBD detection errors are abundant, given the correct error model (or a reasonable approximation of it), we can accurately recover Ne.

We stress that the recall is estimated numerically in empirical applications. Therefore, our empirical data analysis does not use this particular recall formula.

Results

R1. In the simulated data, are the ground truth simulated genotypes being used or is imputation and phasing being applied to the simulated genotypes?

In case of the latter, how does this affect output? If not incorporated, the authors should analyse the effect of these two procedures on the simulated data, as they are definitely necessary to analyse aDNA data and are likely to bias the output in a non-trivial way. The authors should get a precise idea of how the induced biases affect the downstream population size estimation estimates and highlights this in their results.

R2. Again in the case of simulated data, is the typical aDNA coverage mimiced somehow, and is any downsampling performed? Again this will likely impact the method's output.

Reply to R1 and R2:

Please refer to Section 1.6 in the main text for details on the simulation procedure. Briefly, we did not simulate ground truth genotypes, nor have we performed imputation/phasing on simulated data. Instead, we directly simulated ground truth IBD segments (obtained from simulated ARGs), and these are the segments analyzed in Fig.3. While we agree that factors such as phasing and imputation, aDNA damage, coverage, etc., will impact downstream inferences, all of these sources of errors are absorbed into our IBD detection error model. As we have convincingly shown, our error correction model could recover unbiased Ne despite IBD detection errors.

As we have shown in Fig.4, knowledge of the error generating model leads to inference results that are indistinguishable from those with ground truth IBD. Whenever the error model is unknown, the procedures in Supplementary Note S2 show how the model can be inferred. In these procedures, we used real aDNA BAM files and downsampled the BAM files to coverages that match the average coverage of our real-data samples. Therefore, any inaccuracies in aDNA genotyping and IBD detection can be modeled in a way that results in unbiased estimates. For more details on error model misspecification, see our response to question R4. For an in-depth study of IBD detection accuracy from aDNA, see Ringbauer et al. 2024.

R3. How sensitive are the results to the choice of ancIBD parameters? Have the authors tried to vary the parameters of this method and analyse the resulting population trajectories?

As explained in the answer to the previous comment, IBD detection errors, regardless of their root cause, are accounted for using our error model. Therefore, using different ancIBD parameters (or using an entirely different method) will change the precision, recall, and length bias of ancIBD. Therefore, as long as the error profile of the IBD segments is estimated (as described in Supplementary Note S2) and IBD calling performance remains reasonable, unbiased Ne estimates can be obtained.

R4. As remarked by the authors, the IBD estimation errors used when analyzing the simulated data match the true ones, so it is clear that the corrections should improve the estimation of Ne. The authors should investigate this thoroughly - are the error rates estimated indeed usable in other scenarios? A priori my guess is that they are a function of various factors (demography, coverage, etc, not least the IBD detection algorithm itself), and thus estimates on one datasetand Bose from another. Thus using them in any study will bias results in ways which are unclear and not understood at all. The authors should look into this, and perhaps estimate the error rates on unrelated data with different demographies and check how this impacts their inference of Ne. If this does indeed work independently of the simulated demographies/coverage/IBD detection algorithm/etc then this would prove to be a rather useful tool to account for IBD detection errors.

We did not want to imply that the estimated error rates are “usable in other scenarios” and could “work independently of the simulated demographies/coverage/IBD detection algorithms etc”. Instead, the estimated error model is specific to coverages, IBD detection algorithms, etc. (as highlighted by the reviewer). In our manuscript, we estimate the error models separately for the Corded Ware samples and the British samples. In both cases, we estimated the error model using simulated genomes whose coverage match the average coverage of the empirical samples. We have added an explicit description in Section 1.8 to clarify this approach.

L330 - L334:
To estimate error models for IBD detection, we first computed the average coverage of 1240k and WGS data in the empirical samples and then simulated IBD (described in detail in Supp. Note S2) by downsampling 1240k/WGS BAM files to the average coverage, and used the average of the estimated 1240k and WGS error models for the empirical data analysis.

We acknowledge that the ancestry of our empirical data (Corded Ware and Medieval British samples) does not match the precise ancestry of the samples used for estimating error models, for which we have used a mix of Neolithic, Yamnaya, and Bellbeaker samples. We stress that in downsampling simulations, we are limited to samples with high coverage, and we also need a parent-offspring pair to simulate ground truth IBD. Due to this constraint, a perfect match in ancestry to Corded Ware and Medieval Britain is not attainable. However, we do not think this ancestry mismatch will cause much bias in estimated errors because benchmarks on ancient DNA imputation (Fig 2a,c in da Mota et al.) have shown that imputation accuracy is similar across these Eurasian ancestries when using the 1000 Genome reference panel and imputation quality is the primary determinant of IBD detection error in aDNA (Ringbauer et al. 2024).

We agree that the point of misspecified error models is an important issue. Therefore, we have now conducted additional simulations to explore whether certain levels of misspecification introduce substantial biases. We first estimated error models on WGS data downsampled to 0.25x, 0.5x, 1x, and 2x and then simulated IBD detection errors using these four error models. We then inferred Ne using error models that did not match the simulated error model. We report our findings about error model misspecification in the main text:

L386 - L389:
We investigate the effects of misspecified error models in Supp Note S4. We found that it mainly affects the absolute value of the inferred Ne trajectory. However,the misspecified model can still recover the qualitative population dynamic (e.g., whether the population size is growing or remains constant).

Additionally, we note that:

L334 - L338:
Given that our estimated Ne has the same order of magnitude as Ne estimated from linkage disequilibrium (LD) using hapNe-LD [Fournier et al., 2023] (Fig.S29, Fig.S34) and the observation that the inferred Ne trajectory is robust to modest misspecification of the error model, we believe that our approach is not substantially biased.

Discussion
D1. Sentence on l. 416 does not end?

Thank you for spotting this. We have fixed this typo.

Supplementary Material
S1. l.12 : Justification with regards to the choice of beta? Plots to show it doesn't affect much?

We refer to our response to question M17.

S2. l.53-59 : A brief explanation of the FPR estimation should be included, not just a citation.

We now describe details on FPR estimation in Supp Note S2.1

S3. l.93-98 : ``To calculate length bias, for each simulated groundtruth IBD, we recorded inferred IBD segments that cover at least half of the groundtruth segment (therefore, there can only be one unique inferred segment satisfying this condition, if it exists at all). The length bias is the length difference between the inferred and the groundtruth segments. If no such inferred segment exists, then this ground truth segment is considered not inferred, and no length bias is recorded."
- This seems arbitrary, and does not account for the segments that IBD routines call but overlap the true IBD regions marginally/not at all. Surely those segments that are called but do not correspond to a true segment should have a length bias equal to their inferred length? Otherwise $R(z|y)$ is effectively being truncated over $(0.5*y, 1.5*y)$?

Indeed, this scheme effectively truncates R(y|z) with a lower bound 0.5z, but there is no upper bound (not 1.5y; for example, one can infer the whole chromosome as one IBD and that surely covers at least half of the ground truth segment). We note that if a ground truth segment does not correspond to any inferred IBD that spans at least half of its length, then the true IBD segment will be considered as “missed”, which is then accounted for by the recall calculation. We also note that for the IBD length range (8-20cM) and aDNA coverage under consideration in our empirical data analysis, the recall of ancIBD is very high (at least 95%, see Fig.S3b). So, for the majority of the ground truth IBD, their length bias can be accurately quantified.

Regarding:“those segments that are called but do not correspond to a true segment”. Those segments are, by definition, false positives, so they are accounted for by the estimated false positive rate.

One difficult case arises when a long segment is inferred to be multiple adjacent but short segments, and in cases of at least two erroneous gaps, potentially none of these short segments are at least half the length of the ground truth segment. We think this error is not straightforward to model under the current

framework. However, as this only happens rarely (see Fig.S3b) and ancIBD merges spurious gaps, we believe the current model is sufficient to substantially reduce biases caused by such IBD detection errors.

S4. l.116-118 : ``Similar to Supp. Note S2.2, a ground truth IBD segment is considered to be inferred if an inferred segment covers at least half of the true IBD segment"
- This is unduly generous - why not use the proportion of genomic length (say in cM) that the inferential scheme calls as a measure of recall? Using the approach proposed in the manuscript can lead to a non-negligible inflation in the recall being reported - in the extreme case that all predicted segments overlapped the true ones by the bare minimum (i.e. 50\%), the true recall (i.e. the actual proportion detected) should be 0.5 whilst the reported recall using the author's approach ends up being 1.

The recall is defined this way to be compatible with Eq.17. The key point is the separate modeling of the length bias. This way, if a true segment is covered by only 50% of its true length, it will be considered as being called, but the discrepancy will be modeled as a length bias. In other words, the length bias models any deviations in the proportion of the genomic length of IBD segments. The recall guarantees proper modeling of the segment count.

Minor comments
1. The acronym IBD is used l.52 but is only introduced in l.67.

Thanks for spotting this. We now introduce the acronym when it was first introduced.

2. Ne on l.62, N_e on l.66, choose one and stick to it.

The expression Ne in L66 is part of the software package name (*TTNe*), which we prefer not to typeset as a mathematical expression.

3. Citation for the choice of generation time being 29 years?

We added three relevant references to demonstrate that this is a common choice in population genetics:

https://pubmed.ncbi.nlm.nih.gov/12721957/ (Helgason et al., 2003)
https://pubmed.ncbi.nlm.nih.gov/10677323/ (Tremblay et al., 2000)
https://pubmed.ncbi.nlm.nih.gov/27140627/ (Moorjani et al., 2016)

4. l.349 is a missing citation.

We have now fixed this error.

Review 3

Recap of submission

The authors introduce a new pipeline called Time-Transact Ne (TTNe) to model time-transact sampling for the purpose of inferring the recent trajectories of Ne (effective population sizes). The data are identity-by-descent (IBD) segments from DNA expressed in time series. They then perform a simulation study where they analyze three different demographic settings to validate their approach. They also use TTNe for an empirical analysis.

To the best of my knowledge, the authors provide a literature review that is cognizant of the existing methodology and provide a new method to enhance the study of inheritance under different demographic scenarios. The simulation study seems to well-validate their approach for the examples provided as seen in displays like Figure 3, though I have a few technical concerns that I would like to see addressed.

Major comments

Line 195, equation (12): The Bayes' rule is applied for the probability distribution conditioned on Ne, so both lines on the right-hand side of (12) should have P(\ell|T = t, Ne) instead of P(\ell|T = t). Was it stated/proven somewhere that P(\ell|T = t) actually equals P(\ell|L = l,Ne)?

Once t, the time depth of the common ancestor is fixed, the IBD segment length distribution is independent of N_e(shown in Palamara et al., 2012). We now describe this explicitly in the main text.

L255 - L258:
We note that conditioning on a fixed time t to the most recent common ancestor, the IBD length distribution is independent of N_e because the distance to the next recombination is fully determined by 2t meiosis as shown in Palamara et al., 2012.

Minor comments

Lines 84-86: Each (not bold) Ne is taken to be a function over time. It may be a good idea to emphasize how time-dependence is notated here.

Thank you for this suggestion; we have now made this time dependency explicit in L90 - L91:
We use the bold font N_e to denote a vector of float-point values representing $N_e(t)$ at each generation t backward in time.

Line 89: Must the samples S_1,...,S_n be such that t_i < t_j whenever i < j? This seems to be a conventional assumption that is affirmed in the likelihood formula in equation (6), but it might be worth it to say here that t_1 < t_2 < ... < t_n.

In our implementation, we use this time ordering of samples, but there is, in principle, no restriction on the relative age of t_i, t_j. As seen from Eq.2, only the absolute value of the time difference matters in our calculations.

November 14, 2024
RE: GENETICS-2024-307622

Dear Dr. Huang:
I am now pleased to accept your manuscript entitled "Estimating effective population size trajectories from time-series Identity-by-Descent (IBD) segments" for publication in GENETICS, pending minor revision.

I have read the revised manscript carefully, and appreciate the good work that you have put in here, both originally and in response to the reviewers' comments. It's a technically very solid paper describing a useful tool. However, while reading I ran across a few relatively minor issues (see comments below) that should be addressed before final acceptance.

Please submit your revision along with a brief description of how you modified the manuscript in response to these concerns and suggestions (which can be viewed at the bottom of this email. Mostly, these are some minor proofreading bugs (and, please do a careful proofreading pass of your own), but there is one issue that I believe affects the software as well (in a minor way). I think that (I am right and) this is easy to fix, but if I am wrong on either point, please let me know. I expect you should be able to submit a revised manuscript within 30 days. A suitably revised manuscript will be acceptable for publication; I don't expect to send it out for review.

When revising the ms., please make an effort to shorten it, because that almost always improves a manuscript. We urge authors to heed the advice of Strunk and White: "omit needless words"1. Follow this link to submit the revised manuscript: Link Not Available

Thank you for submitting this story to Genetics.

Sincerely,

Peter Ralph
Associate Editor
GENETICS

Approved by:
David Begun
Senior Editor
GENETICS

Associate Editor comments:

eqn (15): Using this equation ignores the contributions from the ends of the chromosomes; a more accurate calculation would be done using f_K( ). To do the calculation, just replace P(l|T=t) with f_K(l,t); this puts in the numerator the expected number of blocks of the length from age t; and in the denominator the total expected number of blocks. This is the right calculation under a Poisson process model of blocks thanks to the 'labeling' property of Poisson processes; as far as I know there is no clean way to do this calculation without making a Poisson assumption. You're right that the cited papers use the expression given; it would be helpful to acknowledge (and, help resolve) this confusion in the literature.

l.90: "floating-point values"

l.102: "Morgans"

l.113: do not indent

l.120: You "sum" instead of "integrate" over t.

(I realize these can be viewed as the same thing, but this might still be confusing.)

l.129: This is a very good explanation of (2)!

l.136: here "waiting time" is actually a distance, which might be confusing given the 'time' context

l.147: I think I would call this a "coalescence probability", not a "rate"?
(since usually we say "instantaneous coalescence rate", which refers to 1/2Ne(t), the "hazard rate"?)

l.154: missing some words in "estimate constant population size"

l.157: why "chromosome 3" (is it human chromosome 3? in terms of length? recombination map?)

l.160: please cite tskit's zenodo record? https://zenodo.org/records/8425430

eq'n (6): Note this is an approximation because you're using f_N() evaluated at the midpoint of the length bin.
This also contradicts the discussion on l.169 which has the left endpoint instead of the midpoint.
(also btw, if you'd been working with the cumulative
distribution instead of the density you could just take differences instead of approximating)

eqn (10): here you use N(x|mu,sigma), but then you define only N(mu,sigma) (without the "x|").
Also, wouldn't it be more clear if you just wrote exp(-(1/2)*(3t/Tmax)^2) instead of this expression?
(You can keep the following explanation in terms of Gaussians.)

l.224: remove indent and put space before "["

l.240: missing ")"

l.247: remove indent

l.314: "GLIMPSE1" not "GLIMIPSE1"

l.353: "We sampled at t=0,15,45": please write this out; for instance, units are in 'generations ago'.

l.454: units on "500 to 1300" ("CE"?)

Associate Editor comments:

eqn (15): Using this equation ignores the contributions from the ends of the chromosomes; a more accurate calculation would be done using f_K( ). To do the calculation, just replace P(l|T=t) with f_K(l,t); this puts in the numerator the expected number of blocks of the length from age t; and in the denominator the total expected number of blocks. This is the right calculation under a Poisson process model of blocks thanks to the 'labeling' property of Poisson processes; as far as I know there is no clean way to do this calculation without making a Poisson assumption. You're right that the cited papers use the expression given; it would be helpful to acknowledge (and, help resolve) this confusion in the literature.

Thank you for spotting this issue. Indeed, the original Eq.15 is an approximation that does not consider chromosome edges. We have now noted in our manuscript that P(l|t) is exactly f_K(l,t) (l.228 - l.229). Because the exact calculation depends on the length of the autosome under consideration, we keep using the approximation (which is independent of the autosome length) in our software implementation. To justify this approximation, we now show that the two calculations give almost identical numerical results (Fig.S20).

l.90: "floating-point values"

We have fixed this error.

l.102: "Morgans"

We have fixed this typo.

l.113: do not indent

We have removed the indentation.

l.120: You "sum" instead of "integrate" over t.
(I realize these can be viewed as the same thing, but this might still be confusing.)

We now write “... is obtained by integrating (or approximated by performing infinitesimal summation) the density over an interval”.

l.129: This is a very good explanation of (2)!

We appreciate your positive feedback!

l.136: here "waiting time" is actually a distance, which might be confusing given the 'time' context

We now write “the distance to the next recombination event” (l. 128).

l.147: I think I would call this a "coalescence probability", not a "rate"?

(since usually we say "instantaneous coalescence rate", which refers to 1/2Ne(t), the "hazard rate"?)

We agree that “coalescence probability” is more appropriate here and have updated our text accordingly.

l.154: missing some words in "estimate constant population size"

We removed this sentence as part of our updates to tighten the manuscript. Estimating a constant population size is only used much later in section 1.3, and we already explain it there.

l.157: why "chromosome 3" (is it human chromosome 3? in terms of length? recombination map?)

We chose human chromosome 3 because it is relatively long and does not contain large regions with very few SNPs (such as chromosome 1’s centromere, which spans almost 10 cM).

l.160: please cite tskit's zenodo record? https://zenodo.org/records/8425430

We now cite tskit’s zenodo record.

eq'n (6): Note this is an approximation because you're using f_N() evaluated at the midpoint of the length bin.
This also contradicts the discussion on l.169 which has the left endpoint instead of the midpoint.
(also btw, if you'd been working with the cumulative
distribution instead of the density you could just take differences instead of approximating)

Thank you for pointing this out. We now use the midpoint in our method description throughout so that it matches with our code.

eqn (10): here you use N(x|mu,sigma), but then you define only N(mu,sigma) (without the "x|").
Also, wouldn't it be more clear if you just wrote exp(-(1/2)*(3t/Tmax)^2) instead of this expression?
(You can keep the following explanation in terms of Gaussians.)

We have now defined $\mathcal{N}(\cdot|\mu,\sigma)$ as the density of Gaussian with mean μ and std σ. We have also written out the exponential in Eq10.

l.224: remove indent and put space before "["

We have fixed this.

l.240: missing ")"

We have corrected this typo.

l.247: remove indent

We have removed the indentation.

l.314: "GLIMPSE1" not "GLIMIPSE1"

We have fixed this typo.

l.353: "We sampled at t=0,15,45": please write this out; for instance, units are in 'generations ago'.

We have now added “generations ago” where applicable.

l.454: units on "500 to 1300" ("CE"?)

We have added “BCE” and “CE” to calendar dates throughout.

Finally, we fully agree with your suggestions that a more focused text can hugely improve its readability. Therefore, we have conducted inline edits throughout our manuscript, aiming to tighten our writing where possible. Those changes are only cosmetic and did not alter any content.

December 9, 2024
RE: GENETICS-2024-307622R1

Mr. Yilei Huang
Max-Planck-Institut fur evolutionare Anthropologie
Department of Archaeogenetics
Deutsch Pl 6
Leipzig
Germany

Dear Dr. Huang:

Congratulations! We are delighted to inform you that your manuscript entitled "Estimating effective population size trajectories from time-series Identity-by-Descent (IBD) segments" is acceptable for publication in GENETICS. Many thanks for submitting your research to the journal.

Thanks very much for addressing the remaining comments. I have no further corrections, but encourage the authors to read the text carefully one more time before sending in the final version (perhaps paying special attention to new bits written recently) to help catch any remaining grammatical errors (for instance, discrepancies in plural-ness, etcetera).

To Proceed to Production:

1. Format your article according to GENETICS style, as discussed at https://academic.oup.com/genetics/pages/general-instructions, and upload your final files at https://genetics.msubmit.net.

2. Your manuscript will be published as-is (unedited-as submitted, reviewed, and accepted) at the GENETICS website as an Advanced Access article and deposited into PubMed shortly after receipt of source files and the completed license to publish. Please notify sourcefiles@thegsajournals.org if you do not wish to publish your article via Advanced Access.

3. We invite you to submit an original color figure related to your paper for consideration as cover art. Please email your submission to the editorial office or upload it with your final files. You can submit a small-sized image for evaluation, and if selected, the final image must be a TIFF file 2513px wide by 3263px high (8.375 by 10.875 inches; resolution of 600ppi). Please avoid graphs and small type.

If you have any questions or encounter any problems while uploading your accepted manuscript files, please email the editorial office at sourcefiles@thegsajournals.org.

Sincerely,

Peter Ralph
Associate Editor
GENETICS

Approved by:
David Begun
Senior Editor
GENETICS

note: Please add jnls.author.support@oup.com and genetics.oup@kwglobal.com (or the domains @oup.com and @kwglobal.com) to your email program's "safe senders" list. You will be contacted by both at various points during the production process.
